# 2240 nm NIR-IV Photodynamic Therapy Can Reverse Ineffective Anti-OX40 Cancer Immunotherapy to Become Effective

**DOI:** 10.1021/acsnano.5c04323

**Published:** 2025-10-11

**Authors:** Munusamy Shanmugam, Chi-Shiun Chiang, Kuo Chu Hwang

**Affiliations:** † Department of Chemistry, 34881 National Tsing Hua University, Hsinchu 30013, Taiwan R.O.C; ‡ Department of Biomedical Engineering and Environmental Sciences, National Tsing Hua University, Hsinchu 30013, Taiwan R.O.C

**Keywords:** NIR-IV photodynamic therapy, immunotherapy, anti-OX40, antitumor immunity, melanoma carcinoma, immunogenic cell deaths

## Abstract

The discovery of immune checkpoint inhibitor (ICI) therapies was granted the Nobel Prize in 2018. However, ICI immunotherapies were later found working poorly for the large majority (70–80%) of cancer patients. It is an urgent need to develop a strategy to conquer this grand challenge and reverse otherwise ineffective immunotherapies to become effective. Herein, we propose a “Blind T cells” model to well rationalize the course leading to the ineffectiveness of immunotherapies. We demonstrate an effective strategy to conquer the ineffectiveness of immunotherapies via producing a large amount of newly generated tumor-recognizing cytolytic CD8^+^ T cells before the administration of immunotherapy reagents. We apply a NIR-IV photodynamic therapy, mediated by LaB_6_–PEG–folate nanoparticles using 2240 nm NIR light excitation, to generate reactive oxygen species, kill cancer cells, in situ produce whole cancer vaccines for priming of CD8^+^ T cells, and induce immunogenic responses in the presence of immunomodulator anti-OX40. A multifunctional anti-OX40 agonist could co-stimulate naive T cells to proliferate with tumor-recognizing properties, as well as suppress the activities of immunosuppressive Treg, and M2-phenotype macrophages, resulting in the complete disappearance of the primary melanoma tumor (that exposes to NIR light irradiation) as well as the effective suppression of remote/metastatic tumors’ growths in the lung (that did not receive photo-irradiation).

## Graphical abstract

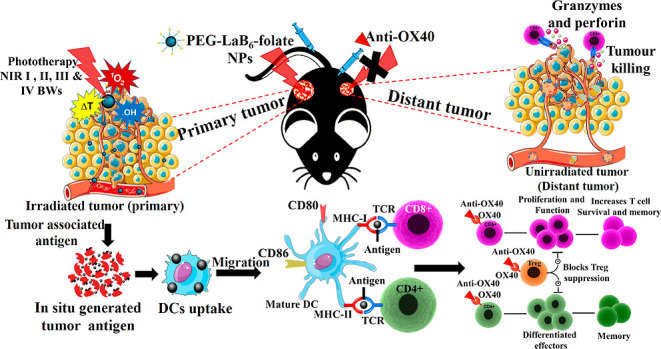

## 1 Introduction

Cancer remains one of the life-threatening diseases for human beings. According to the World Health Organization, the number of cancer deaths worldwide was ∼9.7 million in 2022. Cancer immunotherapy by activating the host immune system to fight against tumor metastasis and recurrence has emerged as an important therapeutic strategy in recent years.^1^ An immune checkpoint inhibitor (ICI) therapy using anti-programmed cell death protein 1 (anti-PD-1/PDL-1) or anticytotoxic T lymphocyte-associated protein 4 (anti-CTLA-4) has recently demonstrated the importance of the antitumor immune activation for cancer treatment.^1^ However, despite the remarkable success of immune checkpoint blockade inhibitors in the treatments of some cancer patients, ICI therapy was found to work well only for 20–30% of cancer patients bearing nonsmall cell lung cancer, melanoma, or renal cell carcinoma but does not work well for a large majority of others.^2^ In clinical trials, cases with the long-lasting effect of ICI therapy are still very rare due to the concerns over induced inflammation and autoimmunity which can be very severe, thus limiting the use of ICI therapies. The reasons for the inconsistent and diverse results of ICI therapy remain elusive. Previously, several plausible mechanisms were proposed to rationalize the causes contributing to the failure of ICI therapies, including T-cell exhaustion in the tumor microenvironment (TME),^3,4^ lack of neoantigens,^5,6^ and immunosuppressive tumor microenvironment (TME).^7,8^ These prior literature works pointed out some factors indirectly contributing to the failure of most immunotherapies but did not identify the key principal factor which is directly responsible for the ineffectiveness of most immunotherapies. It is urgently needed to identify the principal factor responsible for the failure of most immunotherapies and an effective strategy to overcome the grand challenge and to render those ineffective immunotherapies to become effective.

In the oncology community, it is commonly believed that the removal of the immune “brake” of T cells should lead to a dramatic boost of the antitumor immunity. Such a belief is based on a wrong perception/assumption that all antigen-specific T cells can recognize cancer cells. In fact, only a very small fraction of T cells receive training education from those dendritic cells (DCs), which happen to pick up a very limited amount of dead cancer cell debris from a growing tumor. The amount of dead cancer cell debris is very scarce at the early and middle stages of tumor growths but will become more abundant at the late stages of a cancer development. Without the pick up and presentation of dead cancer cell debris by DCs, most of the antigen-specific T cells, in fact, are “blind” and do not recognize cancer cells. Under such a condition, removing the molecular “brake” of those “blind” T cells using immune checkpoint blockade inhibitors not only does not help the improvement of the antitumor immunity but will also result in severe autoimmune-related adverse effects (irAEs).^9,10^ Immunotherapy, especially for these immune checkpoint blockade inhibitors, can work well only when the host system has already had a large number of T cells that recognize the features of cancer cells. To improve the overall antitumor immunity, it is of paramount importance to increase the amount or proportions of tumor-recognizing cytotoxic T cells first before the administration of immune checkpoint blockade inhibitors. It is those tumor-recognizing T cells that can kill cancer cells. To produce a large amount of tumor-recognizing new T cells in a cancer patient, it is necessary to provide large amounts of tumor-associated neoantigens externally or internally for priming and expansion of new tumor-recognizing T cells.

In this work, we apply an unprecedented NIR-IV photodynamic therapy to kill cancer cells, induce inflammation responses, generate whole cancer cell vaccines in situ to stimulate priming of T cells, and use anti-OX40 to co-stimulate proliferation and expansion of tumor-recognizing new T cells, and inhibit the activities of immunosuppressive Treg and M2-phenotype macrophages. A combination of NIR-IV photodynamic therapy and immunomodulator anti-OX40 can produce large amounts of new tumor-recognizing cytotoxic T cells. We found that immunomodulator anti-OX40 alone has a modest effect on the suppression of the tumor growths due to limited amounts of tumor-recognizing T cells. A combination of anti-OX40 immunomodulation with in situ generated whole cancer cell vaccines from NIR-IV PDT has very dramatic and synergistic effects on suppressing tumor growths as well as prolonging the average lifespans of mice-bearing melanoma cancer. The effectiveness of producing tumor-associated neoantigens of phototherapies is in the following order: NIR-IV PDT > NIR-III PDT ≫ NIR-II PDT > NIR-I PTT. In in vivo mice experiments, the combination of treatments LaB_6_–PEG–folate NPs with 2240 nm and anti-OX40 could remarkably extend the average half-life span of mice-bearing B16BL6 malignant melanoma cancer to an amazingly long 83 days (LaB_6_–PEG–folate NPs + 2240 nm + anti-OX40), which is much longer than those from other treatment groups, including, 76 days from the (LaB_6_–PEG–folate NPs + 1550 nm + anti-OX40)-treated group, 51 d from the (LaB_6_–PEG–folate NPs + 1064 nm + anti-OX40)-treated group, 43 d (LaB_6_–PEG–folate NPs + 808 nm + anti-OX40), 21 days from the immunotherapy reagent anti-OX40 alone-treated group, as well as the dark control group (17 d, dark and the absence of LaB_6_ NPs).

## 2 Results

### 2.1 Preparation and Characterization of LaB_6_–PEG–Folate NPs

Lanthanum hexaboride nanoparticles (LaB_6_ NPs) were synthesized by following a literature procedure with a slight modification using anhydrous lanthanum chloride and sodium borohydride as precursors.^11^ The choice of LaB_6_ NPs is the extension of our earlier work in which LaB_6_ NPs are able to absorb long wavelength NIR light and generate ROS to exert photodynamic therapeutic effects.^12–14^ The scanning electron microscopy (SEM) (Figure S1a) shows a uniform size distribution of the as-synthesized LaB_6_ NPs. X-ray diffraction (XRD) analysis (Figure S1b) confirms the crystallinity of the LaB_6_ NPs with major reflection peaks well matching with those shown in JCPDS files (LaB_6_ NPs: JCPDS card No: 65-1831).^15^ UV–vis–NIR spectrophotometer analysis (Figure S1c) reveals the broad absorption spectrum of LaB_6_ NPs extending up to 2500 nm, covering the NIR-I to NIR-IV biological windows. To introduce the tumor-targeting ability, the as-prepared LaB_6_ NPs were functionalized with citric acid, followed by further chemical functionalization with the NH_2_–PEG–folate moiety via 1-ethyl-3-(3-(dimethylamino)­propyl) carbodiimide (EDC) coupling to form LaB_6_–PEG–folate NPs.^16^ To validate the successful conjugation, FTIR analysis was performed and shown in Figure S2.^7^ The FTIR spectrum of citrate-LaB_6_ NPs shows characteristic peaks at 1730 cm^–1^ (ν_C=O_ stretching of the surface-bound carboxylic acid), while the successful conjugation of LaB_6_–PEG–folate was confirmed by the appearance of the amide bond C=O stretching at 1632 cm^–1^ and the N–H stretching peak at 3300 cm^–1^, respectively. The 2855 cm^–1^ and 2924 cm^–1^ peaks are due to the C–H bond stretching from the PEG moieties. The molar extension coefficient for the as-synthesized LaB_6_–PEG–folate NPs were measured to be 0.74 × 10^10^ M^–1^ cm^–1^ @ 808 nm, 0.91 × 10^10^ M^–1^ cm^–1^ @ 1064 nm, 0.95 × 10^10^ M^–1^ cm^–1^ @ 1550 nm, and 0.94 × 10^10^ M^–1^ cm^–1^ @ 2240 nm, which are 5–7 orders higher than conventional organic photosensitizers. Furthermore, the chemical composition of LaB_6_ NPs was determined by X-ray photoelectron spectroscopy (XPS). Figure S1d depicts the full-length survey spectrum of the nanoparticle. The peaks of La and B are from the nanoparticle, while peaks related to O and C are due to the atmospheric air. La 3d^5^ peaks at 838.9 eV (La 3d^5^) and 853.0 eV (La 3d^3^) and the B 1s peak at 188.0 eV, as shown in Figures S1e,f, are in well agreement with the data as reported in the literature thus confirming the LaB_6_ NP structure.^17^ The dark and photostability of the LaB_6_ NPs were also examined to be very stable in physiological environments (see Supporting Information, Figure S3).

### 2.2 ROS Generation from LaB_6_–PEG–Folate NPs upon NIR Light Irradiation

To confirm the generation of ROS species, a LaB_6_–PEG–folate NP-containing aqueous solution was irradiated with NIR-I light (808 nm), NIR-II (1064 nm), NIR-III (1550 nm), and NIR-IV (2240 nm), respectively, in the presence of 100 mM TEMPO spin-trapping reagent and electron paramagnetic resonance (EPR) measurements were performed (Figure 1a,d). From EPR spectra, the characteristic 1:1:1 triplet EPR singlet oxygen signals (^1^O_2_) were observed from the TEMPO spin adduct upon LaB_6_ NPs + 1064 nm excitation (Figure 1a). The singlet oxygen EPR signal was dramatically quenched in the presence of a ^1^O_2_ scavenger, i.e., l-histidine, confirming the identity of the singlet oxygen. No singlet oxygen EPR signals were observed from LaB_6_ NPs in the dark and under 808, 1550, or 2240 nm light excitation (Figure 1a). In addition, the generation of singlet O_2_ upon NIR-II 1064 nm excitation can be directly detected by the characteristic singlet oxygen phosphorescence at ∼1265 nm (Figure S4). Photoexcitation by 808 nm did not show any noticeable singlet O_2_ phosphorescence (Figure S3). While the photoexcitation of LaB_6_ NPs by 1550 and 2240 nm cannot generate singlet oxygen, because the photon energies of both 1550 and 2240 nm NIR light are lower than the band gap energy (0.98 eV, corresponding to 1265 nm) of singlet oxygen. Furthermore, to support the single oxygen generation by 1064 nm photoirradiation, a singlet oxygen specific fluorescence probe, namely, 1,3-diphenylisobenzofuran (DPBF), was used to detect the formation of singlet oxygen. DPBF can react with singlet oxygen to form an endoperoxide intermediate, followed by decomposition to form a nonfluorescent 1,2-dibenzoylbenzene.^18^ As shown in Figure 1b, the fluorescence intensity of DPBF at 410 nm decays continuously proportional to the 1064 nm light irradiation time of a LaB_6_–PEG–folate NPs-containing aqueous solution, which is consistent with the formation of singlet oxygen from LaB_6_ NPs upon 1064 nm NIR light irradiation. While no fluorescence decay was observed upon 808, 1550, and 2240 nm NIR light irradiation (Figure 1b). Such a result is consistent with the EPR experiments that only 1064 nm light irradiation of LaB_6_ NPs leads to the formation of singlet oxygen but not from 808, 1550, and 2240 nm NIR light irradiation. When a singlet oxygen (^1^O_2_) specific sensor (singlet oxygen sensor green, SOSG) was used to detect the formation of singlet oxygen (see Figures 1c and S5), the same conclusion was obtained that only 1064 nm light irradiation of LaB_6_ NPs could lead to the formation of singlet oxygen. Further, the quantum yield (Φ_LaB_6_+1064_) of singlet oxygen from 1064 nm light irradiation of LaB_6_ NPs was determined to be 0.22 (see the Supporting Information for detailed measurements and calculations).

**Figure 1:**
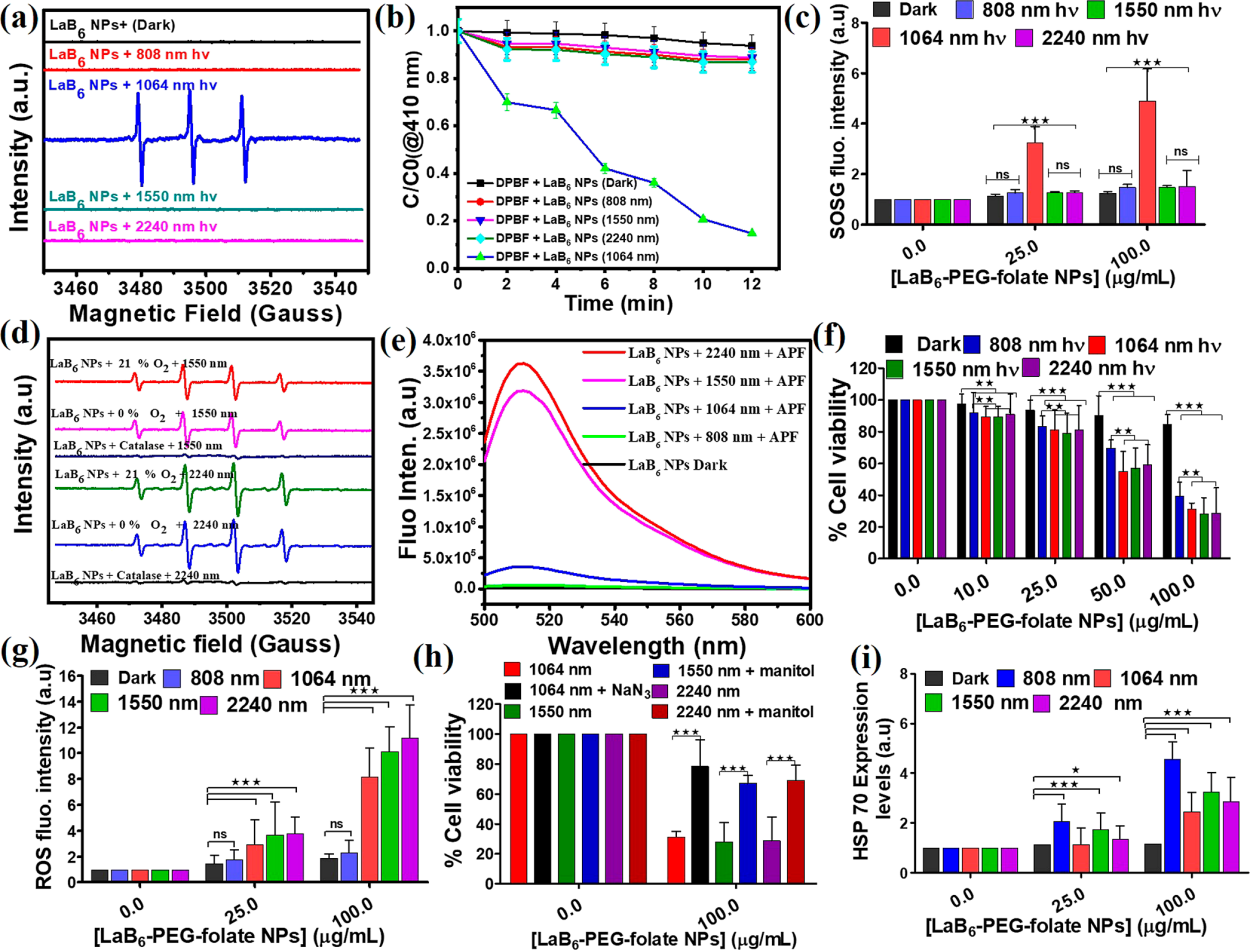
Singlet oxygen and hydroxyl radical detection, in vitro cellular viabilities in B16Bl6 melanoma cancer cells, ROS generation, and HSP70 protein detection upon photoirradiation of LaB_6_–PEG–folate NPs solutions. (a,c) EPR spectra of TEMPO and DMPO-OH generated from photoirradiation of a LaB_6_ NPs-containing aqueous solution. Adsorbance changes using (b) DPDF probe for the detection of ^1^O_2_ generation. (d) EPR detection of hydroxyl radicals in the absence or the presence of O_2_ upon 1550 and 2240 nm NIR light irradiation. (e) APF probe for the detection of ^•^OH generation, (f) cell viabilities of LaB_6_–PEG–folate NPs-internalized B16BL6 cells under dark and photoirradiation conditions at 37 °C, (g) ROS detection by the mean DCF fluorescence using flow cytometry for LaB_6_–PEG–folate NPs-internalized B16BL6 melanoma cancer cells, (h) cell viability in the absence or the presence of mannitol (a hydroxyl radical scavenger)/sodium azide (a singlet oxygen scavenger), and (i) flow cytometry analysis for heat shock protein (HSP70) expression levels of LaB_6_–PEG–folate NPs-internalized B16BL6 cells monitored, respectively. Data are expressed as mean ± s.d. (*n* = 2). One-way ANOVA with Tukey’s posthoc test was used to determine statistical significance. *P < 0.05, **P < 0.01, ***P < 0.001, comparing treatment groups to control and among selected group pairs as indicated.

To determine whether hydroxyl radicals were formed from LaB_6_–PEG–folate NPs under different photoirradiation conditions, 5,5-dimethyl-1-pyrroline *N*-oxide (DMPO) was used as a spin trapping probe to catch the hydroxyl radical.^1^ Upon photoirradiation of a LaB_6_–PEG–folate NPs-containing aqueous solution in the presence of 100 mM DMPO, very strong hydroxyl radical EPR signals with a characteristic 1:2:2:1 quartet pattern were observed upon 1550 and 2240 nm photoirradiation (see Figure 1d), but only a very weak hydroxyl radical EPR signals were observed upon 808 and 1064 nm photoirradiation (see Figure S6). Amazingly, the hydroxyl radical EPR signals could be observed under both normoxia (21% O_2_) and hypoxia conditions (0% O_2_) upon 1550 and 2240 nm NIR light radiation (see Figure 1d). The generation of hydroxyl radicals upon 1550 and 2240 nm NIR light radiation was also confirmed by the use of a hydroxyl radical fluorescence probe, namely, 3′-(*p*-aminophenyl) fluorescein (APF) (see Figure 1e). APF is a nonfluorescent but will be converted to a fluorescent product after reaction with hydroxyl radicals.^19^ A strong fluorescence (λ_max_ = 510 nm, Figure 1e) was observed from the APF-hydroxyl radical reaction product upon 1550 and 2240 nm light irradiation of an aqueous solution containing both LaB_6_–PEG–folate NPs and APF, while no fluorescence was observed upon 808 and 1064 nm light irradiation. The results are consistent with the above EPR observation that hydroxyl radicals were generated from a LaB_6_ NPs-containing aqueous solution upon 1550 and 2240 nm NIR light irradiation, but no or very little amount of hydroxyl radicals were formed upon 808 and 1064 nm NIR light irradiation. The quantum yield values for the generation of hydroxyl radicals from LaB_6_ NPs were determined to be Φ_LaB_6_–2240_ = 0.257, Φ_LaB_6_–1550_ = 0.238, Φ_LaB_6_–1064_ = 0.043, and Φ_LaB_6_–808_ = 0.019, respectively (see the Supporting Information for detailed measurements and calculations). Because two hydroxyl radicals can couple to form hydrogen peroxide (H_2_O_2_), we also determine the formation of H_2_O_2_ using an Amplex red assay kit (Figure S7) under different photoirradiation conditions of a LaB_6_–PEG–folate NPs-containing aqueous solution, including 808 nm (300 mW/cm^2^; 15 min), 1064 nm (300 mW/cm^2^; 12 min), 1550 nm (300 mW/cm^2^; 12 min), and 2240 nm (300 mW/cm^2^; 12 min), respectively. As expected, quite large amounts (3–8 μM) of H_2_O_2_ could be produced upon 1550 and 2240 nm NIR light irradiation of a LaB_6_–PEG–folate NPs-containing aqueous solution within 12 min photoirradiation (Figure S7). The mechanism for the formation of hydroxyl radicals from the photoirradiation of LaB_6_ NPs were reported by our group before.^12^ Briefly, it was reported that water molecules could undergo dissociative adsorption of the surface of LaB_6_ to form proton cations and hydroxide anions.^20^ Upon photoexcitation, the hydroxide anion was oxidized by the hole (most probably, La^4+^ cation) to form hydroxyl radicals, which can further couple to form hydrogen peroxide.^20^ The generation of hydroxyl radicals as ROS via photoirradiation of LaB_6_ NPs upon 1550 and 2240 nm NIR light can be considered as a nonclassical type-III PDT^21^ because it did not generate superoxide (type I PDT) or singlet oxygen (type II PDT) but generates hydroxyl radicals (^•^OH) instead.

### 2.3 In Vitro Photoinduced Cancer Cell Deaths Mediated by LaB_6_–PEG–Folate NPs

To investigate the biocompatibility and the photocytotoxicities of LaB_6_–PEG–folate NPs, in vitro cell experiments were carried using B16BL6 melanoma cancer cells and irradiation with different NIR light. As shown in Figures 1f and S8, the cell viabilities of B16BL6 melanoma cancer cells are weakly LaB_6_ NPs dose dependent in the dark but are highly dose dependent under photoirradiation conditions. Such a result indicates that the dark cytotoxicity of LaB_6_–PEG–folate NPs is low, but the photocytotoxicities are high. The low dark cytotoxicity of LaB6-PEG–folate NPs observed in our study can be explained by their surface functionalization. The citric acid coating enhances the nanoparticle stability and prevents aggregation, reducing nonspecific cellular interactions. The folate modification further improves biocompatibility by enabling targeted delivery to folate-receptor-overexpressed tumor cells. This targeted design minimizes off-target effects and cellular stress under dark conditions, resulting in the high cell viability and low cytotoxicity observed in the absence of irradiation. Roughly, the cell viabilities for ROS-generating wavelengths (including 1064 nm, ^1^O_2_; 1550 nm, ^•^OH; and 2240 nm, ^•^OH) (Figure 1g,h) are lower than 808 nm photoirradiation which does not generate ROS but only heat/hyperthermia generation. In the literature, it is known that mannitol can scavenge hydroxyl radicals and protects plants from oxidation by hydroxyl radicals,^22^ whereas sodium azide is known to be a singlet oxygen specific quencher.^23^ The result suggests that photodynamic therapy, in general, has a better cancer cell killing effect and will generate more dead cancer cell debris than photothermal therapy under the same photoirradiation conditions. Figure 1f also shows that in the presence of a LaB_6_–PEG–folate NPs dose of 50 μg/mL, the dark cell viability is 91%, indicating a good biocompatibility and low dark cytotoxicity of the LaB_6_ nanomaterial. Similar low dark cytotoxicities were observed toward healthy human umbilical vein endothelial cells (HUVECs) (Figure S9). The surface-chelated folate could provide the nanoparticles with a cancer cell targeting ability because folate receptors are overexpressed on most cancer cells.^24^ The surface-chelated folate moieties largely enhance the uptake of the nanoparticles by the cancer cells compared with those without chelating folate moieties (Supporting Information, Figure S10). Under 808 nm NIR light irradiation conditions, the LaB_6_ NPs convert nearly all the incident photon energy to heat and generate a local hyperthermia effect, which is accompanied by the overexpression of the heat shock protein HSP70 (see Figure 1i). Figure 1i also shows that certain fractions of 1064, 1550, and 2240 nm photon energies were also converted (by LaB_6_ NPs) to heat, in addition to ROS generation. The determination of the relative contribution of hyperthermia-induced cell deaths and ROS-induced cell deaths by photoirradiation of LaB_6_–PEG–folate NPs were included in the Supporting Information (Figures S11 and S12).

### 2.4 In Vivo Biodistribution and Antitumor Efficacy of LaB_6_–PEG–Folate NPs

In order to determine the biodistribution of boron and lanthanum in mice, LaB_6_–PEG–folate NPs were intravenously injected into the B16BL6 melanoma tumor-bearing mice. At 12 and 24 h post-i.v. injection, the mice were sacrificed and organs were collected for inductively coupled mass spectroscopy (ICP–MS) analysis. Higher accumulations of B (275 μg/g tissue) and La (75 μg/g tissue) were observed at the tumor site at 24 h after iv injection (Figure S13). Hence, the time point at 24 h post-iv injection was chosen to conduct photoirradiation treatment. The in vivo circulation time plays a key role in the accumulation of nanoparticles at tumor sites.^25^ In vivo pharmacokinetics studies of LaB_6_–PEG–folate NPs and LaB_6_ NPs (without surface modification with PEG–folate) were also investigated using healthy mice. Female C57BL/6J mice were intravenously injected with LaB_6_–PEG–folate NPs and LaB_6_ NPs, respectively, with a dose of 50 mg/kg, and blood samples were collected at various time points (1, 3, 6, 12, 24, 36, 48, and 60 h) after iv injection, followed by ICP–MS analysis. The half-lives in the blood circulation were determined to be ∼20.3 and ∼8.7 h for LaB_6_–PEG–folate NPs and LaB_6_ NPs, respectively (Figure S14). The result clearly indicates that the surface-functionalized PEG–folate moieties can significantly prolong the blood circulation time of LaB_6_ NPs, allowing more time for LaB_6_ NPs to be taken up by tumor tissues. In vivo photothermal images were taken for mice injected with LaB_6_–PEG–folate NPs (50 mg/kg of mice) and then irradiated with NIR light at a time point of 24 h post-iv injection. A thermal camera was used to monitor the temperature changes at the tumor sites under different photoirradiation conditions. As shown in Figures 2a and S15, the local temperature rises to as much as 56.6 and 50.8 °C at the tumor sites were observed after 808 and 1550 nm laser irradiation, respectively, while less temperature rises were observed at 1064 nm (44.9 °C) and 2240 nm (42.9 °C) laser irradiation, respectively. Photoirradiation of LaB_6_–PEG–folate NPs at the tumor site causes a significant rise in the local temperature (Δ*T*). The values of Δ*T* at the tumor sites are as follows: 16.5 °C (808 nm), 7.6 °C (1064 nm), 16.0 °C (1550 nm), and 6.8 °C (2240 nm), respectively (Figure S15). Higher amounts of the temperature rise were observed for 808 and 1550 nm light irradiation as compared to 1064 and 2240 nm NIR light irradiation groups. This result is consistent with in vitro cellular death data (Figure 1f) that 808 nm generate only PTT effect, while photoirradiation using 1064, 1550, and 2240 nm generate combined PDT (major) and PTT (minor) effects on killing cancer cells. In the literature, it is known that necrotic cell deaths could generate more cancer cell debris/neoantigens and thus stronger immune inflammation responses.^26,27^ We, therefore, measured the fractions of apoptotic and necrotic cancer cell deaths resulting from different NIR light irradiation conditions. As shown in Figure S16, photothermal therapy (i.e., photoirradiation of LaB_6_ NPs by 808 nm NIR light)-induced cell deaths occur mostly via combined necrosis and apoptosis pathways with nearly equal fractions, whereas photodynamic therapy-induced cell deaths (i.e., irradiation of LaB_6_ NPs by 1064 nm, 1550, and 2240 nm NIR light) occur mostly via necrosis with a lower fraction of apoptosis pathways. In the literature, it was reported that photodynamic therapy-induced dead cancer cells will release many damage-associated molecular patterns (DAMPs), including ecto-calreticulin (CRT), high mobility group box1 (HMGB1), ATP, etc., which could induce a strong immunogenic response.^28–30^ To confirm that the phototherapies reported in this study could induce strong inflammatory responses, we set up experiments to measure the amounts of DAMPs, including CRT, HMGB1, and ATP. As shown in Figure S17, higher CRT expression levels could be observed in LaB_6_–PEG–folate NPs with laser light irradiation (808, 1064, 1550, and 2240 nm) compared to the dark control group. These results indicate the successful transportation of CRT to the cell surface. Similar increased levels for both ATP and HMGB1 were also observed for LaB_6_–PEG–folate NPs + laser light irradiation (808, 1064, 1550, and 2240 nm) compared to the dark control group, suggesting strong immunogenic cell death (ICD)-induced activation of antitumor immunity. Notice that the 2240 nm NIR light irradiated group has the highest levels of DAMPs among all, which clearly indicates that the NIR-IV (2240 nm) PDT is much more effective in killing cancer cells than other shorter wavelength NIR PDT due to the deeper tissue penetration depths of the longer wavelength NIR light (2240 nm). The percentage of transmitted light output power through 0.5 cm of pork tissue was measured to be 26%/808 nm (NIR-1), 38%/1064 nm (NIR-II), 43%/1550 nm (NIR-III), and 46%/2240 nm (NIR-IV), respectively (see Supporting Information, Figure S18).

**Figure 2:**
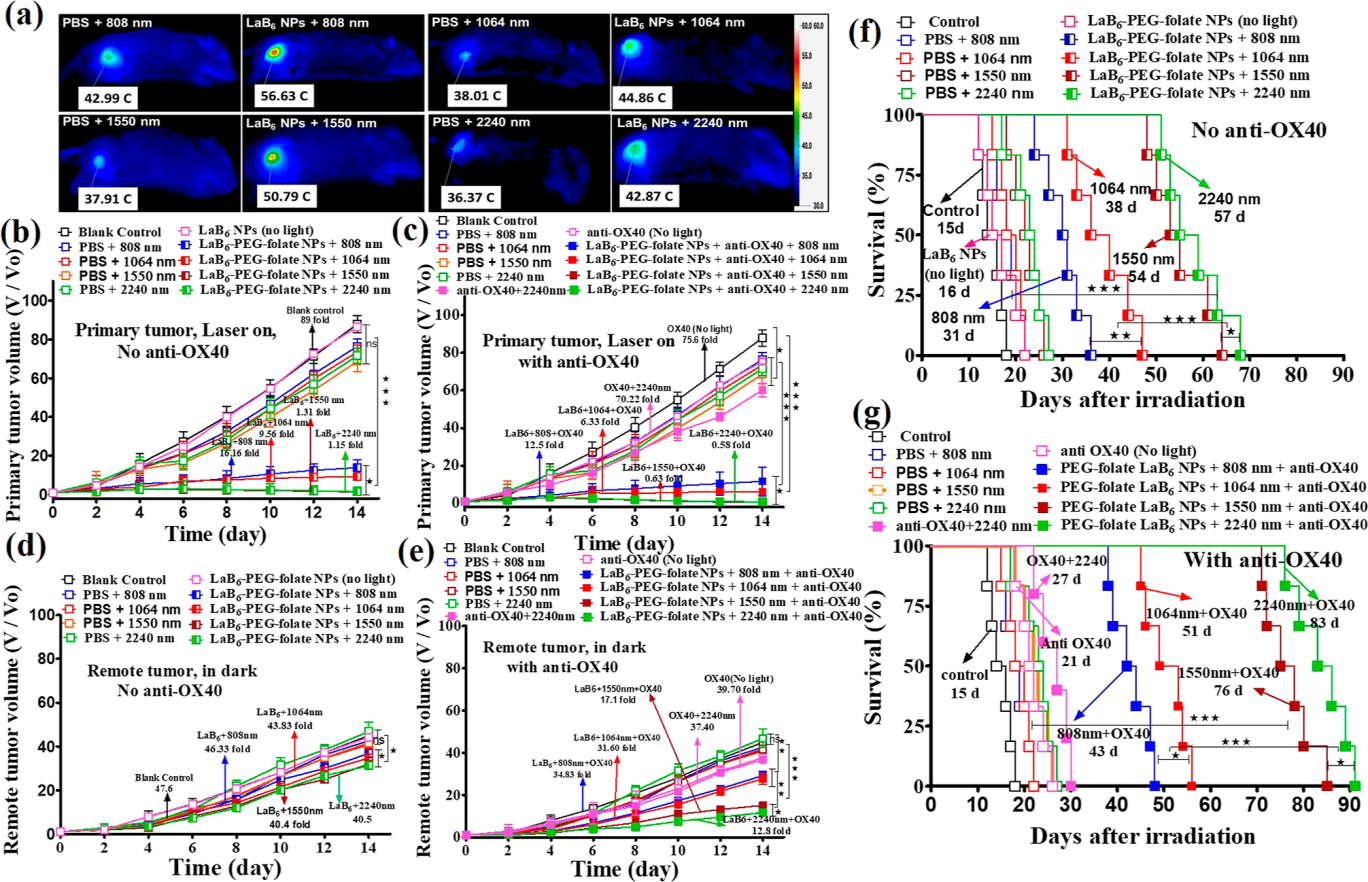
In vivo photothermal images, tumor volume, and average half lifespan measurements. (a) In vivo photothermal images and temperature rises of mice under different photoirradiation conditions, including 808 nm (300 mW/cm^2^, 15 min), 1064 nm (300 mW/cm^2^, 12 min), 1550 nm (300 mW/cm^2^, 12 min), and 2240 nm (300 mW/cm^2^, 12 min). (b) The tumor growth curves of the primary tumors for different treatment groups in the absence of anti-OX40. (c) The tumor growth curves of the primary tumors for different treatment groups in the presence of anti-OX40. (d) The tumor growth curves of the remote tumors for different treatment groups in the absence of anti-OX40. (e) The tumor growth curves of the remote tumors for different treatment groups in the presence of anti-OX40. (f) The survival rates of mice for different treatment groups in the absence of anti-OX40. (g) The survival rates of mice for different treatment groups in the presence of anti-OX40. (*n* = 6 mice per group). Statistical significance was determined using one-way ANOVA followed by the Tukey’s posthoc test. *P < 0.05, **P < 0.01, ***P < 0.001.

For in vivo animal experiments, female C57BL/6J mice were subcutaneously injected B16BL6 melanoma cancer cells into both the right (5 × 10^6^ cells) and the left flanks (5× 10^5^ cells) regions on the same day. The right region was treated as the primary tumor, while the left region was treated as the remote tumor. Then, the mice were randomly divided into 15 groups: including the control, PBS + laser irradiation (808, 1064, 1550, and 2240 nm light irradiation) groups, LaB_6_–PEG–folate NPs + laser (808, 1064, 1550, and 2240 nm) treated groups, anti-OX40 alone-treated group, and LaB_6_–PEG–folate NPs + laser + anti-OX40 (808, 1064, 1550, and 2240 nm) groups. The primary tumors were irradiated with different NIR light, including 808 nm (300 mW/cm^2^, 15 min), 1064 nm (300 mW/cm^2^, 12 min), 1550 nm (300 mW/cm^2^, 12 min), and 2240 nm (300 mW/cm^2^, 12 min), respectively, whereas the remote tumor did not receive phototherapy treatments. The light irradiation times were adjusted to balance the differences in the extinction coefficients so that the amounts of photons being absorbed by LaB_6_–PEG–folate NPs are the same for all wavelengths. To assess the feasibility of lysates as an in vivo whole cancer cell vaccine for the stimulation of host antitumor immune responses, the tumor volume, the survival rates, as well as various immune-related cytokines were measured post-photoirradiation treatments and compared to those control groups in the dark.

Anti-OX40 was injected intraperitoneal to the mice with a dose of 50 μg/mouse on days 1, 4, and 8, whereas LaB_6_–PEG–folate NPs were injected intravenously to mice with a dose of 50 mg/kg on day 0, 2, 5, and 8, followed by photoirradiation at the time point of 24 h post-injection. Subsequently, the tumor volumes were monitored for both the primary (receiving direct photoirradiation) and the remote (without photoirradiation treatments) tumors, respectively. The mice receiving treatment of combined “LaB_6_–PEG–folate NPs + laser irradiation + anti-OX40” shows the effective suppression of tumor growths for both the primary and the remote tumors in comparison with those treated with PBS + laser irradiation. Figure 2b shows the primary tumor growth curves with LaB_6_–PEG–folate NPs + laser irradiation (808, 1064, 1550, and 2240 nm) without the administration of anti-OX40. All photoirradiated groups show very good suppression of tumor growths at the primary tumor (16.2-fold for 808 nm, 9.2-fold for 1064 nm. 1.3-fold for 1550 nm and 1.2-fold for 2240 nm, respectively) but modest effects on the suppression of tumor growths at the remote tumor sites (see Figure 2d). The NIR-III (1550 nm) and NIR-IV (2240 nm) PDT showed a much better suppression on the primary tumor growths as compared to the NIR-I (808 nm) PTT and the NIR-II (1064 nm) PDT-treated groups, which are attributed to the much deeper tissue penetration depths of longer wavelength NIR light and thus better photoexcitation of LaB_6_ NPs at the primary tumor sites. Upon administration of anti-OX40, all photoirradiated groups showed excellent suppression effects on the growth of the primary tumors (12.5-fold for 808 nm, 6.3-fold for 1064 nm, 0.6-fold for 1550 nm, and 0.6-fold for 2240 nm) as compared to 82.6-fold for anti-OX40 alone/no light and 102.8-fold for the dark control group, respectively (see Figure 2c), whereas, unlike in the case of the absence of anti-OX40, the growths of the remote tumors were also effectively suppressed (see Figure 2e) with the remote tumor suppression effects in the following sequence: 2240 nm (tumor size, 12.8-fold) > 1550 nm (17.1 fold) > 1064 nm (31.3 fold) > 808 nm 34.8-fold) > anti-OX40 alone/no light (44.6 fold) > the control (no light/no anti-OX40, 55.6-fold). The results in Figure 2c,e clearly indicate that the immunotherapy reagent anti-OX40 alone has a modest effect on the suppression of both the primary and the remote tumors but has drastic tumor suppression effects on both the primary and the remote tumors upon combination with NIR-III and -IV PDT which can effectively kill cancer cells to provide in situ whole cancer cell vaccines to stimulate the host antitumor immunity as well as the generation of newly produced tumor-recognizing cytolytic T cells (vide infra). The suppression of tumor growths at remote sites solely relies on the activation of the host antitumor immunity. The results in Figure 2e show that 2240 nm NIR-IV light with a longer wavelength has a higher tissue penetration depth and can photo-excite LaB_6_ NPs to generate more ROS and thus has a higher efficacy to in situ produce a larger amount of whole cancer cell debris/vaccine, so that the host antitumor immunity/immunotherapy efficacy could be much better activated in the co-presence of immunotherapy reagent anti-OX40 (vide infra). In other words, the presence of the in situ generated whole cancer vaccine could drastically reverse an otherwise poorly effective anti-OX40-based immunotherapy to become very effective, putatively, via the production of large amounts of newly generated tumor-recognizing neoantigens.

Besides the growth of the primary and remote tumors, the average medium lifespans of different treatment groups were also measured. As shown in Figure 2f, in the absence of anti-OX40, all photoirradiation groups have much longer average half lifespans than the dark control in the following sequence: LaB_6_ NPs + 2240 nm (57 d) > LaB_6_ NPs + 1550 nm (54 d) > LaB_6_ NPs + 1064 nm (38 d) > LaB_6_ NPs + 808 nm (31 d) > dark control (15 d). The results are consistent with those tumor growth data that photoexcitation NIR light with longer wavelengths has a deeper tissue penetration depth and can photoexcite LaB_6_ NPs better to kill more cancer cells, in situ generate more whole cancer vaccines and stimulate better the host antitumor immunity. Upon the administration of anti-OX40 (see Figure 2g), the average medium half-life spans of all photoirradiation groups were further prolonged in the following sequence: LaB_6_ NPs + anti-OX40 + 2240 nm (83 d) > LaB_6_ NPs + anti-OX40 + 1150 nm (76 d) > LaB_6_ NPs + anti-OX40 + 1064 nm (51 d) > LaB_6_ NPs + anti-OX40 + 808 nm (43 d) > anti-OX40 alone/no light (21 d) > dark control (15 d). The results demonstrated that the immuno-co-stimulator anti-OX40 alone has a modest antitumor effect. However, in the presence of whole cancer cell vaccines produced by NIR-I/-II/-III, and, in particular, the NIR-IV PDT, the effects of the immunotherapy reagent, anti-OX40, on suppression of the remote tumor growths were dramatically boosted and become very effective (see data in Figure 2e), which is attributed to the generation and the elevated levels of newly generated tumor-recognizing cytolytic T cells (vide infra). Notably during the treatment, the body weight of mice in all of the treatment groups (Figure S19) was consistent with no fluctuations, suggesting no systemic effect during the treatments.

To further confirm the enhanced immunotherapy efficacy (via combination with in situ-generated whole cancer vaccines) on the inhibition of metastatic tumor growths, 30 mice with normal immune systems were randomly divided into 6 groups including the blank control (*n* = 5), anti-OX40 (*n* = 5), LaB_6_–PEG–folate NPs + anti-OX40 + 808 nm (*n* = 5), LaB_6_–PEG–folate NPs + anti-OX40 + 1064 nm (*n* = 5), LaB_6_–PEG–folate NPs + anti-OX40 + 1550 nm (*n* = 5), and LaB_6_–PEG–folate NPs + anti-OX40 + 2240 nm (*n* = 5), respectively. Besides the primary tumor at the right leg, metastatic tumors were implanted at the lung via the iv injection of B16BL6 melanoma cancer cells (from the mice tail veins) on day 5 after phototherapy treatment at the primary tumor (see Figure 3a). After i.v. injection of melanoma cancer cells and allowing metastatic tumor growths to have 11 days of growth time, the lung organs were removed from one of the mice in each group, followed by the examination of the numbers of metastatic tumor nodules and their distribution in the lung. As shown in Figure 3b, the presence of dark-colored nodules attributed to the melanin-rich B16BL6 melanoma cells provided a qualitative indication of the lung tumor burden across the different treatment groups. This representative analysis (*n* = 1 per group) was conducted solely to illustrate relative differences in metastatic involvement and is not intended for statistical comparison. Consistent with our overall observations, mice treated with anti-OX40 alone showed a relatively higher number of nodules compared with the control group. In contrast, LaB_6_–PEG–folate NPs + anti-OX40 + 808 nm and LaB_6_–PEG–folate NPs + anti-OX40 + 1064 nm exhibited fewer while LaB_6_–PEG–folate NPs + anti-OX40 + 1550 nm and LaB_6_–PEG–folate NPs + anti-OX40 + 2240 nm, qualitatively exhibited no visible nodules. The results are consistent with those results shown in Figure 2, and unambiguously showed that the immunotherapy reagent anti-OX40 alone has very modest antitumor immunity. While in the presence of additional in situ-generated whole cancer cell vaccines (generated by phototherapies), the efficacy of the anti-OX40-based immunotherapy was dramatically boosted, especially for NIR-III and -IV PDT-treated groups. The NIR-III and -IV PDT could effectively kill cancer cells and generate large amounts of tumor-associated neoantigens for priming of newly generated tumor-recognizing T cells via neoantigen presentation by DCs (i.e., the so-called signal I) as well as the stimulation of other immune cells, such as NK cells, macrophages, activated DCs, etc., to secrete the key inflammation cytokine, IL-12, to serve as the signal III for T cells to develop the cytolytic functions and produce antitumor cytokine IFN-γ.^31,32^ Besides, the presentation of a large amount of tumor-associated neoantigens, it was reported in the literature that the presence of a co-stimulatory signal, i.e., the signal II, is mandatory for the proliferation, differentiation, expansion, and survival of T cells.^33,34^ In the absence of the signal II co-stimulation, neoantigen presentation by dendritic cells (i.e., the signal I) alone can only partially activate T cells but no proliferation and differentiation. These partially activated T cells are eventually be deleted. Such a phenomenon is known as T-cell anergy.^35,36^ It was also reported that in the presence of the antigen presentation and co-stimulation signal II but in the absence of signal III (i.e., the stimulation by IL-12 secreted from activated DCs, macrophages, and B cells),^37,38^ T cells can proliferate and expand but are not able to develop the cytolytic effector function and produce antitumor cytokine IFN-γ; that is, these newly generated T cells become tolerant to tumor cells.^31,32^ Therefore, the costimulation of signal III is crucial for these newly generated T cells to develop cytolytic functions.

**Figure 3:**
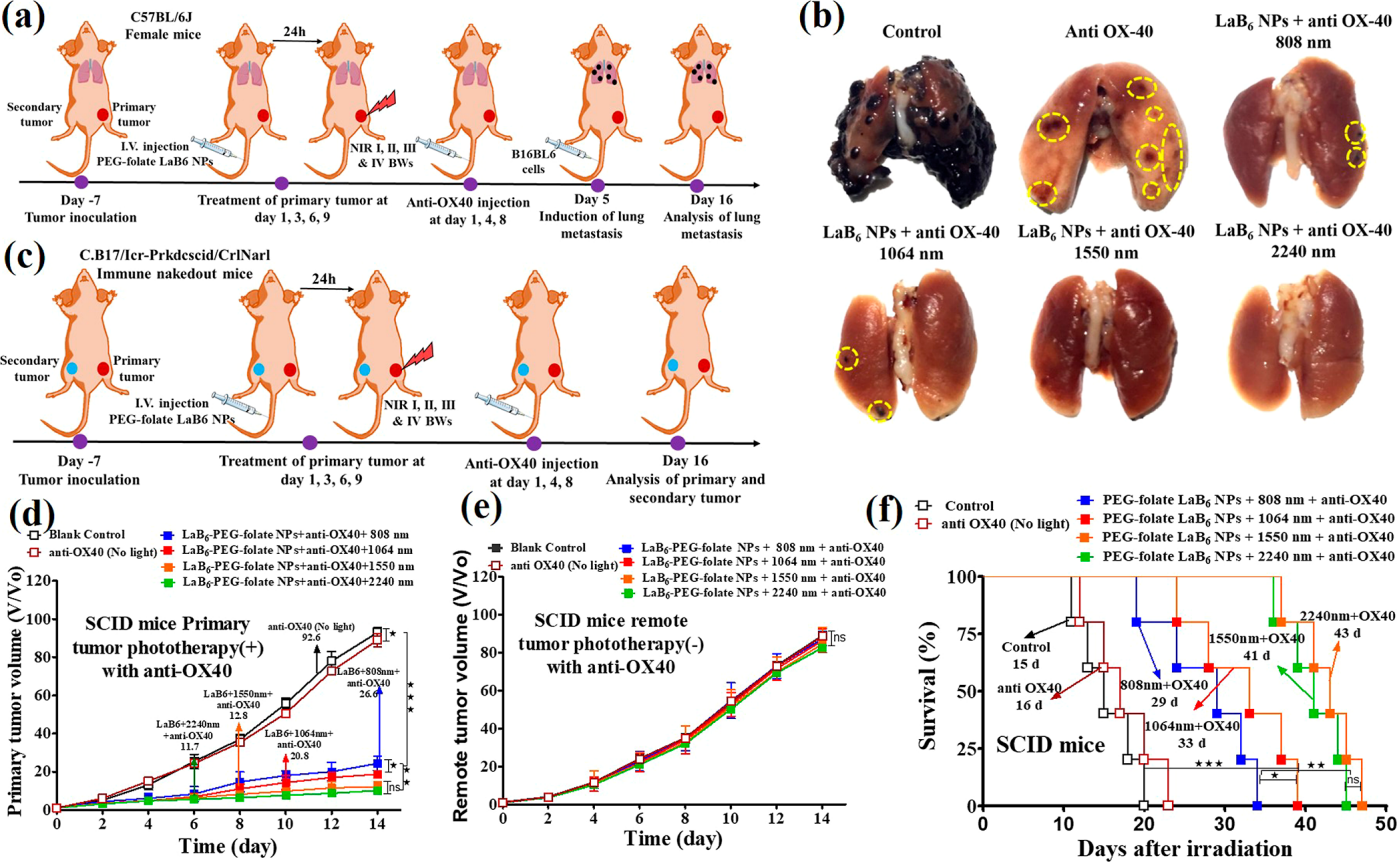
Suppression of metastatic tumor growths in the lung. (a) Protocol for introducing metastatic cancer cells to the lung after phototherapy treatment at the primary tumor using mice with normal immune systems. (b) Representative photographs of lung metastatic nodules of the B16BL6 melanoma tumors for different treatment groups from mice with normal immune systems. Lung images in this panel are from one representative mouse per group (*n* = 1), selected at random, presented for qualitative comparison only, but not as a basis for statistical comparison. (c) Protocol for the treatment of the primary and the remote tumors using immune-deficient SCID mice and administration of anti-OX40, (d) tumor volumes of the primary tumors as a function of time for different treatment groups, (e) tumor volumes of the remote tumors for different treatment groups, and (f) survival rate of mice bearing B16BL6 melanoma tumors and receiving different treatments. The mice used in (a,b) are normal mice, whereas those in (c–f) are immune-deficient SCID mice. (*n* = 6 mice per group). Statistical significance was determined using one-way ANOVA followed by the Tukey’s posthoc test. *P < 0.05, **P < 0.01, ***P < 0.001, comparing treatment groups to the control and among selected group pairs as indicated.

Overall, all signals I, II, and III are mandatory for the priming and generation of new tumor-recognizing cytolytic T cells. Within the tumor microenvironments, various types of immunosuppressive cytokines, such as prostaglandin E2 (PGE2), IL-10, vascular endothelial growth factor (VEGF), and transforming growth factor-β (TGF-β), were secreted by cancer cells, cancer stem cells, Treg, cancer-associated fibroblasts (CAF), and tumor-associated M2-macrophages to suppress the maturation of DCs and the subsequent activation of cytotoxic T cells.^39,40^ Therefore, an external supply of co-stimulatory molecules, such as anti-OX40, to serve as signal II, is very helpful or even necessary for the full activation of T cells. In the absence of large amounts of tumor-associated neoantigens (i.e., signal I), the immunotherapy reagent anti-OX40 alone cannot stimulate the host system to produce large amounts of new tumor-recognizing cytolytic T cells and, thus, has quite a modest effect on enhancing the antitumor immunity.

### 2.5 Confirmation of Boosted Immunotherapy Efficacy on Suppressing Metastatic Tumor Growths Using Immune Knockout SCID Mice

To further confirm that the above boosted immunotherapy efficacy is associated with the production of new tumor-recognizing cytotoxic CD8^+^ T cells, immunodeficient genetically knocked out SCID mice (i.e., the CB17/Icr-Prkdcscid mice, lack of functional T and B cells) were used in this study to repeat the experiments shown in Figure 2. In the immunodeficient SCID mice, the generation of new tumor-recognizing cytotoxic CD8^+^ T cells is not possible, and the above boosted immunotherapy efficacy should disappear if cytotoxic T cells are responsible for the observed high immunotherapy efficacy. CB17/Icr-Prkdcscid mice was subcutaneously injected with B16BL6 melanoma cancer cells into both the right (1 × 10^6^ cells) and the left flank (1× 10^5^ cells) regions on the same day (Figure 3c). On day 7 post-iv injection of the melanoma cancer cells, the primary tumors were irradiated with different NIR lights, respectively. The tumor volumes of the primary and remote tumors were monitored for 14 days. The tumor volumes of the primary and the remote tumors for different treatment groups are shown in Figure 3d,e. Similar to genetically normal mice, the primary tumors which received phototherapies have a noticeable suppression in the tumor growth, whereas the remote tumors did not show a noticeable suppression on the tumor growths, even in the copresence of anti-OX40 as those observed in immune normal mice (see Figure 2e). The results are consistent with the conclusion that the generation of new tumor-recognizing cytolytic T cells is responsible for the boosted immunotherapy efficacy observed in the normal mice systems (i.e., data shown in the Figures 2e,g, and 3b).

The average half lifespan of the immune-deficient SCID mice under different treatment conditions are shown in Figure 3f in the following sequence: 43 d for the (LaB_6_–PEG–folate NPs + anti-OX40 + 2240 nm) group >41 d for the (LaB_6_–PEG–folate NPs + anti-OX40 + 1550 nm) group >33 d for (LaB_6_–PEG–folate NPs + anti-OX40 + 1064 nm) >29 d for the (LaB_6_–PEG–folate NPs + anti-OX40 + 808 nm) group >16 d for anti-OX40 alone-treated group >15 d for the blank control group. Overall, the average half-life spans of the immune-deficient SCID mice groups are much shorter than (∼half of) those of the corresponding normal mice groups under the same treatment conditions (data shown in Figure 2g). Thus, the above results are consistent with the conclusion that newly produced tumor-recognizing cytolytic T cells are responsible for the enhanced effectiveness of anti-OX40 immunotherapy that effectively suppresses the growths of remote metastatic tumors. In short, we have demonstrated here that the in situ generation of new tumor-recognizing cytotoxic T cells could drastically boost the efficacy of an otherwise poorly effective anti-OX40-based immunotherapy in mice having normal immune systems.

### 2.6 In Vivo Antitumor Cytokines (TNF-α, IFN-γ, IL-2, and IL-12) Analysis in Mice with Normal Immune Systems

Interferon-γ (IFN-γ) and tumor necrosis factor-α (TNF-α) are critical mediators of cellular immunity and also play an important role in antitumor immunity involving the activation of natural killer (NK) cells and proliferation of T-cells. Interleukin 2 (IL-2) is an important biomarker in the activation of humoral antitumor immunity.^26^ Interleukin 12 is a cytokine secreted by matured DCs and T helper cells.^41^ IL-12 works by bonding to the IL-12 receptors induces cellular immunity and is mandatory for the development of cytolytic properties of CD8^+^ T cells.^31,42^ To measure the expression levels of the above cytokines after phototherapy treatments, mice with normal immune systems were randomly divided into 15 groups, including the control, LaB_6_–PEG–folate NPs + dark, PBS + laser light irradiation (808, 1064, 1550, and 2240 nm), LaB_6_–PEG–folate NPs + laser (808, 1064, 1550, and 2240 nm), anti-OX40 alone, and LaB_6_–PEG–folate NPs + laser + anti-OX40 + laser light irradiation (808, 1064, 1550, and 2240 nm). After treatments, the expression levels of the above cytokines were examined by collecting the blood samples from a mouse in each treatment group, and measured using ELISA, as shown in Figure 4a–d.^43–45^ We observed no noticeable changes of the expression levels in all TNF-α, IFN-γ, IL-2, and IL-12 from the PBS + laser irradiation group as compared to the dark control group. On the contrast, a significant increase in the serum levels of TNF-α, IFN-γ, IL-2, and IL-12 were observed from the LaB_6_–PEG–folate NPs + laser irradiation + anti-OX40 treatment groups. The results from Figure 4a–d clearly indicate that the serum levels of TNF-α, IFN-γ, IL-2, and IL-12 in the (LaB_6_–PEG–folate NPs + laser irradiation)-treated groups increase noticeably, especially for both the 1550 and 2240 nm irradiation groups. In the co-presence of anti-OX40, the expression levels of all TNF-α, IFN-γ, IL-2, and IL-2 for all (LaB_6_–PEG–folate NPs + laser irradiation + anti-OX40)-treated groups increase very dramatically and all are higher than those from the anti-OX40 alone-treated group. Such a result demonstrated that the presence of large amounts of in situ-generated whole cancer cell vaccines (via NIR-I PTT, and NIR-II/-III/-IV PDT) can largely enhance the efficacy of anti-OX40-based immunotherapy via stimulating the activation and proliferation of the antitumor immune cells (see the green bars in Figure 4a–d). Among the photo-irradiated groups, the NIR-III- and NIR-IV PDT-treated groups could stimulate higher levels of the above-mentioned antitumor-related cytokines as compared to the NIR-I PTT- and NIR-II PDT-treated groups, which is attributed to the deeper tissue penetration depths of 1550 and 2240 nm NIR light, more efficient cancer cell killing effects, and thus a stronger boost of immunotherapy efficacy on the elevation of antitumor immune responses (see also Supporting Information, Tables S1 and S2).

**Figure 4:**
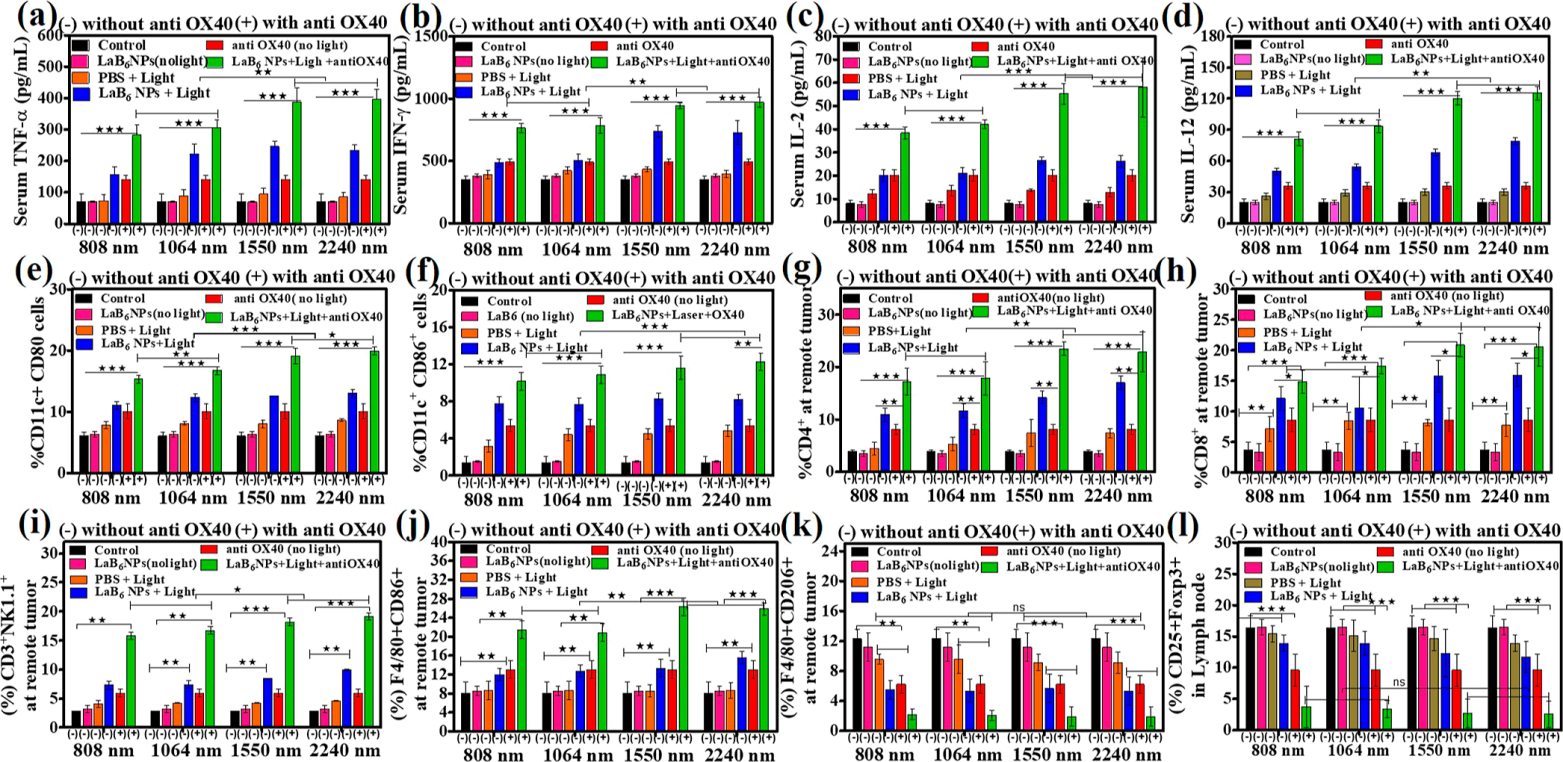
Relative quantification of cytokine levels in the serum for different treatment groups: (a) TNF-α, (b) IFN-γ, (c) IL-2, and (d) IL-12. The maturation level of DCs in the lymph nodes was determined by (e) CD11c and CD80 as well as (f) CD11c and CD86. The populations of (g) CD8^+^CD45^+^ T cells and (h) CD4^+^ and CD45^+^ T cells in remote tumors for different treatment groups. Quantification of proliferation and differentiation of NKT cells were evaluated by measuring the expression level of (i) CD3^+^NK1.1^+^ in the remote tumor. The expression levels of (j) M1-macrophages (CD86^+^) and (k) M2-macrophages (CD206^+^) gating on F4/80 + CD11b^+^CD45^+^ cells at remote tumor sites, as well as (l) Treg cells (CD4^+^CD25^+^Foxp3^+^), respectively, in lymph nodes. (*n* = 2 mice per group). Statistical significance was determined using one-way ANOVA followed by the Tukey’s posthoc test. *P < 0.05, **P < 0.01, ***P < 0.001, comparing treatment groups to the control and among the selected group pairs as indicated.

### 2.7 Antitumor Immune Responses (DCs, CD4 T, CD8 T, NK, and M1-Macrophage) Induced by NIR-III/-IV PDT

Antigen presenting cells (APCs) play a pivotal role in stimulating and regulating adaptive immunity. Dendritic cells (DCs) are one of the major APCs involved in both the initiation and activation of adaptive immunities. DCs are the key antigen-presenting cells responsible for taking up, processing, displaying, and delivering tumor-associated neoantigens to lymph nodes, contributing to the initiation of specific antitumor immune responses.^46^ In the in vivo DC maturation experiments, mice were randomly divided into 15 groups. The maturation level of DCs was accessed by measuring the expression levels of costimulatory molecules CD11c specific DCs with the maturation markers CD80 and CD86.^47^ Mice from different groups were intravenously injected with LaB_6_–PEG–folate NPs (50 mg/kg) and intraperitoneal injected with anti-OX40 (50 μg/mouse) to evaluate the in vivo DC maturation level. On day 14 after the treatment, the mice were sacrificed; and tumor draining lymph nodes were collected, assessed using flow cytometry, and shown in Figure 4e,f. The “LaB_6_–PEG–folate NPs + laser irradiation + anti-OX40”-treated groups showed higher levels of DC maturation compared to those non-photo-irradiation groups. We found the percentage of DC maturation levels for LaB_6_–PEG–folate NPs + laser irradiation + anti-OX40 treated groups are 3.6-fold (808 nm), 4.2-fold (1064 nm), 5.6-fold (1550 nm), and 6.5-fold (2240 nm) increase, respectively, for CD80, and 2.3-fold (808 nm), 2.3-fold (1064 nm), 4.2-fold (1550 nm), and 5.5-fold (2240 nm) increases, respectively, for CD86 compared to the dark control group (1.0-fold). The corresponding flow cytometry analyses are shown in Figures S20 and S21. When compared to other non-photoirradiation groups, LaB_6_–PEG–folateNPs + laser irradiation + anti-OX40-treated groups could effectively promote DC maturation, which is vital for the priming and expansion of tumor-recognizing new cytolytic T cells. The in situ generation of large amounts of tumor-associated neoantigens by phototherapies plays a crucial role in promoting the maturation levels of DCs in the following sequence: 4.7-fold (808 nm), 5.9-fold (1064 nm), 7.6-fold (1550 nm), 8.3-fold (2240 nm) increases, respectively, for CD80 and 4.6-fold (808 nm), 5.9-fold (1064 nm), 7.5-fold (1550 nm), 7.3-fold (2240 nm) increases, respectively, for CD86 as compared to the dark control group (1.0-fold). Notice that the immunotherapy reagent anti-OX40 alone-treated group has a very modest effect on promoting maturation of DCs (1.6-fold as compared to the dark control group).

To further investigate the underlying mechanism responsible for the boosted immunotherapy efficacy in the suppression of the remote tumor growths, the infiltration levels of cytotoxic T lymphocytes (CD8^+^ T cells) and helper T lymphocytes (CD4^+^ T cells) at the remote tumor sites were measured using flow cytometry.^1,48^ Cytotoxic CD8^+^ T lymphocytes could directly kill cancer cells, whereas helper T cells play an important role in the activation of adaptive antitumor immunities.^49^ As depicted in Figure 4g, all LaB_6_–PEG–folate NPs + photoirradiation-treated groups showed enhanced levels of CD4^+^ T cells at the remote tumor sites in the following sequence: 7.0-fold (2240 nm) > 6.2-fold (1550 nm) > 4.8-fold (1064 nm) > 3.8-fold (808 nm) > dark control (1.0-fold). Notice that the immunotherapy reagent anti-OX40 alone-treated group has only a medium increase level (2.6-fold) of the CD4^+^ T cells at the remote tumor sites. Upon the combination of the phototherapies with anti-OX40, the relative proportion of the CD4^+^ T cells at the remote tumor sites were dramatically boosted in the following sequence: 15.4-fold (2240 nm) > 14.3-fold (1550 nm) > 9.0-fold (1064 nm) > 7.8-fold (808 nm) > dark control (1.0-fold). Similar results were observed in the levels of CD8^+^CD45^+^ T cells at remote tumor sites (Figure 4h). Upon the combination of phototherapies with anti-OX40, the frequencies of the CD8^+^CD45^+^ T cells were drastically boosted in the following sequence: 21.6-fold (2240 nm) > 18.1-fold (1550 nm) > 15.9-fold (1064 nm) > 14.3-fold (808 nm) ≫ 3.7-fold (anti-OX40 alone) > dark control (1.0-fold). These results indicated that the presence of additional in situ-generated whole cancer cell vaccines (generated from phototherapies) drastically boosts the efficacy of anti-OX40-based immunotherapy at the remote tumor sites. To enhance clarity and reliability, we now present both fold change (Figure S22) and percentage values for the relevant immune cell populations.

Natural killer (NK) cells are unique lymphocyte subpopulations and contain unique cytoplasmic granules. NK cells play a pivotal role in the anticancer immunity.^47^ Mature NK cells are identified by its characteristic CD3, NK1.1, CD49b, and NKG2D transmembrane receptor proteins expressed on their membrane surface.^50^ As shown in Figure 4i, phototherapy-treated groups all have high infiltration levels of CD3^+^NK1.1^+^ NKT cells at the remote tumor sites in the following sequence: 5.9-fold (2240 nm) > 5.7-fold (1550 nm) > 4.8-fold (1064 nm) > 4.3-fold (808 nm) > 1.0-fold (dark control). Notice that the anti-OX40 alone-treated group also has a small increase in the infiltration level of 1.9-fold compared to the dark control group. Upon combination of anti-OX40 and phototherapy, the NKT cell infiltration levels at the remote tumor sites were largely enhanced in the following sequence: 10.5-fold (2240 nm) > 9.9-fold (1550 nm) > 8.1-fold (1064 nm) > 4.9-fold (808 nm) > 1.9-fold (anti-OX40 alone). Analysis of mature NK cells in spleen (Supporting Information, Figures S23 and S24) was also conducted, and a similar increase trend was observed. The antitumor function of NK cells is enhanced by cytokines, such as IL-2 secreted by activated DCs, and by IFN-α/β produced by other innate immune cells. Activated NK cells could secrete large amounts of antitumor cytokines, such as IFN-γ and TNF-α. The results in Figure 4i clearly show that the presence of in situ-generated whole cancer cell vaccine (by phototherapies) could effectively boost the efficacy of anti-OX40-based immunotherapy against remote tumor growth via promoting the maturation and infiltration of NK cells into the remote tumor sites.

### 2.8 Responses of Immunosuppressive Immune Cells and Cytokines (M2-Macrophages, Treg Cells, IL-10, and TGF-β) Induced by NIR-III/-IV PDT

Tumor-associated macrophages (TAMs) account for a substantial fraction of tumor-infiltrating immune cells.^51^ Tumor-associated macrophages (TAMs) are classified into different functional phenotypes, denoted classically activated macrophages (i.e., the M1-phenotype) and alternatively activated macrophages (i.e., the M2-phenotype). M1-TAMs play a critical role in the presentation of tumor-associated neoantigens; conversely, M2-phenotype TAMs exert pro-tumorigenic activities.^52,53^ Thus, targeting M2-TAMs could be important to alter the immune-suppressive tumor microenvironment (TME). We observed a large increase of M1-TAMs (F4/80^+^ and CD86^+^), but a slight decrease of M2-TAMs (F4/80^+^ and CD206^+^) for all the “LaB_6_–PEG–folate NPs + laser irradiation” groups at remote tumor sites (Figure 4j,k).^54^ Such a trend in the increase of antitumor M1-macrophages, but decreases in the tumor-protecting M2-macrophages for the “LaB_6_–PEG–folate NPs + laser irradiation” groups were dramatically enhanced by the presence of anti-OX40. Such a result is consistent with the earlier conclusion that the presence of in situ generation of whole cancer cell vaccines (via LaB_6_ NPs-mediated phototherapies) could drastically enhance the anti-OX40-based immunotherapy efficacy in suppressing the growth of remote tumors where no phototherapies were exerted. Again, notice that the immunotherapy reagent anti-OX40 alone has a quite modest effect on the regulation of the expression levels of both M1- and M2-macrophages (Figures 4j/S25 and 4k/S26).

Regulatory T cells (*Tregs*) are critical in maintaining immune tolerance and suppressing autoimmunity. The transcription factor Foxp3 serves as the main switch controlling the development and function of Treg.^55^ Representative flow cytometry analysis data of CD4^+^Foxp3^+^ Treg cells are shown in Figure S27. The relative proportion of Treg cells (CD4^+^CD25^+^Foxp3^+^) in the anti-OX40 alone-treated group in lymph nodes is lower than that of the dark control group. Binding of anti-OX40 onto the OX40 receptor on Treg cells is known to suppress the activity of immune-suppressive Treg cells.^56^ The data in Figure 4l showed that in the absence of anti-OX40, the infiltration levels of Treg cells for the phototherapy groups are lower than that for the anti-OX40 alone-treated group, indicating that the whole cancer cell vaccines generated by all NIR-I PTT and NIR-II/-III/-IV PDT therapies could effectively stimulate the host antitumor immunity, suppress the activity of Treg cells, as well as boost the anti-OX40-based immunotherapy efficacy in suppressing the activity of the immunosuppressive Treg cells in lymph nodes.

In the literature, it is known that IL-10 and TGF-β are two major immunosuppressive cytokines secreted by Treg, M2-macrophage, and MDSCs to inhibit the activities of antitumor immune cells, including T cells, NK cells, and DCs.^57,58^ We, therefore, also measured the expression levels of IL-10 and TGF-β after phototherapy treatments in the blood serum. As shown in Figure 5, the expression levels of both IL-10 and TGF-β cytokines were effectively suppressed by different phototherapies, but very modestly suppressed by immunotherapy reagent anti-OX40 alone. The effect of anti-OX40 on suppressing the expression levels of immunosuppressive cytokines IL-10 and TGF-β was, however, dramatically boosted in the presence of in situ-generated whole cancer vaccines (generated by phototherapies). Such a result is consistent with the above observation on the suppression of the expression levels of immunosuppressive Treg and M2-macrophages shown in Figure 4k,l.

**Figure 5:**
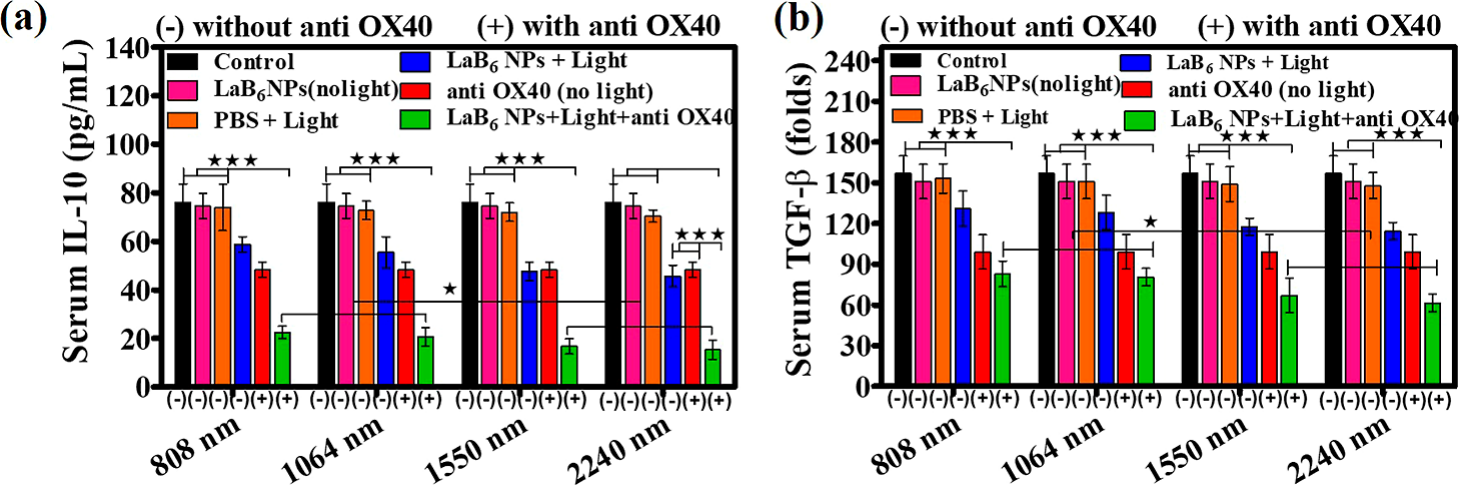
The expression levels of immunosuppressive cytokines: (a) IL-10 and (b) TGF-β in the blood serum of different treatment groups. (*n* = 2 mice per group). Statistical significance was determined using one-way ANOVA followed by the Tukey’s posthoc test. *P < 0.05, **P < 0.01, ***P < 0.001, comparing treatment groups to the control and among selected group pairs as indicated.

Overall, in situ-generated whole cancer cell vaccines could dramatically boost the efficacy of anti-OX40-based immunotherapy in suppressing the growths of remote tumors via stimulated maturation of DCs in nearby lymph nodes, elevated generation, and tumor infiltration of CD4^+^ T cells, newly generated tumor-recognizing cytotoxic CD8^+^ T cells, NK cells, M1-macrophages at remote tumor sites, as well as the suppression of the activities of M2-macrophages (at remote tumor sites) and Treg cells (in lymph nodes), in company with elevated expression levels of antitumor cytokines (including TNF-α, IFN-γ, IL-2, and IL-12), as well as suppression of immunosuppressive cytokines (including IL-10 and TGF-β) in the blood serum.

### 2.9 Short- and Midterm Cytotoxicities of LaB_6_–PEG–Folate NPs

The in vivo short- and midterm cytotoxicities of LaB_6_–PEG–folate NPs in healthy mice were evaluated using the blood biochemistry test. The levels of various parameters related to hepatic (AST, ALT, ALP, and ALB) and nephritic functions (BUN and CRE) were evaluated on days 1, 10, and 20 for mice receiving intravenous injection of LaB_6_–PEG–folate NPs (at a dose of 50 mg/kg) and using an FDA-approved chemodrug, DOX, for comparison. As shown in Figure S28, although nephritic functions were slightly higher for LaB_6_–PEG–folate NPs-treated groups as compared to the control group during the 21 day observation period. However, all these blood chemistry levels were lower than those of the FDA-approved, a clinically used chemodrug, DOX,-treated group. The results clearly showed that LaB_6_–PEG–folate NPs are biocompatible and safe to use without any noticeable short- and midterm cytotoxicities to both the liver and kidney.

The optical images of the mice receiving various treatment conditions are shown in Figure S29, which clearly depict the suppression of both primary and the remote tumors in the groups treated with LaB_6_–PEG–folate NPs + NIR light + anti-OX40 on day 16 in comparison with the other groups. Histopathological examination (H&E) and caspase-3 staining experiments were performed for both the primary and the remote tumors for different treatment groups. Histopathological analysis was performed on both primary and remote tumors from the different treatment groups using hematoxylin and eosin (H&E) staining and caspase-3 immunostaining. Tumor sections were collected and stained with H&E to evaluate the overall tissue morphology and identify features, such as necrosis, as shown in Figure S30 (primary tumors) and S31 (remote tumors). The group treated with LaB_6_–PEG–folate NPs and laser irradiation and anti-OX40 showed substantial cancer cell destruction in both primary and remote tumors. In these H&E images, necrotic regions are indicated with arrows for clarity. Caspase-3 immunostaining was conducted in parallel to assess apoptosis in the same tumor tissues, with results shown in Figure S32 (primary) and S33 (remote). Apoptotic cells showing positive caspase-3 staining are also marked with arrows to guide interpretation.

A higher number of tumor necrosis and apoptosis was observed for both the primary and the remote tumors in the “LaB_6_–PEG–folate NPs + laser irradiation + anti-OX40”-treated groups in comparison with the remaining groups, showing noticeable cancer cell deaths. Further, after phototherapy, major organs including the spleen, kidney, heart, and liver were collected to determine the apoptotic cell deaths using H&E staining. Notice that these major organs were not exposed to the laser light irradiation during the phototherapy treatments. As shown in Figures S34–S37, neither organ damage nor any pathological changes were detected, indicating that the LaB_6_ NPs exhibit high safety and low dark cytotoxicity. In general, the improved inhibition of primary and remote tumors with a remarkable survival rate indicates the application prospects of LaB_6_–PEG–folate NPs-based NIR light phototherapies for reversing the poor effectiveness of anti-OX40-based immunotherapy.

### 2.10 Measurements of the *K*_i_-67 Expression Level and the IFN-γ ELISPOT Assay

*K*_i_-67 is a nuclear protein and a reliable marker for the evaluation of T-cell proliferation.^59,60^ The higher level of the *K*_i_-67 protein represents the higher level of T-cell proliferation. We first examined the proliferation of cytotoxic T cells in various treatment groups focusing on CD45^+^CD8^+^ *K*_i_-67^+^ cells in the spleens of B16BL6 tumor-bearing mice using flow cytometry. As shown in Figure S38 (also shown below), the mice group treated with combined “NIR-IV PDT + anti-OX40 immunotherapy” exhibited a significantly higher elevated frequency of proliferating CD8^+^ T cells (17.6%), as compared to other treatment groups, such as LaB_6_–PEG–folate + 2240 nm (13.5%), anti-OX40 + 2240 nm (10.2%), PBS + 2240 nm (9.35%), LaB_6_–PEG–folate alone in the dark (8.62%), anti-OX40 alone in the dark (9.76%), and the untreated control group (8.14%). Such a result clearly indicates that a combination of NIR-IV PDT and anti-OX40 immunotherapy could result in the largest T-cell proliferation. The elevated expression of *K*_i_-67 within the CD8^+^ T-cell population indicates effective T-cell priming and a strong proliferative immune response induced by the combined “NIR-IV PDT and anti-OX40 immunotherapy” treatment.

Interferon γ (IFN-γ) is a key cytokine that plays a significant role in T-cell activation and function. The higher level of the IFN-γ cytokine represents the higher level of T-cell activation.^61,62^ To evaluate and compare the extents of T-cell activation by different treatment groups, we performed an IFN-γ ELISPOT assay on splenocytes collected from various treatment groups at 12 days post-treatment. Mice receiving the combined “NIR-IV PDT (i.e., LaB_6_–PEG–folate + 2240 nm irradiation) and anti-OX40 immunotherapy” treatment showed a significantly higher number of IFN-γ-producing cells (103 ± 10), as compared to other treatment groups, such as the “LaB_6_–PEG–folate + 2240 nm” treatment group (72 ± 6), the “anti-OX40 + 2240 nm” treatment group (30 ± 5), the “PBS + 2240 nm” treatment group (23 ± 4), the “LaB_6_–PEG–folate in dark” treatment group (9 ± 4), the “anti-OX40 in dark” treatment group (21 ± 4), and the untreated control group (4 ± 2) (see Supporting Information, Figure S39). The IFN-γ ELISPOT assay data clearly indicate that the combined “NIR-IV PDT + anti-OX40 immunotherapy”-treated group has the highest level of T-cell activation among all of the treatment groups.

Taken together, both the *K*_i_-67 assay and the IFN-γ ELISPOT assay data demonstrate that the combined “NIR-IV PDT + anti-OX40 immunotherapy” treatment can best promote both the CD8^+^ T cells’ proliferation and activation, providing compelling evidence of a robust systemic antitumor immune response and affirming the superiority of this combined therapeutic approach in eliciting durable and effective antitumor immunity.

## 3 Discussion

### 3.1 A Newly Proposed “Blind T Cells” Model to Rationalize the Ineffectiveness of Immunotherapies

Under normal conditions, the natural deaths of cancer cells are very little, which generates very limited amounts of tumor-associated neoantigens for priming, proliferation, and expansion of newly generated tumor-recognizing T cells. In other words, under normal conditions, the fraction (or quantity) of tumor-recognizing T cells is very small. The large majority of T cells are actually “blind” and do not recognize tumor cells (see Scheme 1). Conventional immunotherapy, such as checkpoint blockade inhibitors, anti-PD-1/-PD-L1, can block the pathways used by cancer cells to trick those very small fraction of tumor-recognizing cytotoxic T cells, resulting in the very modest improvement of antitumor immunity and serious autoimmune adverse problems from those large majority of “blind” T cells. This can explain why the immune checkpoint blockade inhibitor-based immunotherapy does not work well for most of cancers but only work well for those cancer patients with high DNA mutation rates, such as melanoma, renal cell carcinoma, and nonsmall cell lung cancer, where high DNA mutation rates leads to the generation of more tumor-specific neoantigens,^63,64^ and thus priming of higher fraction of tumor-recognizing T cells. By the in situ generation of large amounts of tumor-associated neoantigens, especially via NIR-III/-IV PDT, to initiate the priming, proliferation, and expansion of more tumor-recognizing newly generated T cells in the co-presence of signals I, II, and III (see Scheme 1), large amounts of tumor-recognizing cytotoxic T cells will be produced. Under such a condition, the immunotherapy reagents can remove the “brake” of these tumor-recognizing newly generated cytotoxic T cells, leading to effective antitumor actions and thus boosted immunotherapy efficacy. Only in the presence of large amounts of tumor-recognizing T cells, the conventional checkpoint blockade inhibitor immunotherapies will work well. Besides the modality of in situ generation of whole cancer cell vaccines presented in this work, alternatively, one can take out those naive T cells from a cancer patient, re-engineer those T cells ex vivo with a few (1 or 2) chimeric antigen receptors (CAR) to generate CAR-T cells that recognize a few features of tumor cells, and re-infuse those ex vivo prepared CAR-T cells back to a cancer patient’s body so that these re-infused CAR-T cells can recognize and destroy cancer cells. It was recently demonstrated that the CAR-T therapy works very well in several clinical trials for the complete eradication of advanced leukemias and lymphomas.^65,66^ Recently, it was also reported that the administration of cancer mRNA can largely enhance the antitumor immunity via the generation of large amounts of tumor-specific proteins/neoantigens in situ, which subsequently induces the generation of a large amount of tumor-recognizing cytolytic T cells. A combination of mRNA cancer vaccine with immune checkpoint blockade immunotherapy can effectively cure pancreatic cancer.^67^ These observations highlight two important issues, i.e., (1) activation of existing (old) T cells is not enough to suppress tumor growths if without producing a large quantity of tumor-recognizing T cells since most old existing T cells are actually “blind”, and do not recognize the features of tumor cells. (2) Generation of large amounts of tumor-specific neoantigens is paramount (or mandatory) for the activation of host anticancer immunity via priming and expansion of new cytotoxic tumor-recognizing T cells in the presence of costimulatory signals II and III. By using the tumor-associated neoantigens as the checkpoints, cancer cells can no longer stealth and evade from attacks by those newly generated tumor-recognizing T cells.

**Scheme 1:**
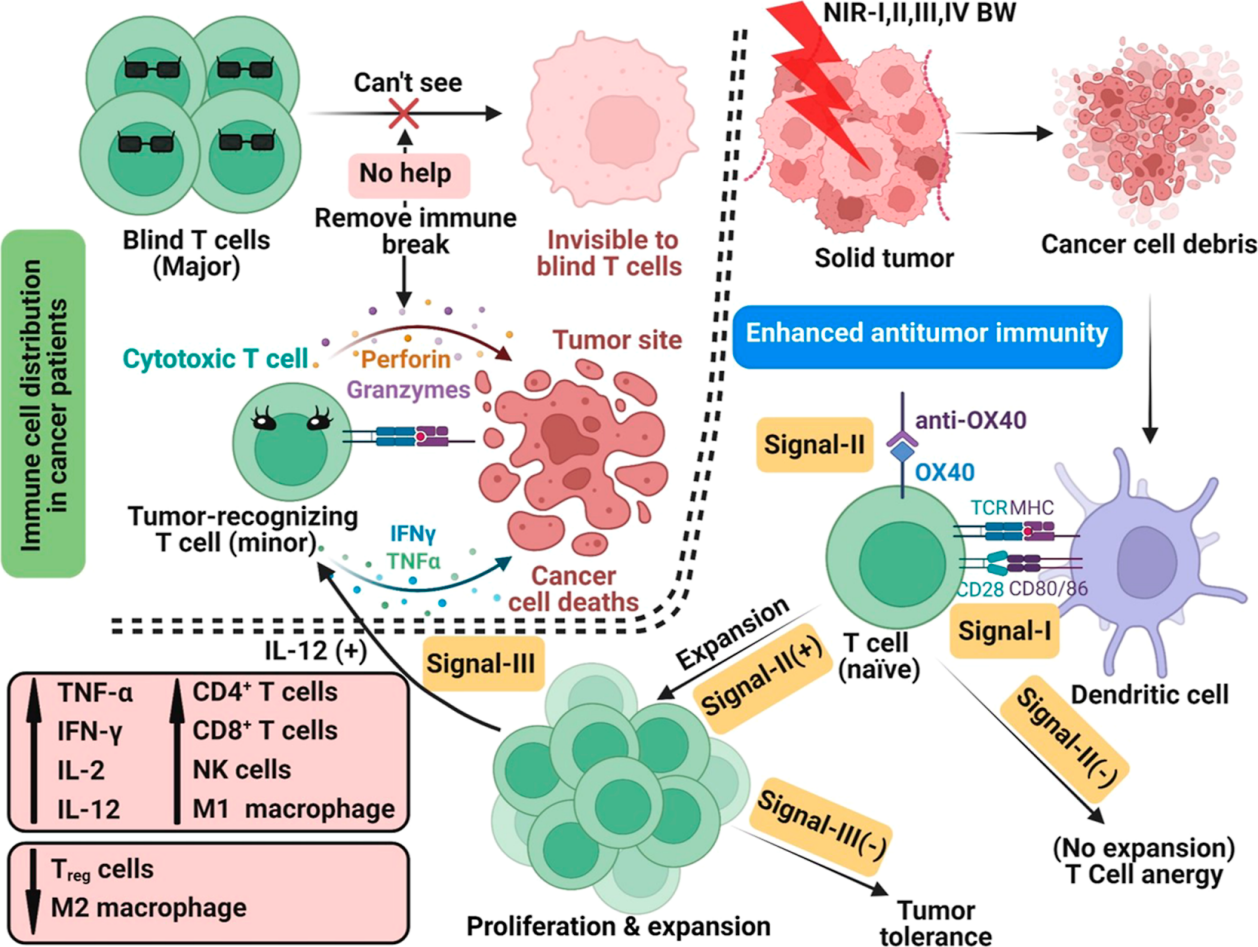
A “Blind T Cells” Model to Rationalize the Ineffectiveness of Immunotherapies, and a Strategy to Reverse Ineffectiveness of Immunotherapies via Combination of the In Situ Generated Whole Cancer Cell Vaccine by NIR-I/-II/-III/-IV PDT with Immunomodulator Anti-OX40^a^

a Under normal conditions, large majority of existing CD8^+^ T cells in cancer patients are actually “blind” and cannot recognize cancer cells. Removal of immune break does not help these blind T cells to recognize and kill cancer cells. The signals I, II, and III are mandatory for the priming, proliferation, expansion and development of cytolytic functions of tumor-recognizing cytolytic new CD8^+^ T cells. Generation of large quantity of tumor-recognizing new cytotoxic T cells provides a solution to reverse an otherwise ineffective immunotherapy to become effective.

To effectively generate newly produced tumor-recognizing cytolytic CD8^+^ T cells, three criteria must be fulfilled, namely, (a) supplying large amounts of tumor-associated neoantigens, (b) priming and expansion of T cells in the copresence of signal II (i.e., costimulation by anti-OX40 or anti-CTLA 4), and (c) costimulation of signal III, such as IL-12. In the literature, several reports have demonstrated that good to excellent tumor suppression effects could be observed when combining in situ generation of cancer cell vaccines (using radiotherapy, photothermal therapy, chemotherapy, etc.) with checkpoint blockade immunotherapeutic reagents (such as anti-PD-1/anti-CTLA-4).^68–71^ However, it was never explicitly pointed out in these prior literature reports regarding the origin of ineffective immunotherapies as well as the concept of increasing tumor-recognizing new cytolytic T cells for reversing the ineffectiveness of immunotherapies. The strategy presented in the current work for the reversing of ineffective immunotherapies is feasible and cost-effective compared with both CAR-T and mRNA cancer vaccine methods because it is process-wise simple, far more cost-effective, recognizes multiple tumor-specific features, and does not require cumbersome personalized cancer genome sequencing analysis and the synthesis of limited numbers of tumor-specific neoantigens, genetic modification of T cells to allow the generation of limited numbers of chimeric antigen receptor(s), chemical modification of mRNAs, and so on.

Currently, all the literature reported papers regarding phototherapy + immunotherapy are located in the NIR-I, II, and III biological windows.^12,72^ No any paper is within the NIR-IV (2100–2300 nm) biological window.^73^ Most literature reported a combination of NIR-I/-II PDT/PTT and immunotherapy can only destroy primary tumors but are not effective to eradicate metastatic tumors with only very few exceptions,^74^ due to the poor cancer cell-killing efficacies of visible light or NIR-I/-II PTT/PDT, inefficient generation of tumor-associated antigens for the activation of host antitumor immunity as well as a lack of the co-stimulation of the signal II required for the proliferation and expansion of T cells.^33,34^ Photothermal therapy-induced cancer cell deaths undergo a combination of apoptosis and necrosis pathways (with nearly equal fractions), whereas the photodynamic therapy could generate more necrotic cancer cell deaths than apoptotic cell deaths. Necrotic cancer cells could produce more cellular debris as compared to apoptotic cells, leading to a better stimulation of antitumor immune responses. It was also reported that the necrotic cellular debris from necrotic dead cancer cells could induce immunogenic responses, including the maturation of DCs and subsequent T-cell priming, but not those from apoptotic dead cells.^75^ It is necessary to improve the PDT-induced cancer cell killing efficacies by using NIR-III or NIR-IV PDT, of which the NIR excitation light has much deeper tissue penetration depths. Upon neoantigen presentation by matured DCs (i.e., the signal I), co-stimulation of CD4^+^ and CD8^+^T cells by anti-OX40 (i.e., the signal II) will stimulate T cells to release antitumor cytokines (including IL-4, IL-6, IL-12, TGF-β, etc.) to serve as the signal III and promote development of cytolytic effector functions of CD8^+^ T cells.^76^ Within the immunosuppressive TME, stimulation of CD8^+^ T cells by the signal III (or IL-12) will also promote the T cells to release various cytokines, including perforins and Granzymes, to destroy tumor cells.^77^ While binding of anti-OX40 agonist to the OX40 receptor on Treg cells can inhibit their ability to suppress the cytotoxic functions of CD8^+^ T cells via turning off the Foxp3^+^ signal pathway.^78^ The administration of the anti-OX40 agonist was also found to promote infiltration of CD8^+^ T cells and decrease in the amounts of immunosuppressive macrophages, MDSCs, and Treg in the tumor site.^79^ Overall, anti-OX40 is able to activate CD4^+^ T cells, CD8^+^ T cells, as well as suppresses the activities of Treg cells and M2-macrophages simultaneously.^56^ Among various immunotherapy agents, preclinical studies suggest that anti-OX40 may offer a broader immunomodulatory profile compared to anti-PD-L1 or anti-CTLA-4, by not only suppressing Treg and M2 macrophages but also promoting CD8^+^ T-cell activation, improving T-cell priming, and enhancing T-cell infiltration into tumors.^80–82^ However, these findings remain to be validated in clinical settings. We have clearly specified that these observations are based on preclinical (murine) models. Activation of CD8^+^ T cells by co-stimulator anti-OX40 alone does not work well, due to the lack of sufficient amounts of tumor-associated neoantigens as well as the lack of signal III (i.e., IL-12) required for the expansion and the development of cytolytic functions of new tumor-recognizing T cells, as evidenced by the very minor effect on the suppression of tumor volumes (see, Figure 2e) and in the improvement of the average survival rate (see, Figure 2g) as demonstrated in this study.

The importance of tumor recognition by cytotoxic CD8^+^ T cells was not fully realized in prior studies.^3–8^ To rationalize the ineffectiveness of immunotherapies, several hypothesis have been reported, including T-cell exhaustion in the tumor microenvironment,^3,4^ immunosuppressive tumor microenvironment (TME),^7,8^ and lack of neoantigens.^5,6^ Exhausted CD8^+^ T cells are featured by the upregulated expression of inhibitory receptors (such as PD-1, CTLA-4, TCR, and TIGIT) as well as the decreased production of pro-inflammatory cytokines (such as IL-2, TNF-γ, and IFN-γ). Immunosuppressive TME is featured by the large amounts of immunosuppressive cytokines (such as PGE2, TGF-b, IL-10, etc.) secreted by cancer cells, cancer stem cells, Treg, MDSCs, and M2-macrophages. These two hypotheses are based on an inexplicitly written assumption that all antigen-specific CD8^+^ T cells can recognize cancer cells, but become malfunctioned in the local TME. The third “lack of neoantigens” hypothesis points out a factor indirectly contributing to the failure of most immunotherapies but did not identify the key principal factor directly responsible for the ineffectiveness of most immunotherapies.

## 4 Conclusion

In summary, we have explicitly pointed out that the key principal factor directly responsible for the failure of most immunotherapies is the lack of tumor-recognizing by existing CD8^+^ T cells. We proposed a “Blind T Cell” model to rationalize the ineffectiveness of immunotherapies. The “Blind T Cells” model uncovers the incorrect assumption in the oncology-immunotherapy field that existing antigen-specific CD8^+^ T cells can recognize the features of cancer cells. Most immunotherapies only increase the activities of those large majority of existing “blind” T cells that do not recognize cancer cells, but did not increase the amount (or fraction) of the tumor-recognizing cytolytic T cells. We also demonstrated a strategy to reverse an otherwise ineffective (or weakly effective) anti-OX40-based immunotherapy to become very effective by increasing the quantity of new tumor-recognizing cytolytic CD8^+^ T cells before the administration of immunotherapy reagents. We illustrated that by combining immuno-modulator anti-OX40 and in situ generated whole cancer cell vaccine, mediated by advanced NIR-III/-IV PDT, the proportions of various antitumor-related immune cells, including DCs, CD4^+^ T cells, CD8^+^ T cells, M1-macrophage, and NK cells, all were largely increased at remote tumor sites and in peripheral blood accompanied by elevated levels of various antitumor-related cytokines, including IL-2, IL-12, TNF-α, and INF-γ. Meanwhile, the proportions of immunosuppressive cells, such as Treg, M2-macrophage as well as immunosuppressive cytokines, such as IL-10 and TGF-β, are downregulated. The unprecedented NIR-IV PDT not only could in situ generate large amounts of whole tumor cell vaccines required for priming of new tumor recognizing cytolytic CD8^+^ T cells but also could induce inflammatory responses for the generation of cytokine, IL-12. A large increase in the population and activation of tumor-recognizing new cytolytic T cells, as evidenced by the *K*_i_-67 measurement and the IFN-γ ELISPOT assay, by the combined NIR photodynamic-anti-OX40 immunotherapy, is the key criteria to conquer the ineffectiveness of immunotherapies and reverse the otherwise ineffective anti-OX40-based immunotherapy to become effective, resulting in the effective suppression of remote tumor growths observed in this study.

The average half-life spans for mice groups treated with LaB_6_–PEG–folate NPs plus 1550 nm/2240 nm NIR light irradiation and the presence of the anti-OX40 immunomodulator were significantly extended to 83 days (2240 nm) and 76 days (1550 nm), respectively, which are far longer than the anti-OX40 alone-treated group (21 days) and the PBS control group (15 days). The administration of immunotherapeutic reagent anti-OX40 alone without providing additional cancer neoantigens only leads quite poor antitumor effects and short average lifespans of mice bearing B16BL6 melanoma cancer. Overall, the key issue to overcome the ineffectiveness of immunotherapies is to increase the amount of tumor-recognizing new cytolytic T cells, followed by removing the antitumor immune “brake” of these newly generated tumor-recognizing cytolytic T cells via the administration of various kinds of immune co-stimulators/activators/checkpoint blockade inhibitors. We believe that such a strategy is broadly applicable to render otherwise ineffective immunotherapies become very effective. The current work also provides a guideline for the clinical treatment of patients using immunotherapies, namely, immunotherapies should be performed immediately after the use of cancer cell killing methods (such as photodynamic/photothermal/particle/chemotherapy/radiotherapy, etc.) to induce the in situ generation of large amounts of new tumor-recognizing cytotoxic T cells.

## 5 Methods

### 5.1 Synthesis of LaB_6_ NPs

Briefly, lanthanum chloride (392 mg), sodium borohydride (363 mg), 0.5000 g of NaCl, and 0.5000 g of KCl were well hand mixed for half an hour and subsequently placed under an argon atmosphere in a tube furnace at 450 °C for 7 h at a temperature rising rate of 10 °/C. The final product was allowed to cool at room temperature, followed by repeated washing at 10,000 rpm for 10 min with methanol and HCl and finally washed with D.I. water to remove the impurities to obtain the final product. The as-synthesized LaB_6_ NPs were functionalized with citric acid, followed by further coupling with the NH_2_–PEG–folate moiety by 1-ethyl-3-(3-dimethylaminopropyl) carbodiimide (EDC) coupling to form LaB_6_–PEG–folate NPs to ensure the targeting ability of the folate moieties to the folate receptors on cancer cells.^2^

### 5.2 The Molar Extinction Coefficients of LaB_6_ NPs

The molar extinction coefficient of LaB_6_ NPs was measured by following a literature procedure.^27^ In brief, the average size of the LaB_6_ NPs was determined from DLS measurements. From the average particle size, one can calculate the volume for a nanoparticle. Then, the number of LaB_6_ molecules in a nanoparticle can be calculated by multiplying the density of LaB_6_ NPs (obtained from the literature) with the volume for a particle, followed by division by the molecular weight of a LaB_6_ molecule. From the ICP–MS measurement of a LaB_6_ NPs-containing aqueous solution, one can obtain the total weight (and thus the total number) of LaB_6_ NPs in the aqueous solution. Then, the number of LaB_6_ NPs in the solution can be calculated by dividing the total number of LaB6 molecules by the number of LaB_6_ molecules in a particle. Dividing the as-obtained LaB_6_ NPs in the solution by the solution volume one obtains the concentration of LaB_6_ NPs in the solution. From the Beer–Lambert law, ε = *A*/*bc*, where *A* = absorbance, *b* = 1 cm, and *c* = concentration of LaB_6_ NPs, one further calculates the extinction coefficients of LaB_6_ NPs at a particular wavelength.

### 5.3 EPR Measurements

Electron paramagnetic resonance (EPR) measurements (Bruker, Elexsys series, E-580 with X-band: CW/FT) were performed to detect the generation of ROS species, including both singlet oxygen and hydroxyl radicals. A 1.0 M 2,2,6,6-tetramethylpiperidine (TEMP) probe in D_2_O was mixed with 50 μL of LaB_6_ NPs (1 mg/mL) and then irradiated with 808, 1064, 1550, or 2240 nm NIR light with the same power intensity of 300 mW/cm^2^ for 5 min for all wavelengths. The final solution was then analyzed in EPR. Twenty mM 5,5-dimethyl-1-pyrroline *N*-oxide (DMPO) was used for the detection of the hydroxyl radical (^•^OH). LaB_6_ NPs (1 mg/mL) was dispersed in an aqueous solution, followed by photoirradiation. The 2240 nm diode laser was constructed by Tan-Yu Technology Co., LTD (Kaohsiung, Taiwan) using a 2240 nm laser diode (model m2k-BA-2240-0500-CM, Coherent, DILAS, Germany).

### 5.4 Measurement of H_2_O_2_ Using an Amplex Red Assay

An Amplex red (from Invitrogen, USA) assay was used to detect the formation of H_2_O_2_ from the photoirradiation of LaB_6_–PEG–folate NPs (0, 10,50 and 100 μg/mL) upon NIR light irradiation. The standard curve for H_2_O_2_ was obtained using different concentrations of H_2_O_2_ (0, 1, 2, 4, 10, and 20 μL) from a stock H_2_O_2_ (3%) solution. LaB_6_–PEG–folate NPs (0, 10, 25, 50, and 100 μg/mL) were then irradiated with 808 nm (300 mW/cm^2^; 15 min), 1064 nm (300 mW/cm^2^; 12 min), 1550 nm (300 mW/cm^2^; 12 min), and 2240 nm (300 mW/cm^2^; 12 min), respectively, in a 96 well culture plate. Then, the solution was centrifuged to remove LaB_6_–PEG–folate NPs, and 50 μL of the supernatant was treated with 50 μL of the assay reagents, followed by a 30 min incubation at room temperature. The fluorescence was monitored using the microplate reader with an excitation wavelength of 540 ± 10 nm and fluorescence emission detection at 590 ± 10 nm.

### 5.5 Quantification of Cell Viabilities Using the MTT Assay

2.0 × 10^4^ cells/mL of B16BL6 cells were cultured on 24-well plates. After 24 h incubation, LaB_6_–PEG–folate NPs were added to each plate and incubated in the dark for 24 h at 37 °C. Phototoxicities of LaB_6_–PEG–folate NPs upon irradiation with 808 nm (300 mW/cm^2^; 15 min), 1064 nm (300 mW/cm^2^; 12 min), 1550 (300 mW/cm^2^; 12 min), and 2240 nm (300 mW/cm^2^; 12 min) NIR light under the same experimental conditions. Subsequently, after photoirradiation, the cells were incubated for 12 h and a MTT (0.5 mg/mL) reagent was then added to the cells and incubated for another 4 h. Then, the upper layer solution was removed and 1 mL of DMSO was added. The final solution was centrifuged at 13,000 rpm, and the upper solution was subjected to ELISA measurements at an optical absorbance of 570 nm wavelength. The values of the optical absorbance were finally converted to cell viabilities based on the standard curve from the control experiments that were performed under the same experimental conditions. The cell viability study using a healthy human HUVEC cell line was conducted in a similar way.

### 5.6 Heat Shock Protein Analysis

2 × 10^5^ cells/mL of B16BL6 melanoma cancer cells were cultured on a 12-well plate and incubated for 24 h. Then, LaB_6_–PEG–folate NPs were added to the cells and incubated for 12 h. Subsequently, cells were subjected to photoirradiation with different light sources. After irradiation, cells were stained with an Alexa Fluor 640-conjugated HSP 70 antibody (1:50 dilutions; Cell Signaling, USA), followed by flow cytometry analysis.

### 5.7 DPBF Experiments

1 mg/mL portion of LaB_6_ NPs and 0.08 mM of 1,3-diphenyl isobenzofuran (DPBF) were mixed and irradiated with 808, 1064, 1550, and 2240 nm diode laser (300 mW/cm^2^ 12 min), respectively. The optical absorbance of the solution was measured at 410 nm with a time interval of 2 min using a UV–visible NIR spectrometer.

### 5.8 SOSG Measurements

1 mg/mL of LaB_6_–PEG–folate NPs were mixed with 20 μL of SOSG (Invitrogen, USA) in D_2_O, followed by photoirradiation using 808 nm, 1064 nm, 1550 nm, and 2240 nm (300 mW/cm^2^, 5 min) diode lasers, respectively. The solution was then analyzed using a fluorescence emission spectroscope using an excitation wavelength of 504 nm.

### 5.9 Apoptosis Assay

2 × 10^5^ cells/well of B16L6 melanoma cancer cells were cultured in a 6-well plate for 24 h. Then, LaB_6_–PEG–folate NPs stock solutions were added and further incubated for 12 h. The cells were photoirradiated with 808 nm (300 mW/cm^2^; 15 min), 1064 nm (300 mW/cm^2^; 12 min), 1550 nm (300 mW/cm^2^; 12 min), and 2240 nm (300 mW/cm^2^; 12 min) light under the same conditions. After photoirradiation, the cells were trypsinized and resuspended in binding buffer (200 μL, 1×), followed by the addition of 5 μL of FITC labeled Annexin V and 10 μL of propidium iodide and incubated for 15 min at room temperature in the dark. Thereafter, the cells were analyzed by a flow cytometry analysis.

### 5.10 In Vivo Phototherapy Experiments

All of the mice (female C57BL/6J mice, 4–6 weeks old) used in this study were purchased from the National Laboratory Animal Center, Taipei, Taiwan. The handling of mice was conducted with the approval of the National Tsing Hua University Animal Center (Institutional Animal Care and Use Committee, IACUC approval number 10543). For tumor inoculation, B16L6 melanoma cancer cells (2 × 10^7^ cells/mL) were suspended in the Matrigel (Corning; basement membrane matrix) and PBS (1:1 ratio PBS and Matrigel at ice-cold temperature) and injected in the right flank considered as the primary tumor (1 × 10^6^ cells) and left flank to be the remote tumor (1 × 10^5^ cells) regions of female C57BL/6J mice. When the tumor diameter in the mice reached a size of 3–4 mm, they were randomly divided into 13 groups. The mice were irradiated with 808 nm laser (300 mW/cm^2^, 15 min), 1064 nm laser (300 mW/cm^2^, 12 min), 1550 nm laser (300 mW/cm^2^, 12 min), and 2240 nm (300 mW/cm^2^, 12 min) at 300 mW/cm^2^ power. While the control group was monitored, no irradiation. Anti-OX40 (Bio X Cell, lot no: 78012001) was injected into the mice at a dose of 50 μg per mouse on days 1, 4, and 7, whereas LaB_6_–PEG–folate NPs were i.v. injected at a concentration of 50 mg/kg on days 1, 3, 5, and 7. The survival rates, body weights, and relative tumor volumes of both the primary and remote tumors were monitored throughout the therapy every day. To establish the lung metastases, B16L6 melanoma cancer cells (2 × 10^7^ cells/mL) were subcutaneously injected on a tail vein on day 5. The treatment was administered on days 1,3, 6, and 9 up to 2 weeks. Anti-OX40 was injected intraperitoneally into the mice at 50 μg/mouse on days 1, 4, and 8. The mice were sacrificed on day 16 and the metastasis nudes of all of the mouse groups were counted to evaluate the antimetastasis effect.

### 5.11 In Vivo Photothermal Imaging

Mice bearing B16L6 melanoma tumors were injected intravenously with LaB_6_–PEG–folate NPs (50 mg/kg) and PBS were injected the next day after 24 h, followed by irradiation with 808 nm (300 mW/cm^2^, 15 min), 1064 nm (300 mW/cm^2^, 12 min), 1550 nm (300 mW/cm^2^, 12 min), and 2240 nm (300 mW/cm^2^, 12 min) diode lasers. A NIR thermal camera was used to monitor the temperature change near tumor regions, and the data was processed using TAS 19 software.

### 5.12 In Vivo Pharmacokinetic Study

A concentration of 50 mg/kg of LaB_6_–PEG–folate NPs, LaB_6_ NPs, and FDA-approved chemodrug DOX, respectively, was intravenously injected on healthy mice (female C57BL/6J mice). At different time points 1, 3, 6, 12, 24, 36, 48, and 60 h after iv injection, blood samples were collected from ocular veins and then immersed in aqua regia, digested, and analyzed using ICP–MS.

### 5.13 In Vivo Blood Chemistry Analysis

Biochemical analysis was performed by collecting blood samples from ocular veins of healthy mice. Serum samples were collected from the blood by centrifugation at 3000 rpm for 15 min. Blood chemistry was determined using an automated biochemical analyzer (Type 7200-202; Hitachi Ltd., Tokyo, Japan).

### 5.14 H&E Tissue Sectioning and Caspase 3 Staining

To analyze the extent of damages, the side organs, including liver, spleen, heart, kidney, and tumor tissues, were dissected from the mice treated with different lasers, collected, and stained with hematoxylin and eosin (H&E). Further, 50 μm depth from each organ was sectioned and stained with H&E and examined for the tissue damages post-phototherapy treatment.

### 5.15 Biodistribution for LaB_6_–PEG–Folate NPs in the Main Organs

50 mg/kg of LaB_6_–PEG–folate NPs (50 mg/kg) were intravenously injected. After 12 and 24 h postinjection, various organs of the mice (tumor, liver, kidney, spleen, heart, blood, and pancreas) were collected. The organs were digested using HClO_4_ (60%) and H_2_O_2_ were added to each organ in a 1:2 vol %. Then the contents were heated at 60–80 °C, followed by sonication for 24–36 h.

### 5.16 Cytokine Analysis and Detection

The serum was collected from mice in the different treatment groups after the irradiation experiments. The concentrations of the cytokines, including IFN-γ (R&D Systems, lot no: P138913), TNF-α (R&D Systems, lot no: P190320), and IL-2 (R&D Systems, lot no: P226350), were analyzed using the ELISA kits (Proteintech, USA).

### 5.17 DC Cell Maturation Analysis

Mice were injected with LaB_6_–PEG–folate NPs (intravenously) and anti-OX40 (intraperitoneal injection). Nine days post-injection, lymph nodes were collected from mice in various treatment groups, stained with DC biomarker CD11c (BD Biosciences, lot No: 9345738), CD 80 (BD Biosciences, lot No: 9276231), and CD 86 (BD Biosciences, lot No: 9301674) antibodies and subsequently analyzed using flow cytometry.

### 5.18 Analysis of the Proliferation and Differentiation of Immune Cells

For the detection of immune activation, mice bearing B16L6 tumors were injected intravenously with LaB_6_–PEG–folate NPs (50 mg/kg). On the 14th day after NIR light irradiation, the mice were sacrificed, and cells were harvested from the spleen and the tumors for NK cell analysis from different treatment groups. The blocking and staining of cells were conducted according to the manufacturer’s instructions and then CD45, CD49b, NKG2G, NKp46, F4/80, CD86, and CD206 expressing cells were analyzed using a flow cytometer.

### 5.19 Flow Cytometry Analysis

To analyze the tumor infiltration of T cells in the B16L6 melanoma tumors, the mice were sacrificed by dislocation on days 12th to 14th post-tumor inoculation. Briefly, the tumor was washed with 1× PBS, cut into small pieces, and transferred to the 1× PBS solution, followed by centrifuging at 2000 rpm at 4 °C for 10 min. Then, to the supernatant were added 1× PBS and 1 mL of dispase (25×), and the mixture was stirred for 30 min, filtered using a 70 μm filter, and washed again. To the supernatant, 5 mL of 1× RBC lysis buffer was added with 5 mL of 1× PBS and centrifuged again. Then, 1.2 mL of 1× PBS (containing 1% goat serum and 0.2% Fc block) was placed for 30 min in ice. Then, each sample was separated and stained for 40 min in ice with antimouse CD8 (BD bioscience 553033), CD45 (BD bioscience 559864), and CD4 (BD bioscience 553650) antibodies, respectively. The samples were disclosed by flow cytometry (B Accuri C6) for the number of infiltrations CD4^+^CD45^+^ T cells and CD8^+^ T cells.

### 5.20 NKT Cell Analysis in Tumor Tissues

To evaluate the NKT cell population in remote B16L6 melanoma tumors, mice were sacrificed by cervical dislocation on day 14 post-tumor inoculation. The tumors were washed with 1× PBS, minced into small fragments, and suspended in 1× PBS, followed by centrifugation at 2000 rpm for 10 min at 4 °C. The pellet was resuspended in 1× PBS containing 1 mL of 25× dispase, incubated with gentle stirring for 30 min, and passed through a 70 μm cell strainer. The filtrate was washed and treated with 5 mL of 1× RBC lysis buffer mixed with 5 mL of 1× PBS, then centrifuged again. The resulting pellet was resuspended in 1.2 mL of 1× PBS supplemented with 1% goat serum and 0.2% Fc block and incubated on ice for 30 min. Single-cell suspensions were subsequently stained with fluorochrome-conjugated antimouse CD3 and NK1.1 antibodies for 40 min on ice. Flow cytometric analysis was performed, and CD3^+^NK1.1^+^ populations were defined as the NKT cells. Data were analyzed using FlowJo software.

### 5.21 In Vitro Analysis of Immunogenic Cell Death (ICD) Markers

Ecto-calreticulin (CRT) expressions were evaluated using a confocal laser scanning microscope (CLSM). Two × 10^5^ cells/mL of B16BL6 cells were cultured on a 6-well plate and incubated for 24 h. LaB_6_–PEG–folate NPs (100 μg/mL) were added to cells and incubated for 12 h. Then, the cells were photoirradiated with 808 nm (300 mW/cm^2^; 15 min), 1064 nm (300 mW/cm^2^; 12 min), 1550 nm (300 mW/cm^2^; 12 min), and 2240 nm (300 mW/cm^2^; 12 min) light, respectively. After irradiation, the cells were washed with PBS, subsequently the CRT antibody (1:100 dilution; Abcam, Ab196159) was added and the cells were incubated for another 30 min in room temperature and finally visualized using CLSM. Similar to detecting adenosine triphosphate (ATP) and high mobility group box 1 protein (HMGB1), the cell supernatant was collected and ATP release was examined using an ATP Assay kit-luminescence (Dojindo laboratories, WU821) and the HMGB1 was detected using an enzyme-linked immunosorbent assay (ELISA) Kit (Arigo. Biolaboratories, 130814), respectively.

### 5.22 Cytokine Analysis and Detection

The serum was collected from mice in the different treatment groups after the irradiation experiments. The concentrations of the cytokines, including IFN-γ, TNF-α, TNF-β, IL-2, IL-12, and IL-10, were analyzed using ELISA kits.

### 5.23 Measurement of Enhanced Cellular Uptake of Nanoparticles by Surface-Chelated Folate Moieties

B16BL6 cells (2 × 10^5^ cells/mL) were seeded into 6-well plates and incubated for 24 h under standard culture conditions. Subsequently, the cells were treated with various concentrations of LaB_6_–PEG–folate NPs or citrate-coated LaB_6_ NPs, followed by incubation for an additional 24 h. After incubation, the cells were washed thoroughly with 1× PBS, and subjected to trypsinization for detachment. The cells were stained with a Cy5 goat antimouse IgG secondary antibody (Lot No. 1675775, Life Technologies Corporation, USA) at a concentration of 5 μg/mL per flow tube and incubated at 4 °C for 1 h. After staining, cells were washed thoroughly with PBS to remove any unbound antibody and resuspended in 1 mL of 1× PBS. Flow cytometric analysis was performed using APC-A channel settings (λ_ex_ = 650 nm; λ_em_ = 667 nm) to detect the fluorescence signal corresponding to nanoparticle binding and uptake.

### 5.24 Assessment of T-Cell Proliferation by the *K*_i_-67 Expression

To assess T-cell proliferation following various treatments, spleens were harvested from B16BL6 tumor-bearing mice 12th post-treatment. Single-cell suspensions were prepared by mechanical dissociation and filtration through a 70 μm cell strainer. Red blood cells were removed using RBC lysis buffer following the manufacturer’s protocol. After the lysis, cells were washed and resuspended in FACS buffer. Surface staining was performed using antimouse CD45 and CD8 antibodies for 30 min at 4 °C in the dark. Following surface staining, cells were fixed with ice-cold 70% methanol and incubated at −20 °C for 30 min to permeabilize the cells for intracellular staining. Subsequently, cells were washed and stained intracellularly with an anti-*K*_i_-67 antibody for 30 min at room temperature in the dark. Stained cells were acquired on a flow cytometer and data were analyzed using FlowJo software. T-cell proliferation was quantified as the percentage of CD45^+^CD8^+^ *K*_i_-67^+^ cells among the total splenocytes.

### 5.25 Quantification of IFN-γ-Secreting T Cells by the ELISpot Assay

The frequency of IFN-γ–secreting T cells was assessed using a mouse IFN-γ ELISpot Kit following the manufacturer’s instructions. Splenocytes were freshly isolated from B16BL6 tumor-bearing mice on day 12 post-tumor inoculation and plated at a density of 2 × 10^5^ cells per well into 96-well PVDF-bottom plates precoated with the antimouse IFN-γ capture antibody. To evaluate antigen-specific responses, splenocytes were cocultured with LaB_6_–PEG–folate-treated B16BL6 tumor cells, which had been irradiated at 2240 nm (300 mW/cm^2^) for 48 h. Following incubation, wells were thoroughly washed to remove nonadherent cells, and the biotinylated antimouse IFN-γ detection antibody was added for 1.5 h at room temperature. Plates were then incubated with a streptavidin–alkaline phosphatase (AP) conjugate for 1 h at room temperature. Spots were developed using the BCIP/NBT substrate solution. Each purple spot corresponds to an individual IFN-γ-producing cell.

The specifications for all antibodies, reagents, and ELISA kits are listed in Supporting Information, Tables S3–S5.

### 5.26 Statistical Analysis

Statistical analyses were performed using GraphPad Prism 5 software. Comparisons between two groups were conducted using unpaired two-tailed Student’s *t*-tests, while comparisons among multiple groups were evaluated by one-way ANOVA followed by the Tukey’s post-hoc test for multiple comparisons. Two-way ANOVA was applied where appropriate. Statistical significance was indicated as *p < 0.05; **p < 0.01; ***p < 0.001.

## Supplementary Material



## Notes

Measurement of singlet oxygen phosphorescence quantum yield, quantum yield for generation of hydroxyl radicals, determination of the relative fractions of hyperthermia-induced cancer cell deaths and ROS-induced cell deaths mediated by photoirradiation of LaB_6_–PEG–folate NPs, characterization of surface-functionalized LaB_6_ nanoparticles, singlet oxygen phosphorescence spectra, EPR spectra for generation of hydroxyl radicals from LaB6 NPs under different photoirradiation wavelengths, LIVE/DEAD assay of the LaB_6_–PEG–folate-internalized B16BL6 melanoma cancer cells under dark and photoirradiation conditions, and cell viabilities of a healthy human umbilical vein endothelial cells (HUVECs) as a function of the doses of LaB_6_–PEG–folate NPs in the dark (PDF)

## Abbreviations

HCC: hepatocellular carcinoma
ICI: immune-checkpoint inhibitor
NIR-IV PDT: NIR-IV photodynamic therapy
PEG: polyethylene glycol
anti-PD-1: antiprogrammed cell death protein 1
anti-CTLA-4: anticytotoxic T lymphocyte-associated protein 4
LaB_6_ NPs: lanthanum hexaboride nanoparticles
SEM: scanning electron microscopy
XRD: X-ray diffraction
EDC: 1-ethyl-3-(3-dimethylaminopropyl) carbodiimide
FTIR: Fourier transformed infrared spectroscopy
XPS: X-ray photoelectron spectroscopy
NIR: near infrared
TEMPO: 2,2,6,6-tetramethylpiperidine 1-oxyl, 2,2,6,6-tetramethyl-1-piperidinyloxy
DPBF: 1,3-diphenylisobenzofuran
DMPO: 5,5-dimethyl-1-pyrroline *N*-oxide
EPR: electron paramagnetic resonance spectroscopy
HSP70: Heat Shock Protein 70
ROS: reactive oxygen species
APF: aminophenyl fluorescein
DCF: dichlorofluorescein
DAMPs: damage-associated molecular patterns
CRT: ecto-calreticulin
HMGB1: high mobility group box1
ICP–MS: inductively coupled plasma mass spectrometry
SCID: severe combined immunodeficiency disease
CAF: cancer-associated fibroblasts
ACT: adoptive cell therapy
TAMs: tumor-associated macrophages
ALP: alkaline phosphatase
ALT: alanine transaminase
AST: aspartate transaminase
BUN: blood urea nitrogen
CRE: creatinine
ALB: albumin
TME: tumor microenvironment
HBV: hepatitis B virus
HCV: hepatitis C virus
PD-1: programmed death receptor.
