# Supplementary material for: 2240 nm NIR-IV Photodynamic Therapy Can Reverse Ineffective Anti-OX40 Cancer Immunotherapy to Become Effective
Source: ACS Nano. 2025 Oct 11;19(41):36129–47. doi: 10.1021/acsnano.5c04323 (PMC12548349; doi:10.1021/acsnano.5c04323)
Supplement: Supplementary file 1 [file nn5c04323_si_001.pdf]

Supporting Information

2240 nm NIR-IV Photodynamic Therapy Can Reverse Ineffective Anti-OX40 Cancer Immunotherapy to Become Effective

Munusamy Shanmugam[1], Chi-Shiun Chiang,[2] and Kuo Chu Hwang[1]*

[1.]Dr. Munusamy Shanmugam and Prof. Kuo Chu Hwang*
Department of Chemistry
National Tsing Hua University, Hsinchu 30013, Taiwan ROC
E-mail: kchwang@mx.nthu.edu.tw

[2.]Prof. Chi-Shiun Chiang
Department of Biomedical Engineering and Environmental Sciences
National Tsing Hua University, Hsinchu 30013, Taiwan ROC

Singlet oxygen phosphorescence quantum yield

Singlet oxygen phosphorescence quatam yield at 1064 nm was measured and calculated as follows. A well known dye methylene blue (MB) was used as a reference standard for comparison and measurement of singlet O_2 phosphorescence quantum yield via comparing the phosphorescence emission area (by integrating from 1200 to 1300 nm) of singlet oxygen to that for MB (Area $_{phos\text{-}MB}$) using the equation (1) below:

$\Phi_{LaB6\text{-}1064}/\Phi_{MB} = [Area_{phos\text{-}LaB6\text{-}1064}/(Ab_{LaB6\text{-}1064} \text{ x } I_{1064})] \text{ x } [Area_{phos\text{-}MB}/(Ab_{MB\text{-}650} \text{ x } I_{650})]$ .... (1)

Since $Area_{phos\text{-}MB} = Ab_{MB\text{-}650} \text{ x } I_{650} \text{ x } \Phi_{MB}$ …………… (2), and

$Area_{phos\text{-}MB}$ = 43359661 (obtained via experimental measurement).

Similarly, we derived the equation for LaB_6-PEG-folate NPs.

$Area_{phos\text{-}LaB6\text{-}1064} = Ab_{LaB6\text{-}1064} \text{ x } I_{1064} \text{ x } \Phi_{LaB6\text{-}1064}$…………… (3)

$Area_{phos\text{-}LaB6\text{-}1064}$ = 50659525 (obtained via experimental measurement)

The incident light intensity of FLS 920 spectrometer at 1064 nm was 8.3 mW/cm^2 with slit width 10 nm. The absorbance values for both MB and LaB_6-PEG-folate NPs-containing solutions were adjusted to be 0.5 from the UV-visible NIR spectrometer. From litertature we obtained the quantum yield (Φ_{MB}) for methylene blue in H_2O to be 0.60[S1]. Singlet oxygen quatam yield of LaB_6- PEG-folate NPs was obtained by using the equations (1) to be $\Phi_{LaB6\text{-}1064}$ to be **0.22.**

Quantum yield for the generation of hydroxyl radicals

In the detection of hydroxyl radicals, non-fluorescent 3′-(*p*-aminophenyl) fluorescein (APF) was used to react with hydroxyl radicals, leading to formation of a fluorescent product. The quantum

yield for the generation of hydroxyl radicals under 2240 nm NIR light irradiation of a LaB_6-PEG-folate NPs-containing solution was calculated as follows. TiO_2 NPs used as a reference for for generation of hydroxyl radicals with a known quantum yield value of 0.04[S2]. The fluorescence area ($Area_{phos\text{-}TiO2+APF}$) for photo-irradiation of a-TiO_2 NPs-containing aqueous solution is,

$Area_{phos\text{-}TiO2+APF} = Ab_{TiO2\text{-}375\ nm} \times I_{375\ nm} \times \Phi_{TiO2\text{-}OH}$ .... (1)

$Area_{phos\text{-}TiO2+APF} = 2035349$

Similar equation can be derived for LaB_6-PEG-folate NPs

$Area_{phos\text{-}LaB6\text{-}2240\ +\ APF} = Ab_{\ LaB6\text{-}2240\ nm} \times I_{2240\ nm} \times \Phi_{\ LaB6\text{-}2240\ +\ OH}$.............. (2)

$Area_{phos\text{-}LaB6\text{-}2240\ +\ APF} = 9057337$

The fluorescence area was obtained integration of the fluorescence area between 500 to 600 nm. The absorbance values were maintained to be same from the UV-visible NIR spectrometer. From the photoluminescence spectrometer (FLS920) at a slit width of 3 nm used, the light intensity of Xe lamp is 8.3 mW/cm^2 at 375 nm, whereas it is 300 mW/cm^2 at 808, 1064, 1550 and 2240 nm. Hydroxyl radical quantum yield ($\Phi_{TiO2\text{-}OH}$) for TiO_2 NPs in water is 0.040[S2]. The Hydroxyl radical formation quantum yield of LaB_6-PEG-folate NPs are obtained by dividing the equations (1)/(2) and then by substituting the corresponding values in the above equations. The $\Phi_{\ LaB6\text{-}2240\text{-}OH}$ obtained is **0.257.**

Similarly, $Area_{phos\text{-}LaB6\text{-}1550\ +\ APF} = 1657895$, $\Phi_{\ LaB6\text{-}1550\ -\ OH}$ obtained is **0.238**

$Area_{phos\text{-}LaB6\text{-}1064\ +\ APF} = 1657895$, $\Phi_{\ LaB6\text{-}1064\ -\ OH}$ obtained is **0.043**

$Area_{phos\text{-}LaB6\text{-}808\ +\ APF} = 807632$, $\Phi_{\ LaB6\text{-}808\ -\ OH}$ obtained is **0.019**

$Area_{phos\text{-}LaB6\text{-}2240\ +\ APF} = 807632$, $\Phi_{\ LaB6\text{-}2240\text{-}OH}$ obtained is **0.257.**

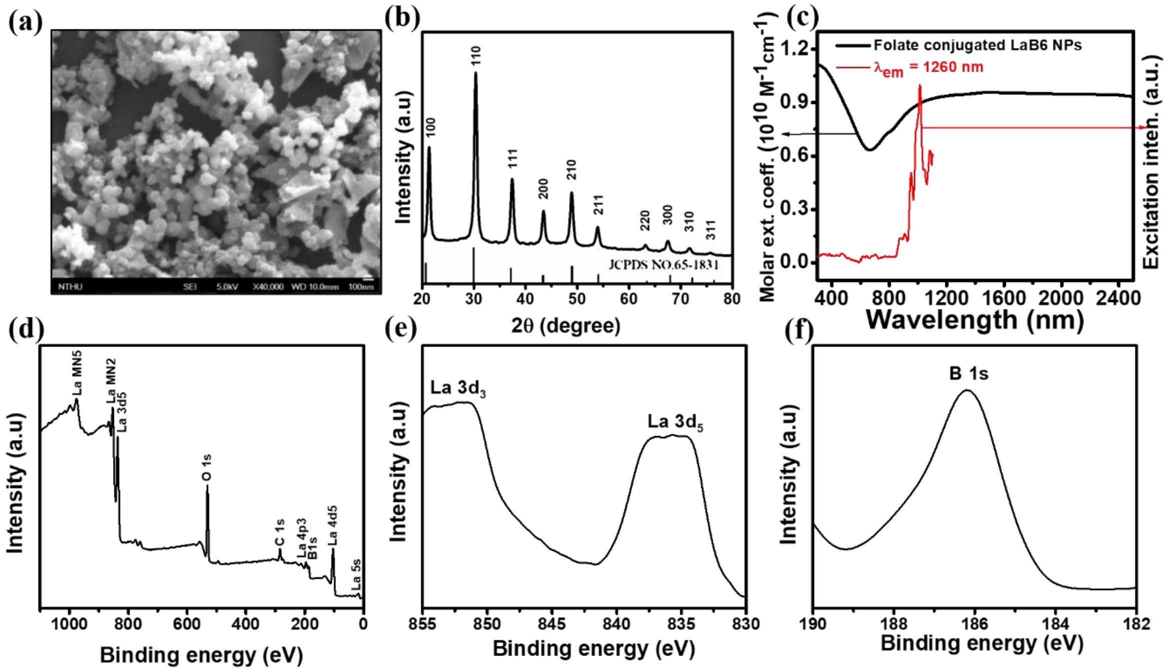

Figure S1. Characterization of LaB_6 NPs. (a) SEM image, (b) XRD spectra of LaB_6 NPs. (c) UV-visible-NIR absorption spectrum and excitation spectrum (red curve) for singlet oxygen phosphorescence of PEG-folate LaB_6 NPs (λ_{em}@ 1260 nm). (d) X-ray photoelectron spectroscopy (XPS) full length survey scan of as-prepared LaB_6 NPs. (e) La_{3d} binding energy spectrum. (f) B_{1s} binding energy spectrum.

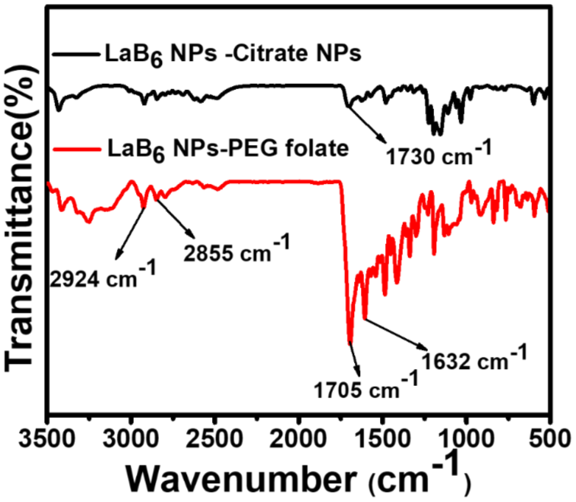

Figure S2. FTIR spectra of LaB_6-citrate, and LaB_6-PEG-folate NPs.

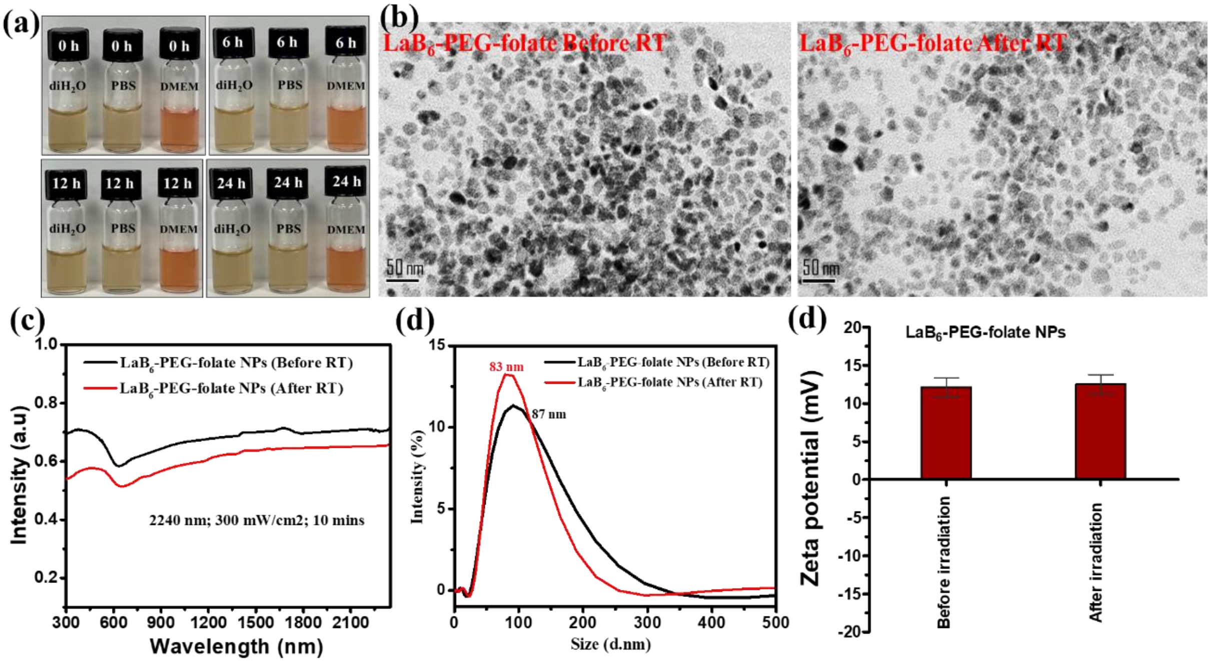

Figure S3. Stability of LaB$_6$-PEG-folate NPs before and after irradiation (a) In different biological media (PBS and DMEM) over time intervals of 0, 6, 12, and 24 h (b) TEM images, (c) UV-vis-NIR spectroscopy, d) DLS measurements, and (d) Zeta potential.

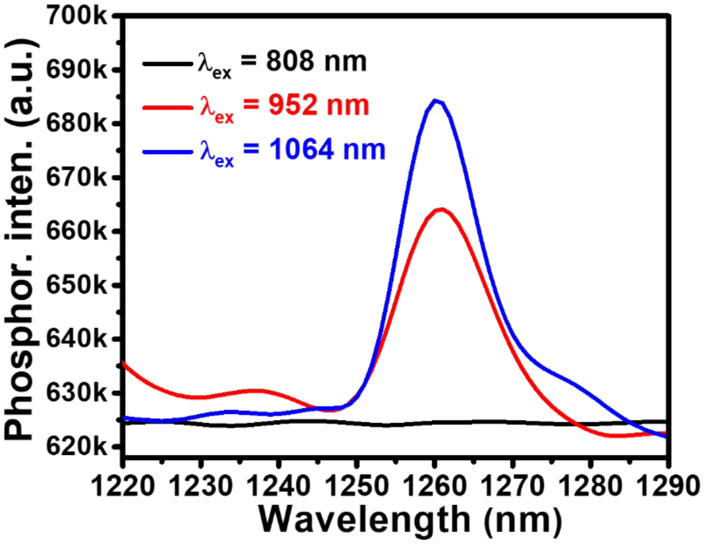

Figure S4. Singlet oxygen phosphorescence emission spectra from a LaB$_6$-PEG-folate NPs-containing aqueous solution upon different NIR light (808, 952 and 1064 nm) photo-excitation.

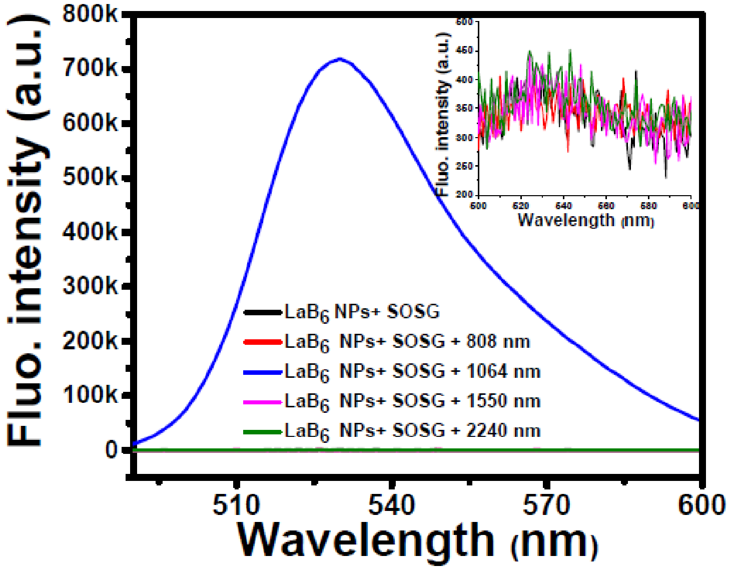

Figure S5. Fluorescence emission spectra from solutions containing both singlet oxygen sensor green (SOSG) and LaB_6-PEG-folate NPs under different photo irradiation conditions.

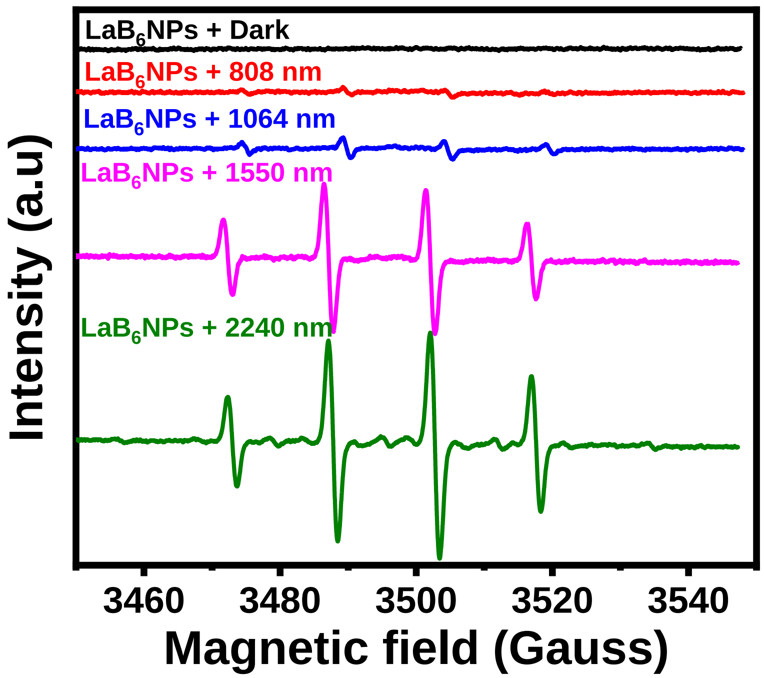

Figure S6. EPR spectra of DMPO-OH spin adduct generated from a LaB_6-PEG-folate NPs-containing aqueous solution under 808, 1064, 1550 and 2240 nm NIR light irradiation, respectively.

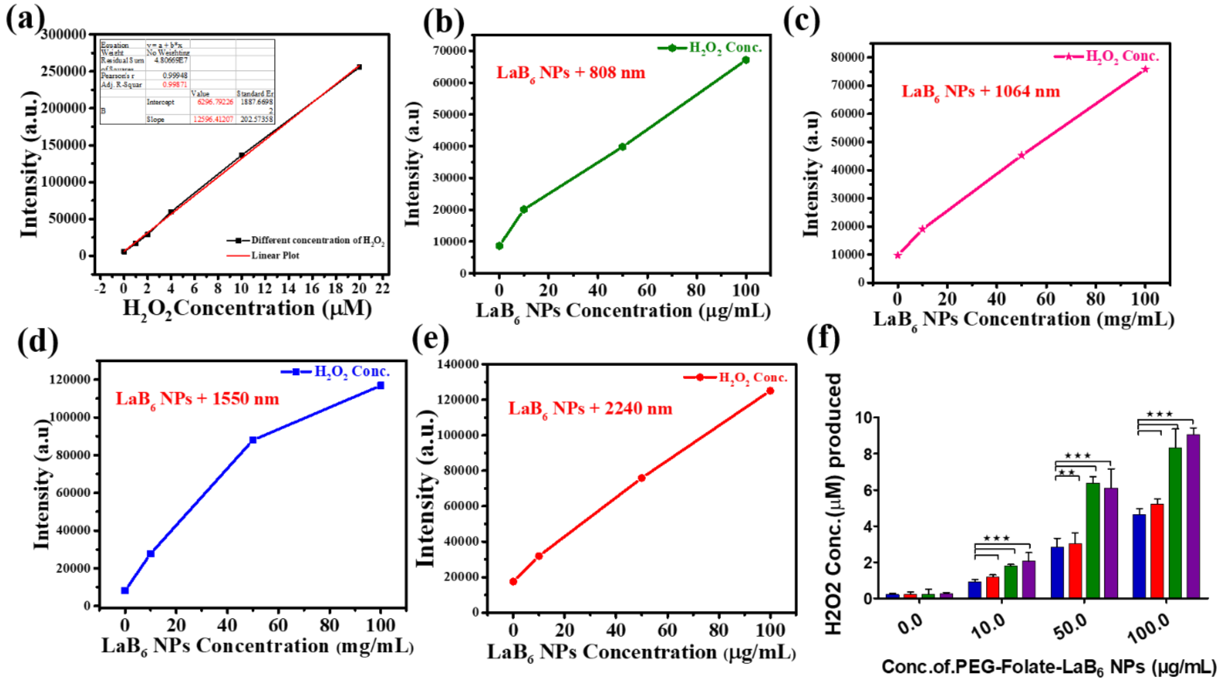

Figure S7. Detection of H_2O_2 formation using an Amplex red assay. (a) The standard curve was created using different amounts of externally added H_2O_2 (0, 1, 2, 4, 10, and 20 μM). The Amplex red fluorescence intensities were obtained from photo-irradiation of LaB_6-PEG-folate NPs by (b) 808 nm (300 mW/cm^2; 15 min), (c) 1064 nm (300 mW/cm^2;12 min) (d) 1550 nm 300 mW/cm^2; 12 min), and (e) 2240 nm (300 mW/cm^2; 12 min). (f) The corresponding amounts of H_2O_2 calculated by comparing the fluorescence intensities to the standard curve shown in (a). Data are expressed as mean ± s.d. (n = 2). One-way ANOVA with Tukey's post-hoc test was used to determine statistical significance. *$P < 0.05$, **$P < 0.01$, ***$P < 0.001$, comparing treatment groups to control and among selected group pairs as indicated.

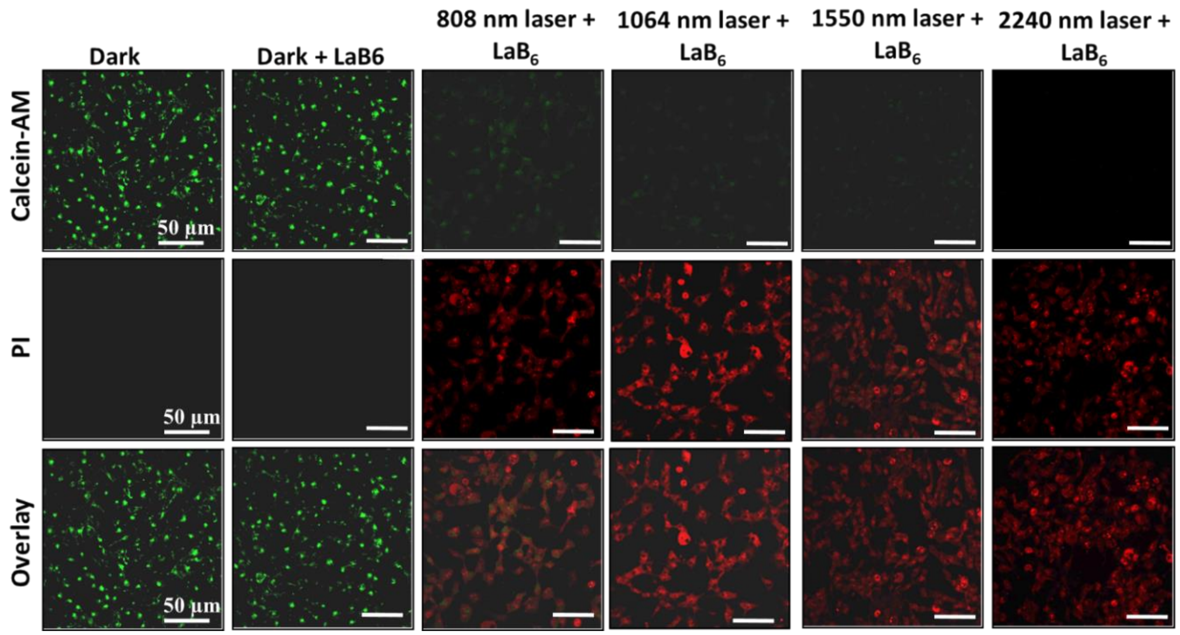

Figure S8. LIVE/DEAD assay of the LaB_6-PEG-folate internalized B16BL6 melanoma cancer cells under dark and photo-irradiation conditions. The live cells were stained with Calcein AM (green color), and the dead cells were stained with PI (red color), respectively. The scale bar represents 50 µm.

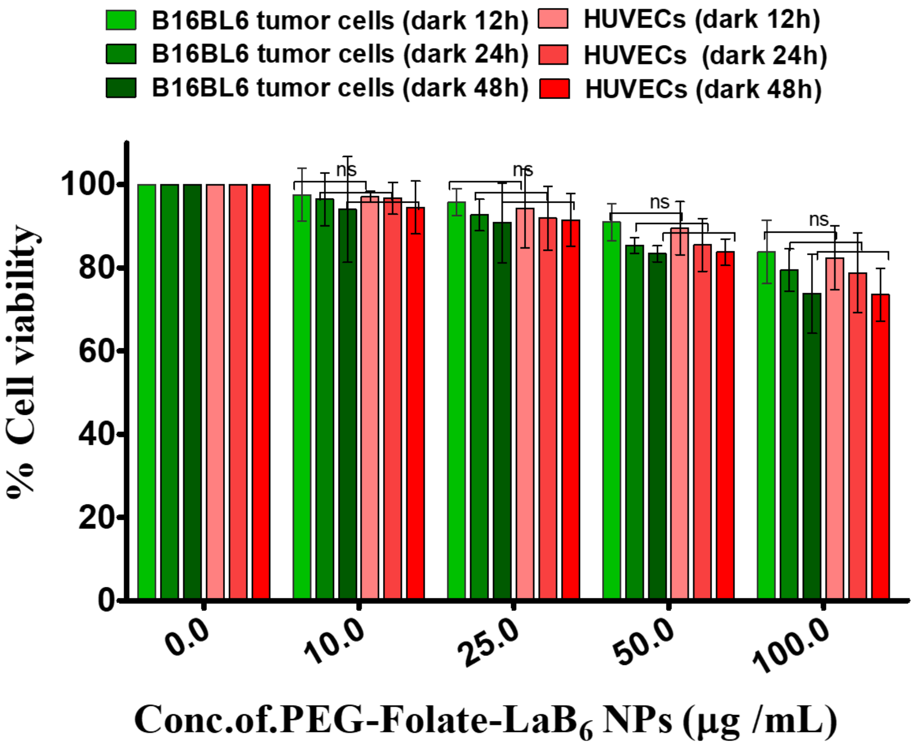

Figure S9. **Cell viabilities of a healthy human umbilical vein endothelial cells (HUVECs)** as a function of the doses of LaB_6-PEG-folate NPs in dark. Data are expressed as mean ± s.d. (n = 2). One-way ANOVA with Tukey's post-hoc test was used to determine statistical significance. *P < 0.05, **P < 0.01, ***P < 0.001.

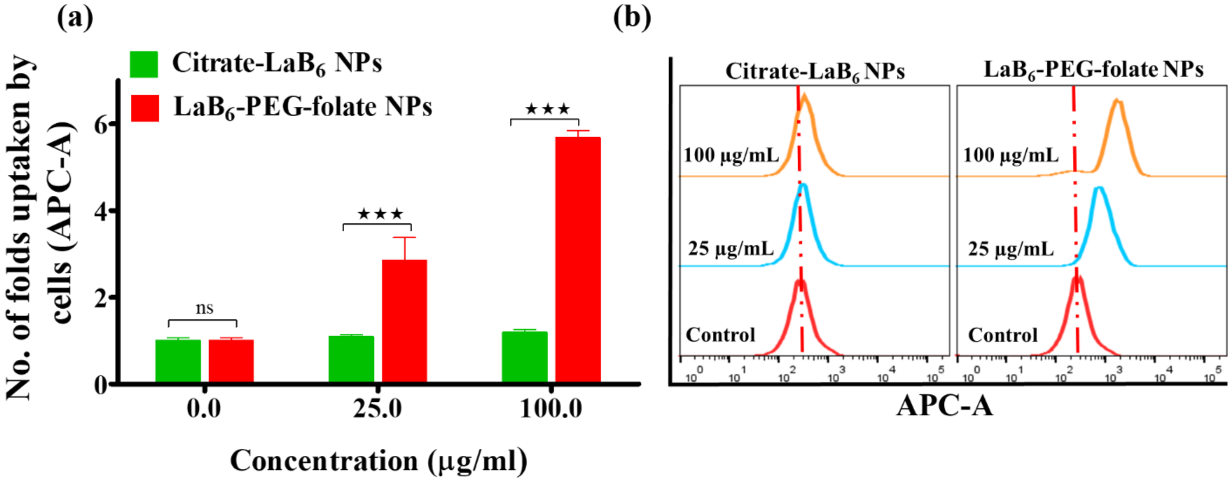

Figure S10. Targeting ability of LaB_6-PEG-folate NPs and citrate- LaB_6 NPs. (a) Flow cytometry experiments and b) Fluorescence intensity (APC-A channel) changes with respect to concentration of Citrate-LaB_6NPs and LaB_6-PEG-folate NPs NPs. Data are expressed as mean ± s.d. (n = 2). One-way ANOVA with Tukey's post-hoc test was used to determine statistical significance. *P < 0.05, **P < 0.01, ***P < 0.001.

Determination of the relative fractions of hyperthermia-induced cancer cell deaths and ROS-induced cell deaths mediated by photo irradiation of LaB_6-PEG-folate NPs.

To determine the relative contribution of hyperthermia-induced cell deaths and ROS-induced cell deaths, the cell viability measurements were conducted at both 37 °C and 4 °C for all phototherapy groups. Since hyperthermia-induced cell deaths are highly sensitive to the local temperature and will be suppressed by lowering down the medium temperature from 37 to 4 °C, whereas the ROS-induced cell deaths are very weakly dependent on the local temperature due to the very high chemical reactivities of ROS. Upon 808 nm NIR light irradiation, the LaB_6-PEG-folate NPs releases nearly all the excited energy to heat and mediates nearly pure photothermal therapy effect on killing cancer cells (as stated in the main text). Upon lowering down the medium temperature from 37 to 4 °C, the 808 nm NIR light mediated NIR-I PTT-mediated caner cell deaths decreases from 30% at 37 °C (at a LaB_6-PEG-folate NPs dose of 50 mg/kg, see data in Figure 1f)) to 20% at 4 °C (at a LaB_6-PEG-folate NPs dose of 50 mg/kg, see data in Figure S11). The suppression factor is 0.33, that is, upon lowering down the medium temperature from 37 °C to 4 °C, the hyperthermia-induced cell deaths will be reduced by 1/3. Whereas the ROS-induced cell deaths are assumed to

remain the same for both 37 °C and 4 °C. By knowing the temperature-induced suppression factors for both hyperthermia-induced cell deaths and ROS-induced cell deaths, one can compare amounts of decrease in the cell viabilities data shown in Figure 1f (at 37 °C and a LaB_6-PEG-folate NPs dose of 50 mg/kg), and those in Figure S9 (at 4 °C and a LaB_6-PEG-folate NPs dose of 50 mg/kg) for all (808, 1064 nm, 1550 nm, and 2240 nm) phototherapy conditions, and obtained the relative contributions of hyperthermia-induced cell deaths and ROS-induced cell deaths for all photo-irradiation wavelengths, as shown in Figure S12.

The fractions were calculated as shown in the equations below:

$$(d_1)_{\text{cell death,1064 nm, 37 °C}} = (PDT)_{\text{1064 nm, 37 °C}} + (PTT)_{\text{1064 nm, 37 °C}} \text{ -------------- } (1)$$

$$(d_2)_{\text{cell death,1064 nm, 4 °C}} = (PDT)_{\text{1064 nm, 4 °C}} + (PTT)_{\text{1064 nm, 4 °C}} \text{ -------------- } (2)$$

$$d_3 = PTT_{\text{808 nm, 37 °C}} \text{ ------------------------------------ } (3)$$

$$d_4 = PTT_{\text{808 nm, 4 °C}} \text{ ------------------------------------- } (4)$$

The values of d_1, d_2, d_3 and d_4 were determined experimentally.

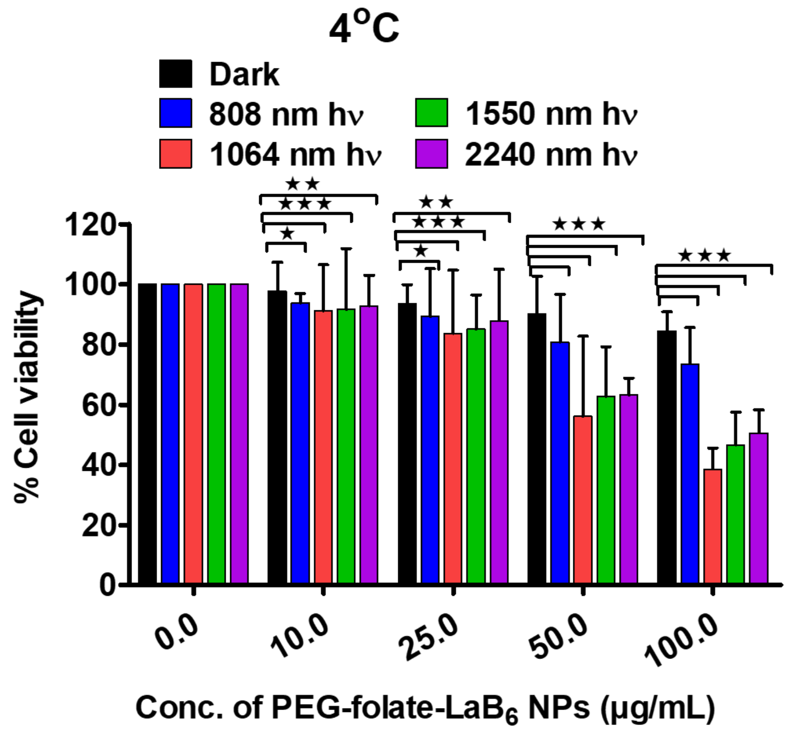

Figure S11. **Cellular viabilities** of LaB_6-PEG-folate NPs-internalized B16BL6 melanoma cancer cells at 4 ^{0}C. *p < 0.05; **p < 0.01; ***p < 0.001. Data are expressed as mean ± s.d. (n = 2). One-way ANOVA with Tukey's post-hoc test was used to determine statistical significance. *P < 0.05, **P < 0.01, ***P < 0.001.

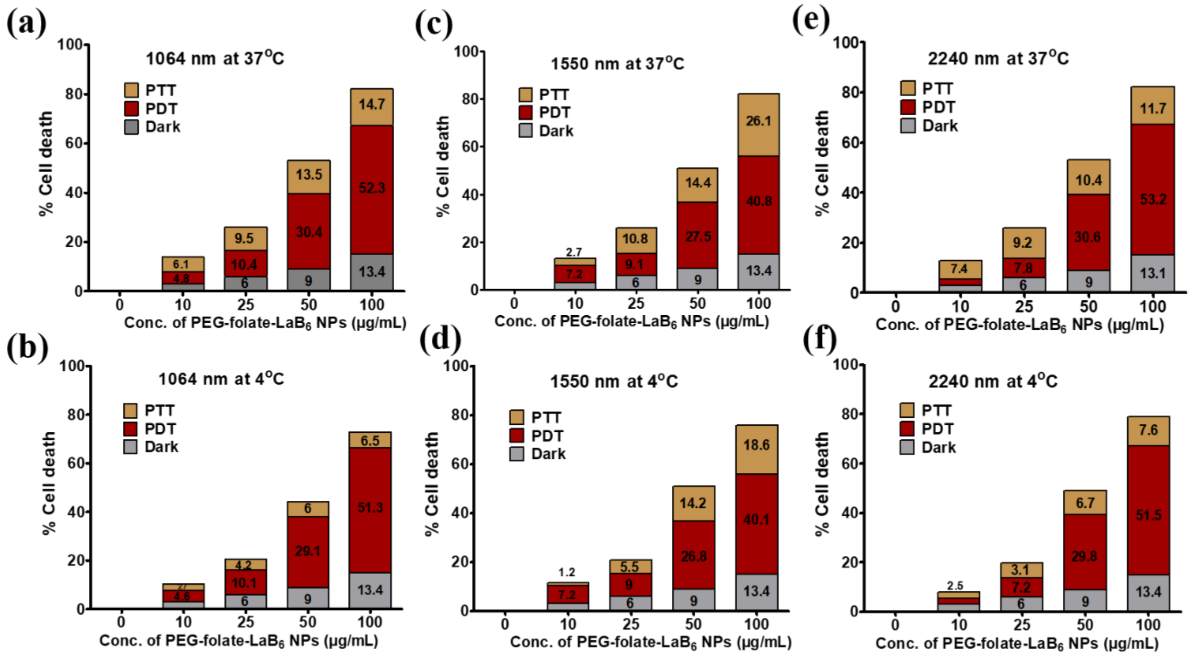

Figure S12. The relative contribution of NIR PDT and NIR PTT induced cellular deaths for LaB_6-PEG-folate NPs-internalized B16BL6 melanoma cancer cells upon (a) and (b)1064 nm, (c) and (d) 1550 nm, (e) and (f) 2240 nm NIR light irradiation at 37 °C and 4 °C, respectively.

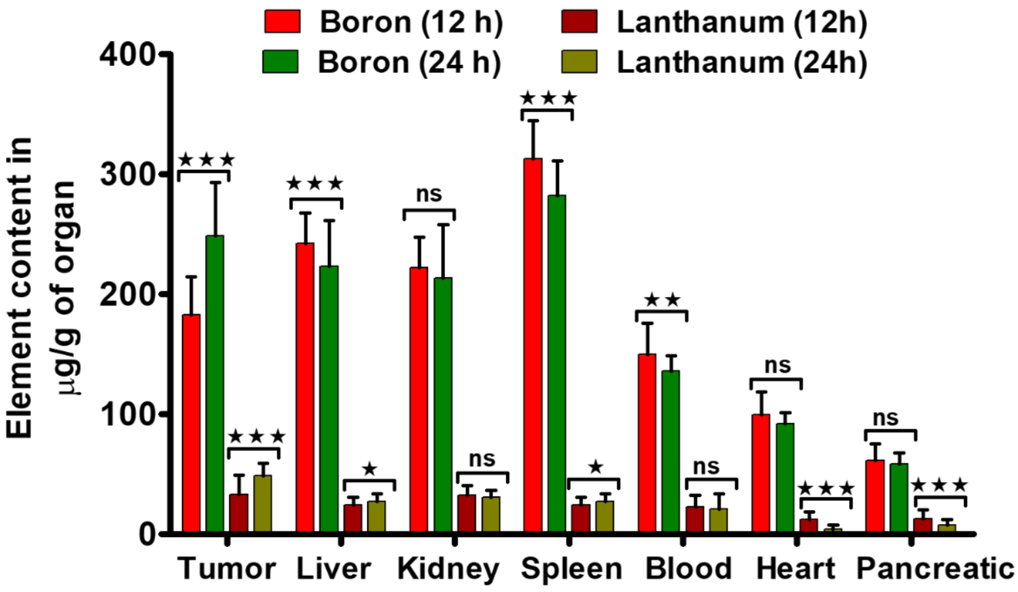

Figure S13. In vivo biodistribution of LaB_6-PEG-folate NPs internalized B16BL6 melanoma cacner cells using ICP-MS analysis at 12 and 24 h post i.v. injection. *p < 0.05; **p < 0.01; ***p

< 0.001. Data are expressed as mean ± s.d. (n = 2). One-way ANOVA with Tukey's post-hoc test was used to determine statistical significance. *P < 0.05, **P < 0.01, ***P < 0.001.

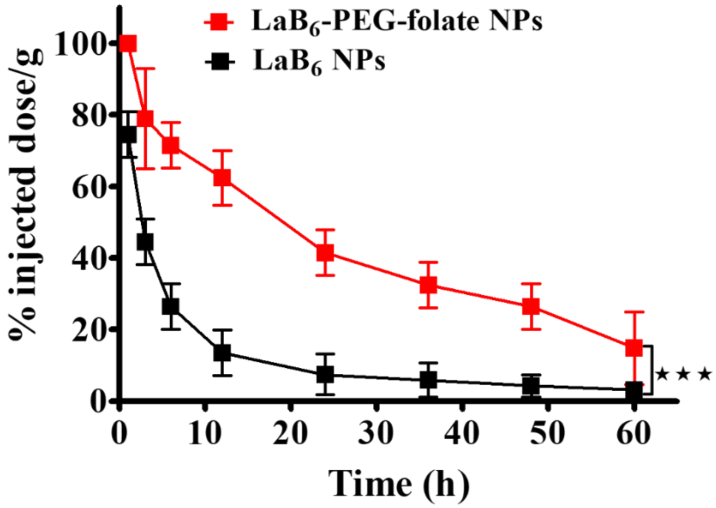

Figure S14. In vivo pharmacokinetic studies of LaB_6-PEG-folate NPs and LaB_6 NPs (without surface modification with PEG-folate) using healthy mice at various time points 1, 3, 6, 12, 24, 36, 48 and 60 h post iv injection of nanoparticles. Data are expressed as mean ± s.d. (n = 2). One-way ANOVA with Tukey's post-hoc test was used to determine statistical significance. *P < 0.05, **P < 0.01, ***P < 0.001.

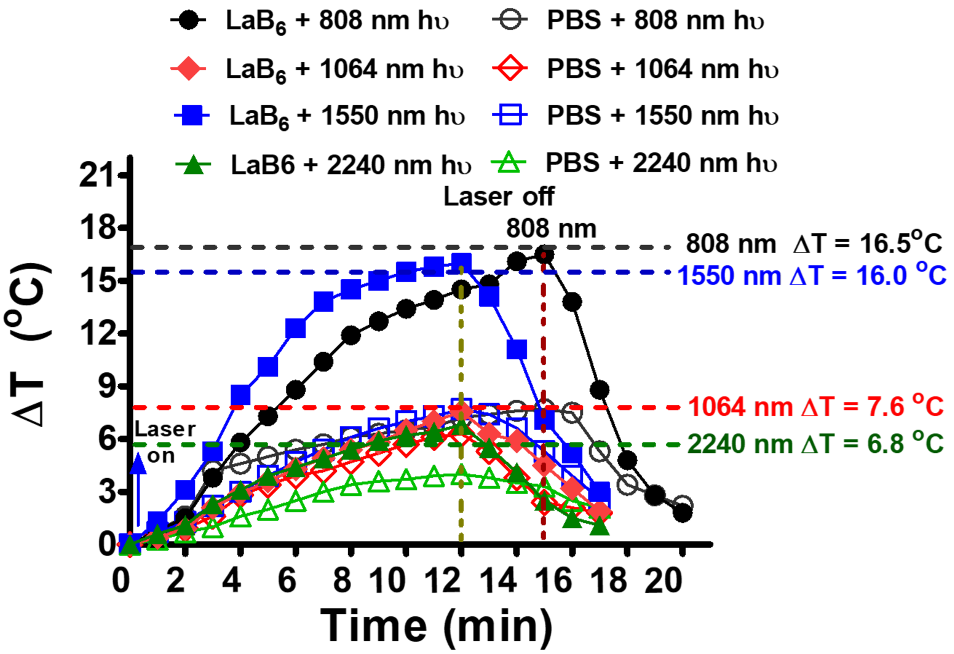

Figure S15. Temperature changes at tumor sites as a function of laser irradiation time for different photo-irradiation treatment conditions.

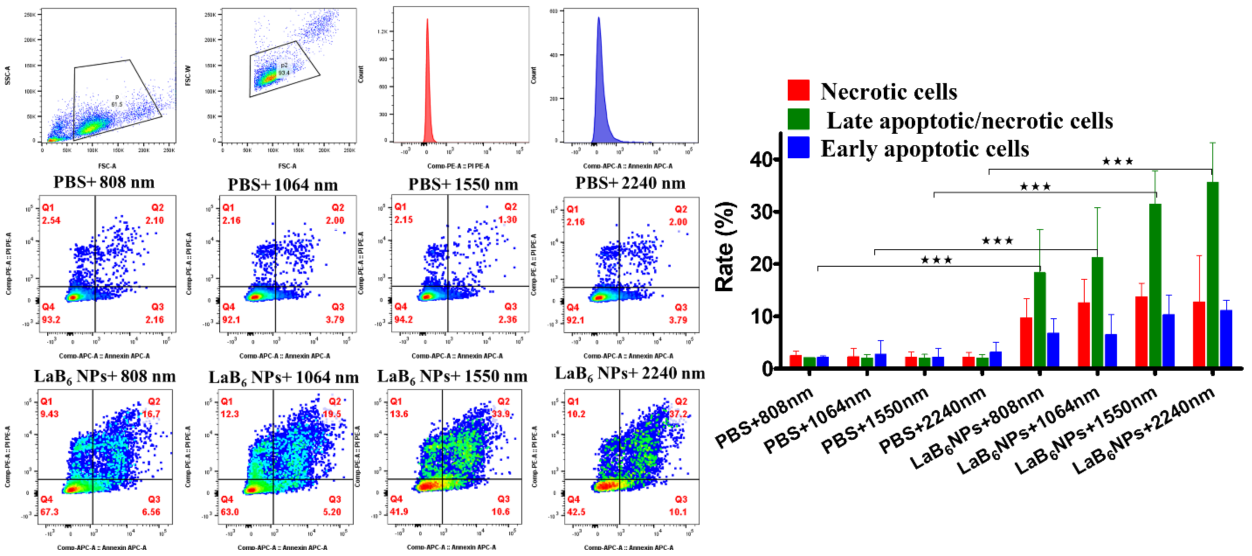

Figure S16: Representative flow cytometry results and quantitative analyses of the population of Annexin V and PI in B16BL6 cells after photo irradiation treatment. Quadrant statistics were used to study groups; each analysis recorded 10000 events. The bar graphs show the group values are expressed as mean SD (* p < 0.05, ** p < 0.01, ***. P ≤ 0.001).

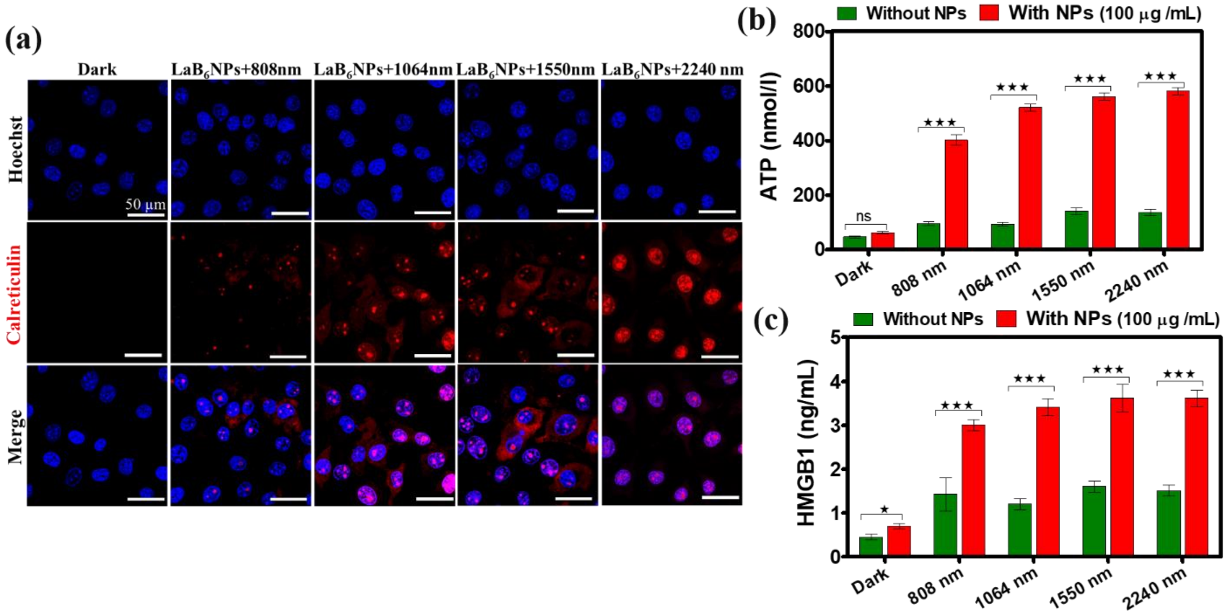

Figure S17: Photodynamic therapy-induced immunogenic cell deaths-associated biomarkers: (a) ecto-CRT immunofluorescence of various treatment groups; blue and red indicate nuclei and ecto-CRT, scale bars represented 50 µm; (b) ATP release and (c) HMGB1 release of different cells treated with and with LaB_6-PEG-folate NPs respectively. *p < 0.05; **p < 0.01; ***p < 0.001.

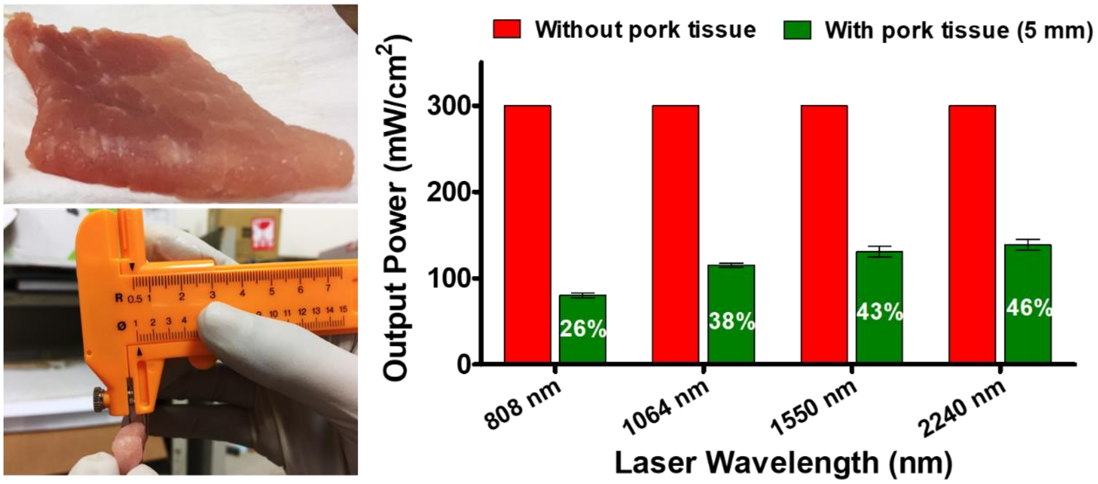

Figure S18. Comparison of penetration depths for different laser wavelengths (808, 1064, 1550 and 2240 nm) with and without using a pork tissue of 5 mm thickness at an output power intensity of 300 mW/cm^2. *p < 0.05; **p < 0.01; ***p < 0.001.

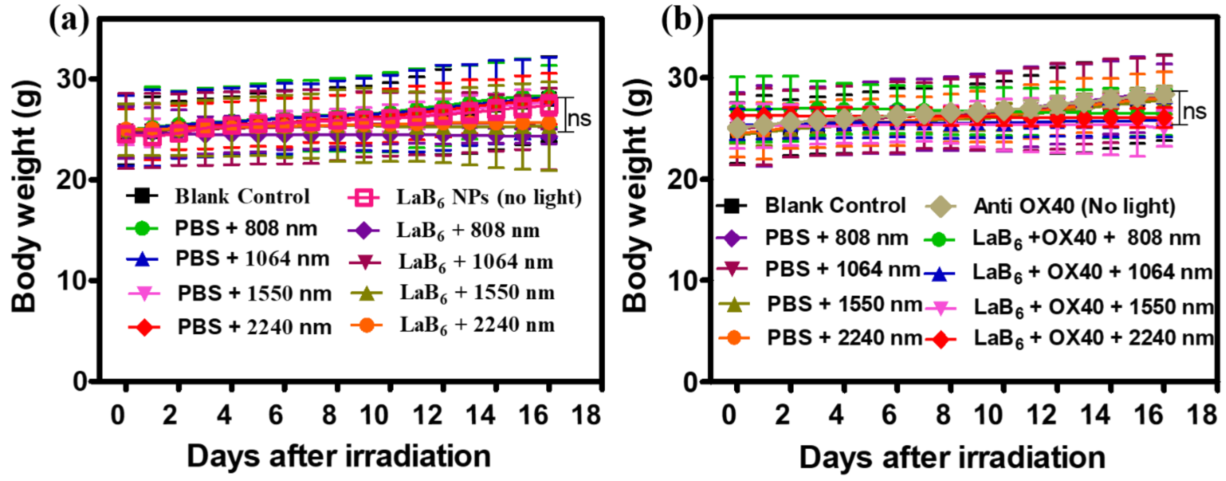

Figure S19. Body weights of mice bearing B16BL6 melanoma cancers and received different treatment conditions: (a) LaB_6-PEG-folate NPs+laser irradiation in the absence of anti-OX40, and (b) LaB_6-PEG-folate NPs+laser irradiation+anti-OX40. $^*p < 0.05$; $^{**}p < 0.01$; $^{***}p < 0.001$.

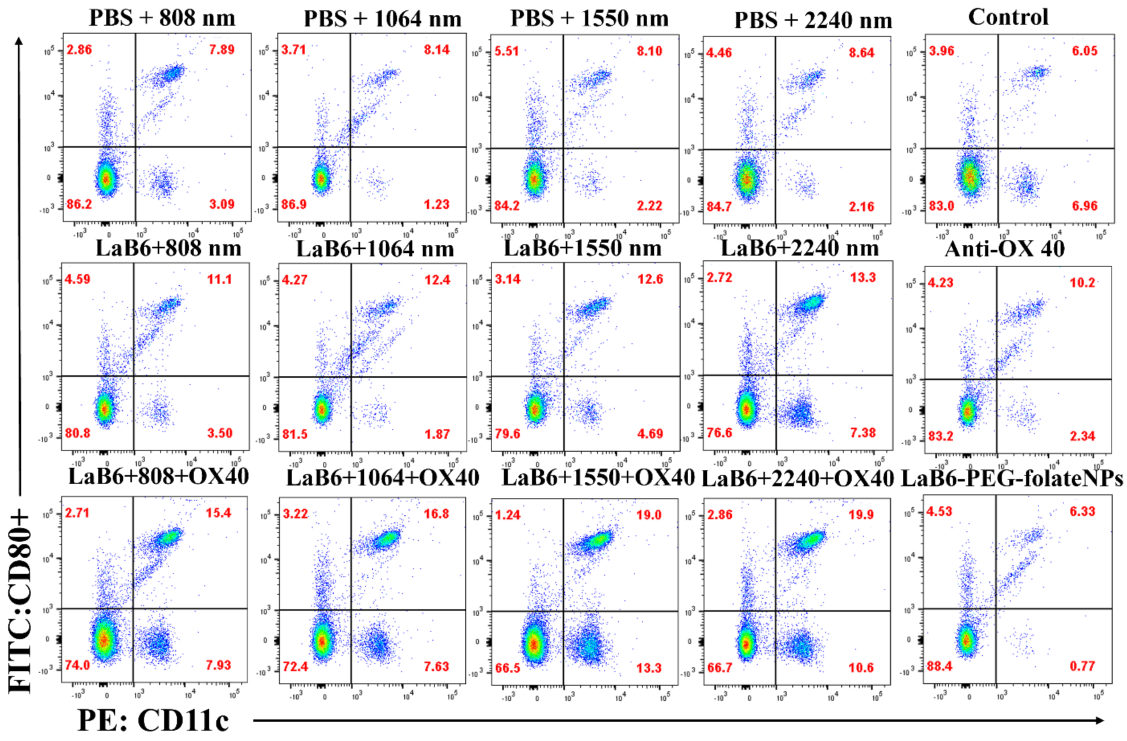

Figure S20. Flow cytometry analysis for DC maturation of B16L6-tumor-bearing mice after staining with anti-CD11c and anti-CD80 antibodies.

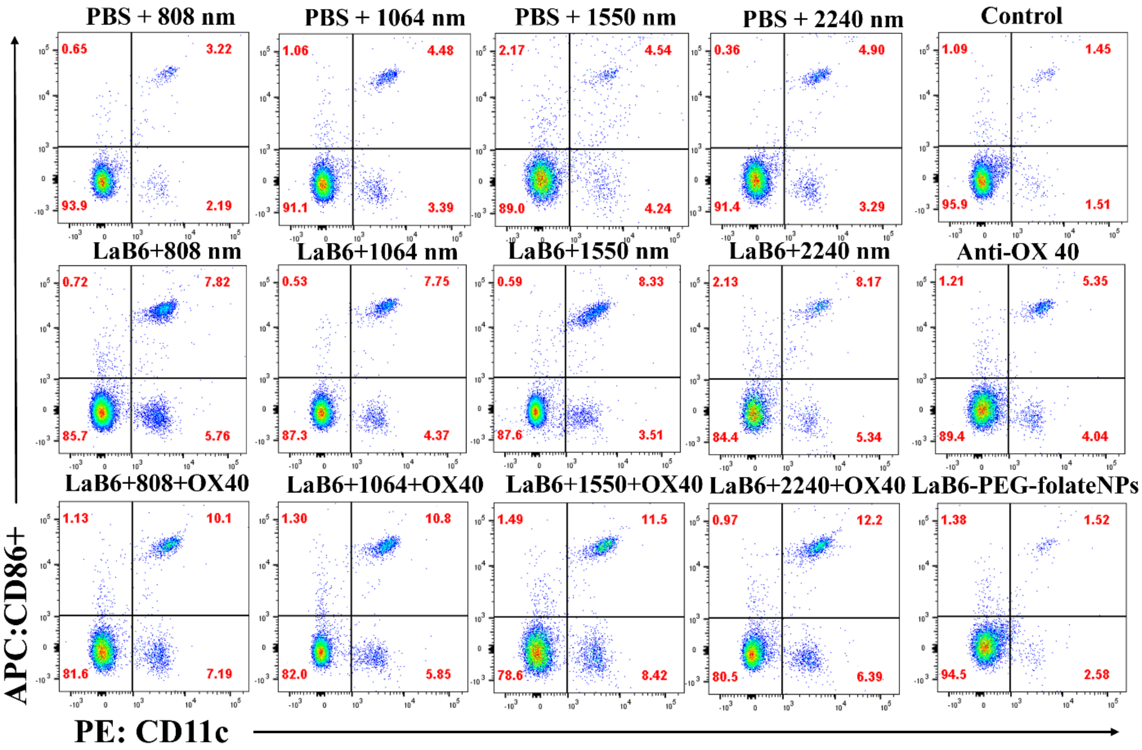

Figure S21. Flow cytometry analysis for DC maturation of B16L6-tumor-bearing mice after staining with anti-CD11c and anti-CD86 antibodies.

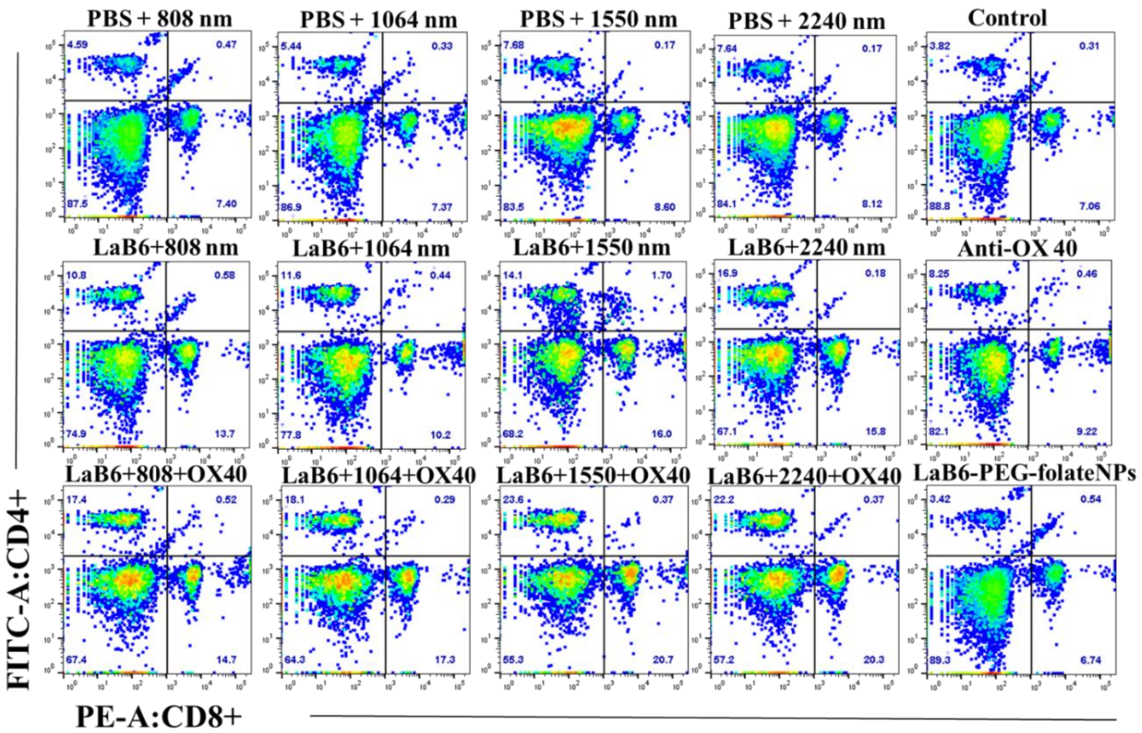

Figure S22: Flow cytometric analysis for expression levels of $CD4^+$, $CD8^+$ and $CD45^+$ T cells in the remote tumors collected from various groups after treatments.

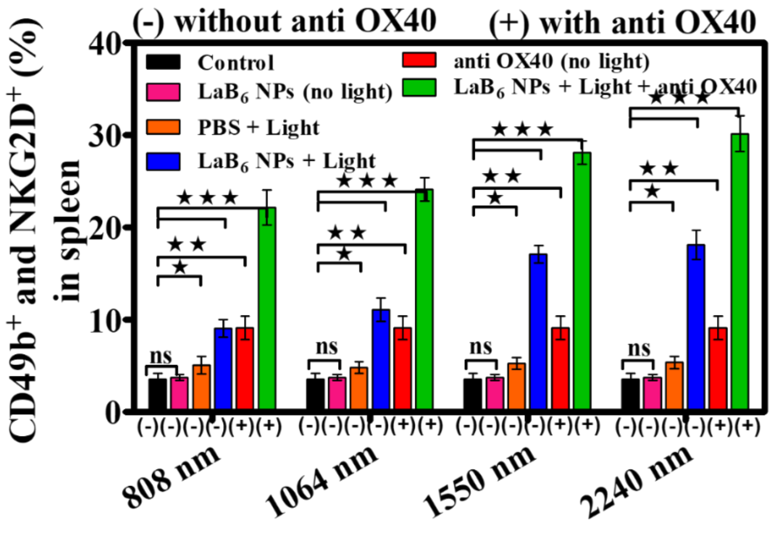

Figure S23. Quantification of the expression level of $CD49b^+NKG2D^+$ for mature NK cells in spleen from different photo-irradiation treamnet groups. *p < 0.05; **p < 0.01; ***p < 0.001.

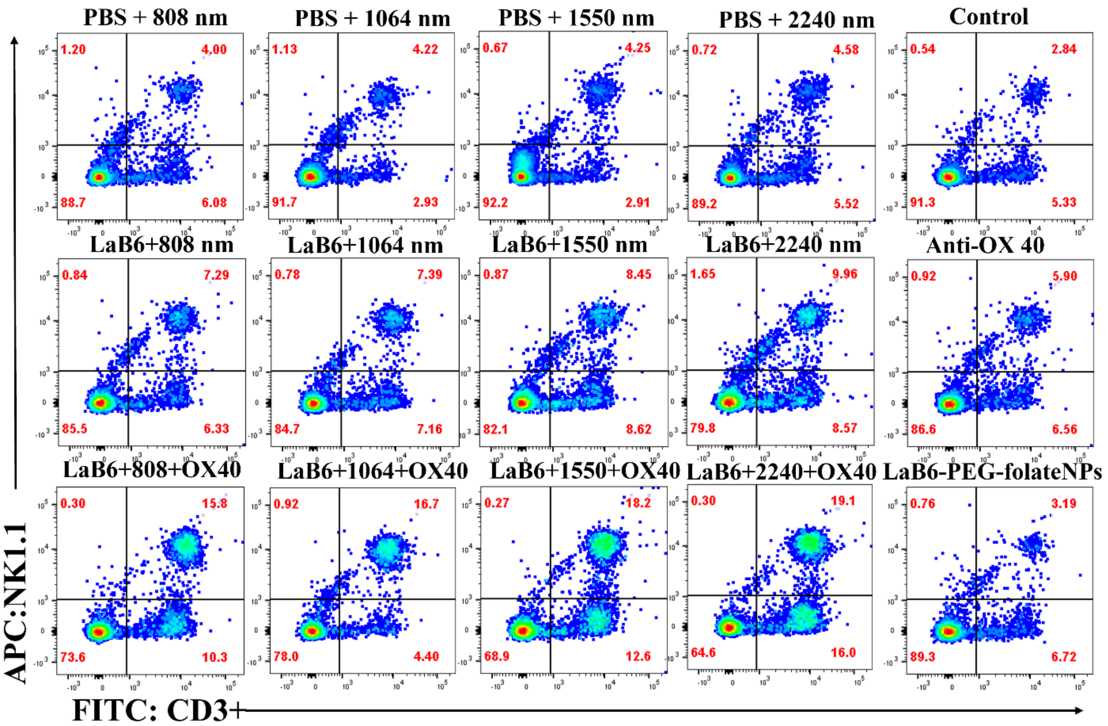

Figure S24. Flow cytometric analysis showing the expression of $CD3^{+}NK1.1^{+}$ cells in the remote tumor.

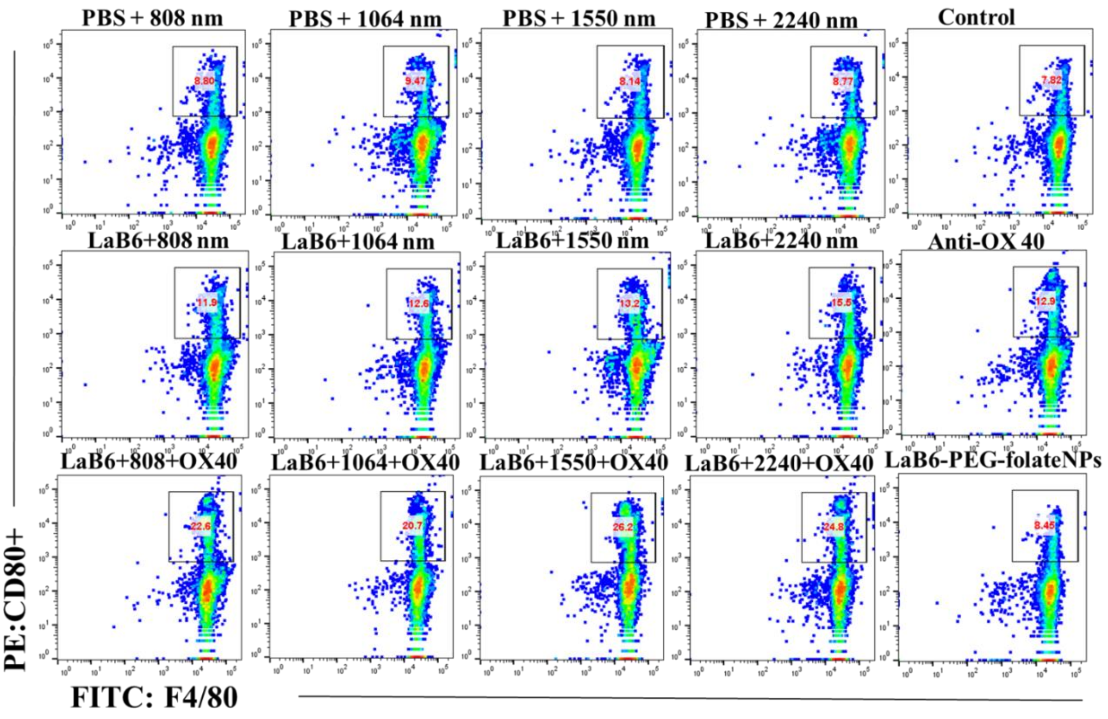

Figure S25. Flow cytometric analysis for expression levels of M1-macrophages $CD86^{+}$, F4/80 in remote tumor.

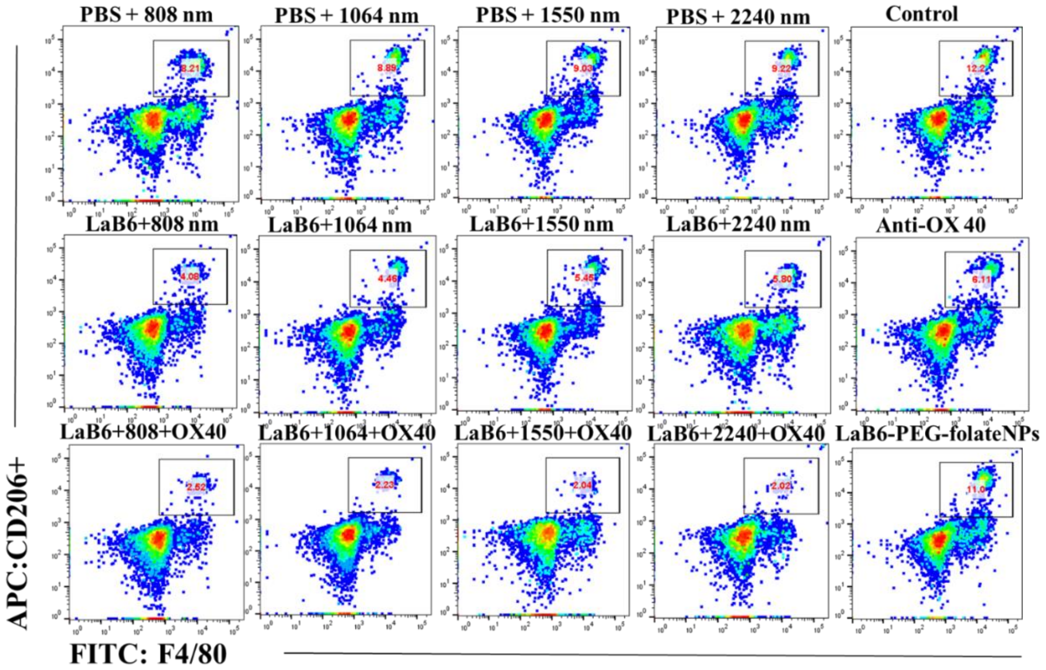

Figure S26. Flow cytometric analysis for expression levels of M2-macrophages CD206^{+}, F4/80 in remote tumor.

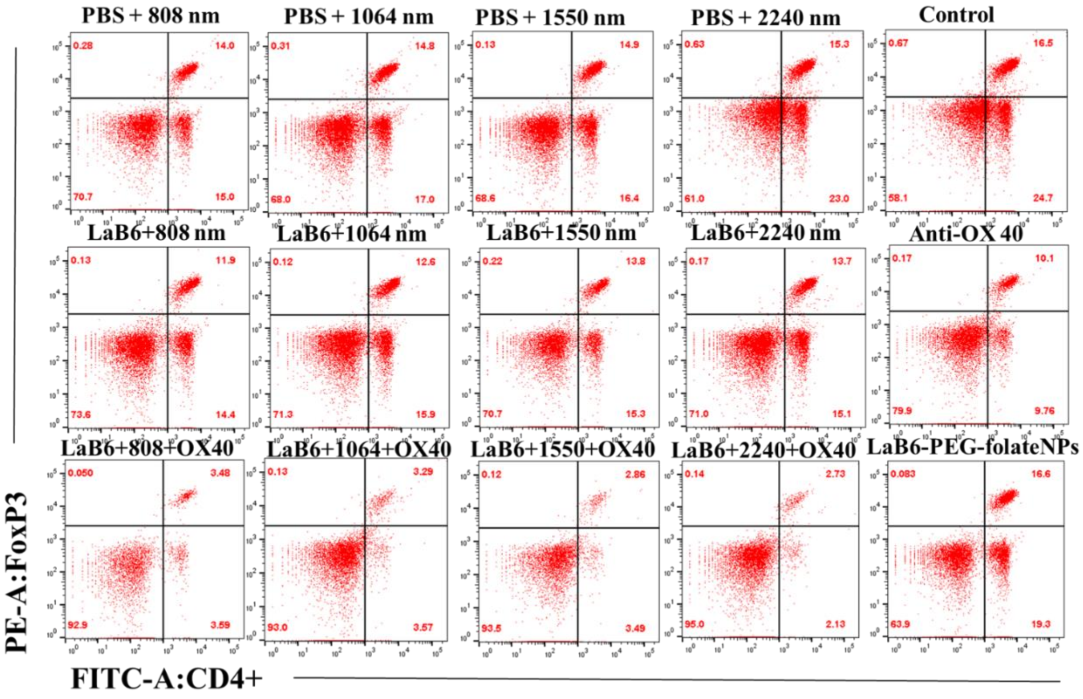

Figure S27. Flow cytometric analysis of lymph nodes for the expression level of CD4^{+}CD25^{+}Foxp3^{+} Treg cells from different photo-irradiation treatment groups.

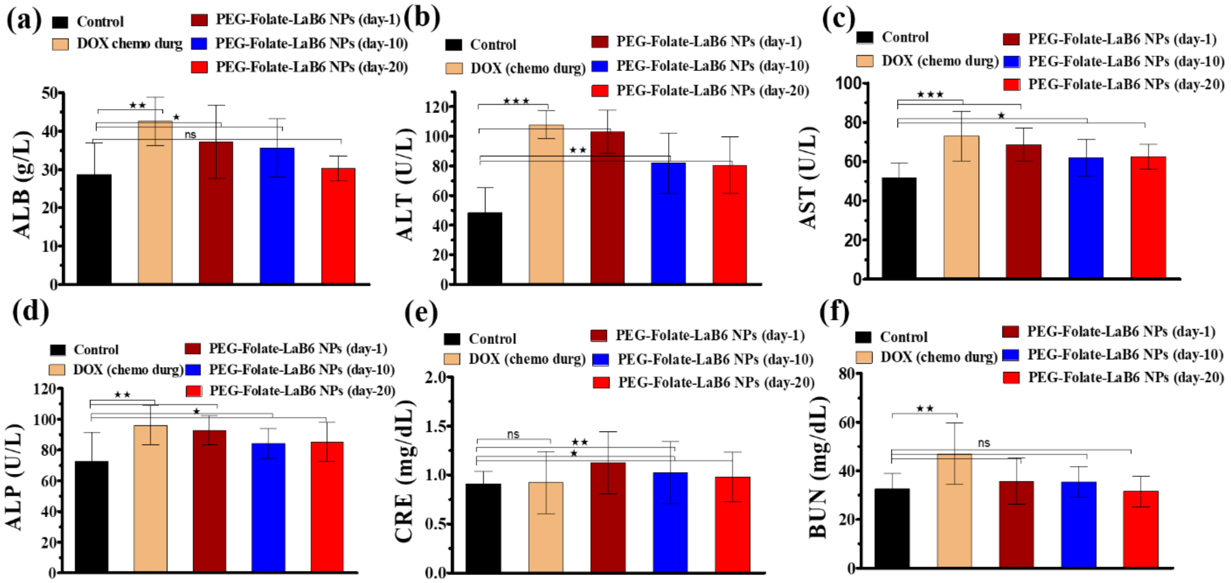

Figure S28. ***In vivo* blood biochemistry evaluation** for LaB_6-PEG-folate NPs (50 mg/kg) and DOX (50 mg/kg, at day 1) in healthy mice at day 1, 10, and 20 post intravenous injection. The levels of (a) ALB, (b) ALT, (c) AST, and (d) ALP associated with hepatic function of liver, whereas (e) BUN, and (f) CRE are related to nephritic function of kidneys, respectively. One-way ANOVA with Tukey's post-hoc test was used to determine statistical significance. $^*P < 0.05$, $^{**}P < 0.01$, $^{***}P < 0.001$.

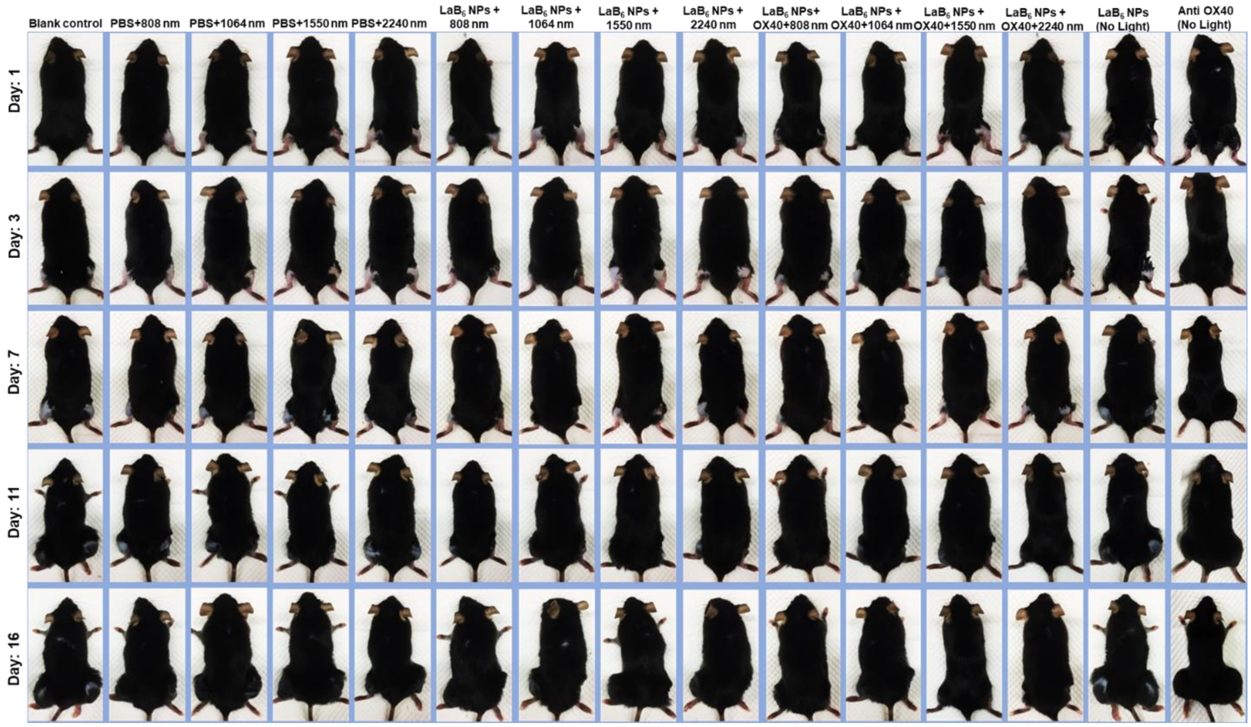

Figure S29. Optical mice images of mice receiving various treatment conditions at day 1, 3, 7, 11 and 16.

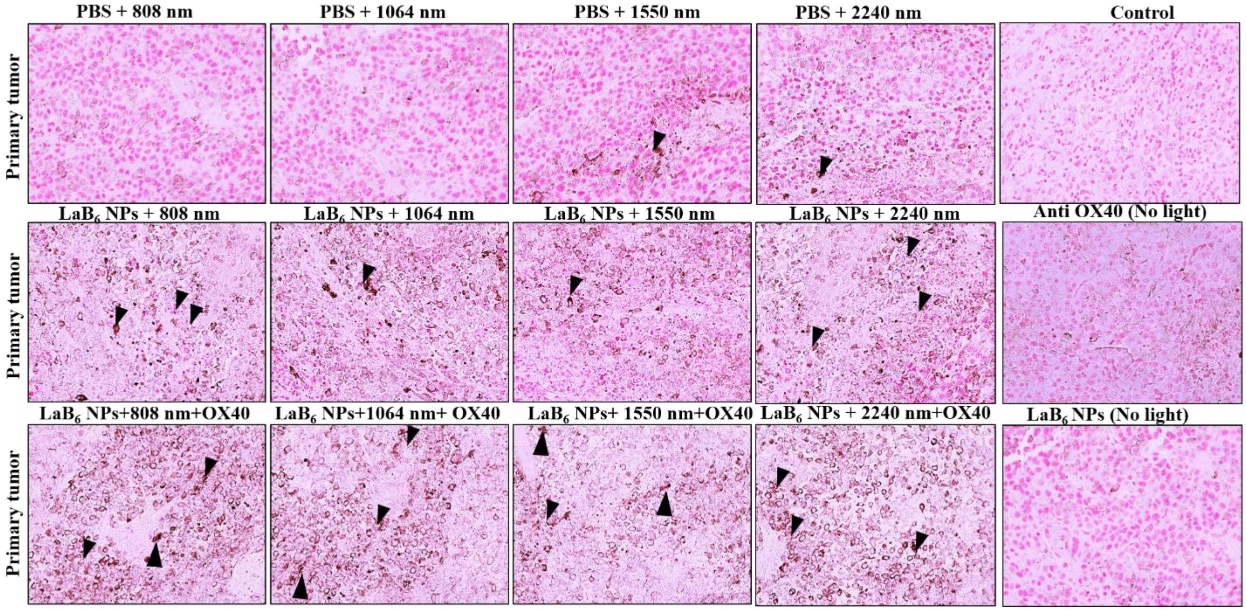

Figure S30. H&E staining of the primary tumor sections of mice treated with LaB_6-PEG-folate+laser light irradiation, including 808 nm, 1064 nm, 1550 nm and 2240 nm NIR light, respectively. The scale bar represents 50 µm. Arrows indicate regions of cancer cell destruction.

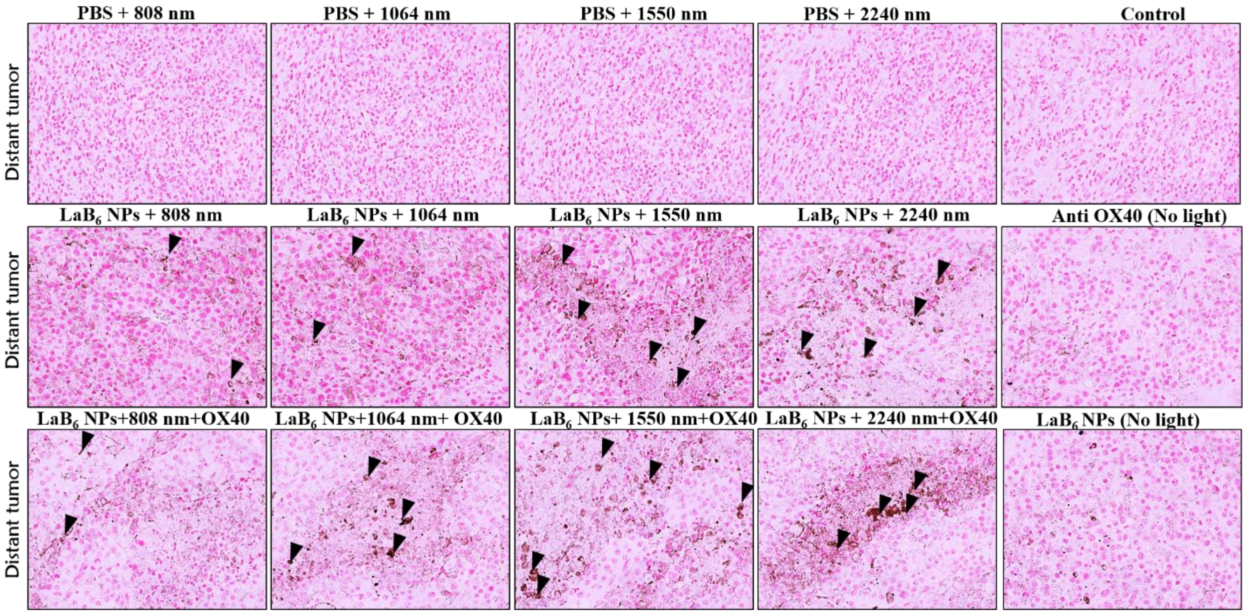

Figure S31. **H&E staining of the remote tumor sections** of mice treated with LaB_6-PEG-folate+laser light irradiation, including 808 nm, 1064 nm, 1550 nm and 2240 nm NIR light, respectively. The scale bar indicates 50 µm**.** Arrows indicate regions of cancer cell destruction.

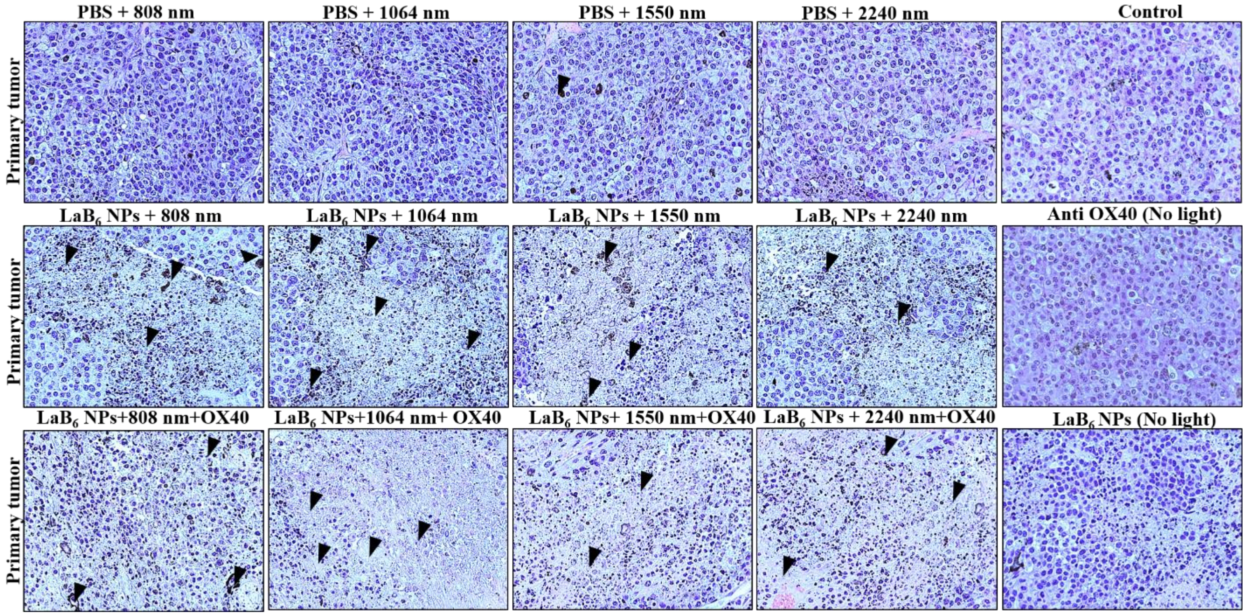

Figure S32. Caspase-3 staining of the primary tumor sections of LaB_6-PEG-folate NPs internalized B16BL6 cells bearing mice under 808 nm, 1064 nm, 1550 nm and 2240 nm photo-irradiation conditions, respectively. The scale bar indicates 50 µm. Arrows indicate cleaved caspase-3-positive cells.

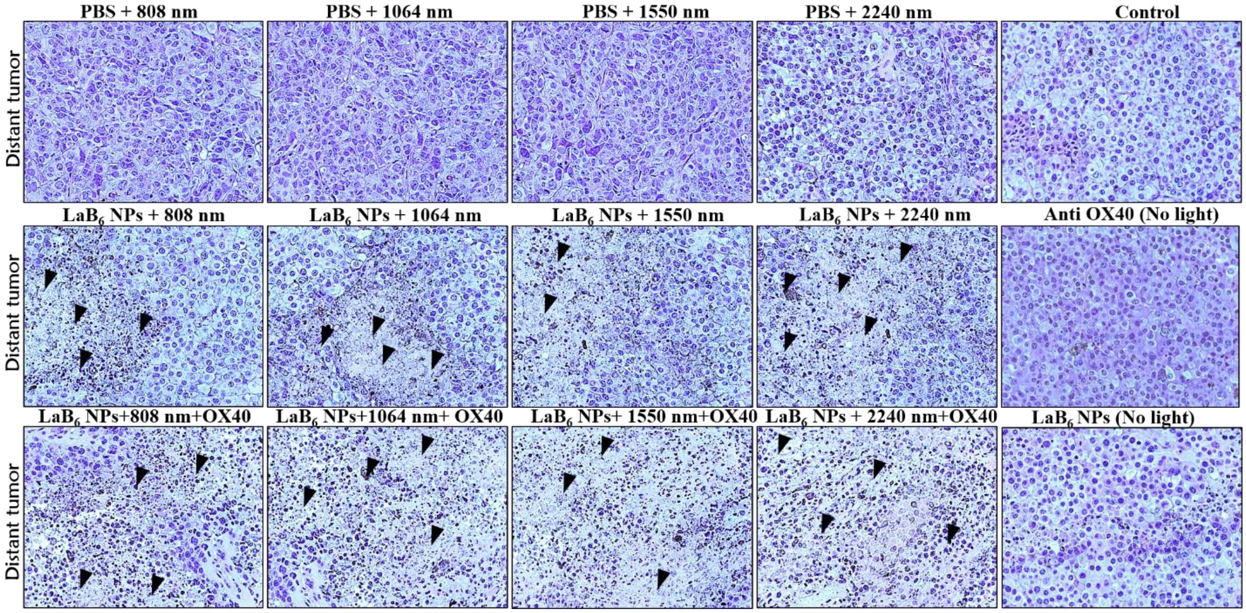

Figure S33. Caspase-3 staining of the remote tumor sections of LaB_6-PEG-folate NPs internalized B16BL6 cells bearing mice under 808 nm, 1064 nm, 1550 nm and 2240 nm photo-irradiation conditions, respectively. The scale bar indicates 50 µm. Arrows indicate cleaved caspase-3-positive cells.

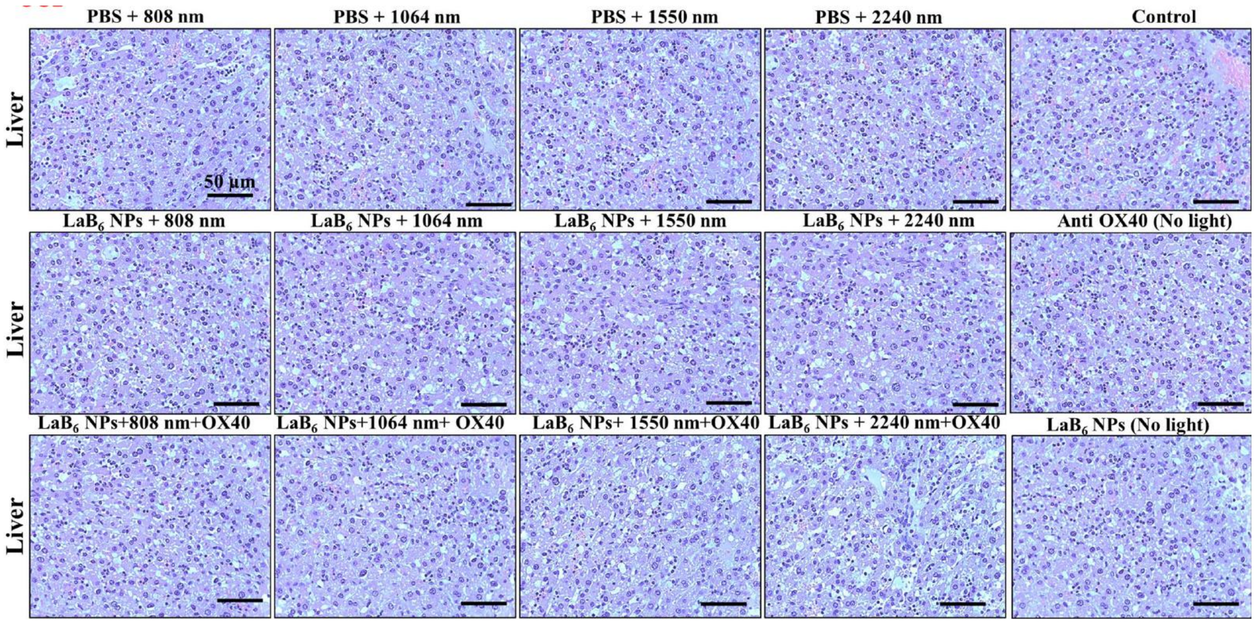

Figure S34. H&E staining images of liver using LaB_6-PEG-folate NPs internalized B16BL6 melanoma cancer cells-bearing mice under 808 nm, 1064 nm, 1550 nm and 2240 nm photo-irradiation conditions, respectively. The scale bar indicates 50 µm.

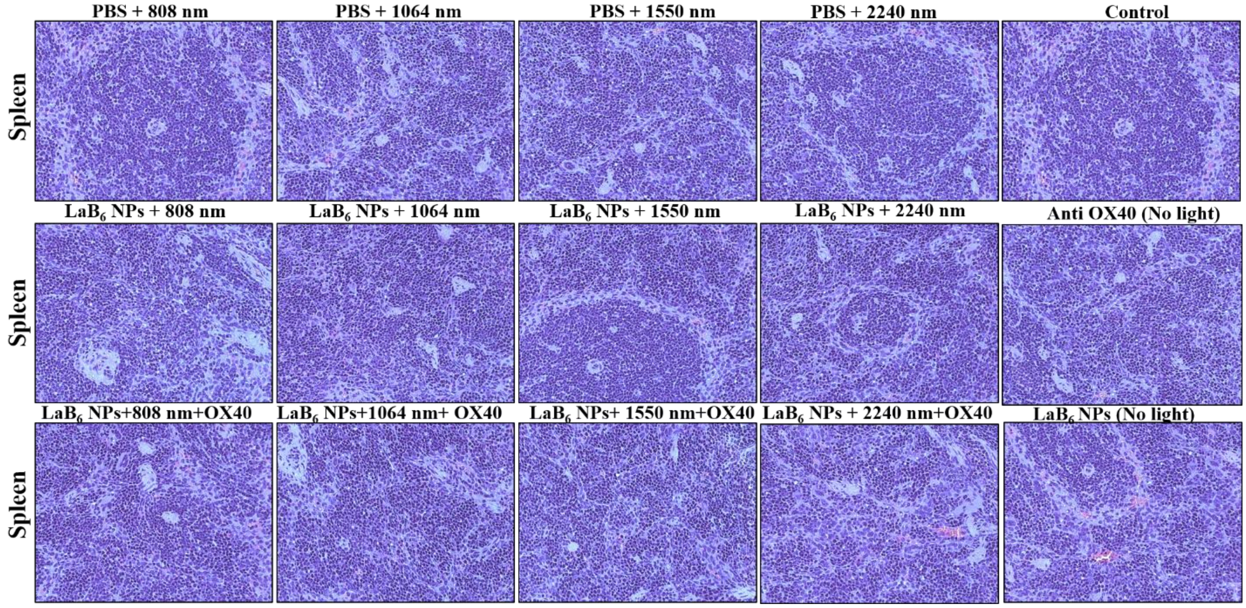

Figure S35. H&E staining images of spleen using LaB_6-PEG-folate NPs internalized B16BL6 cells bearing mice under 808 nm, 1064 nm, 1550 nm and 2240 nm photo-irradiation conditions, respectively. The scale bar indicates 50 µm.

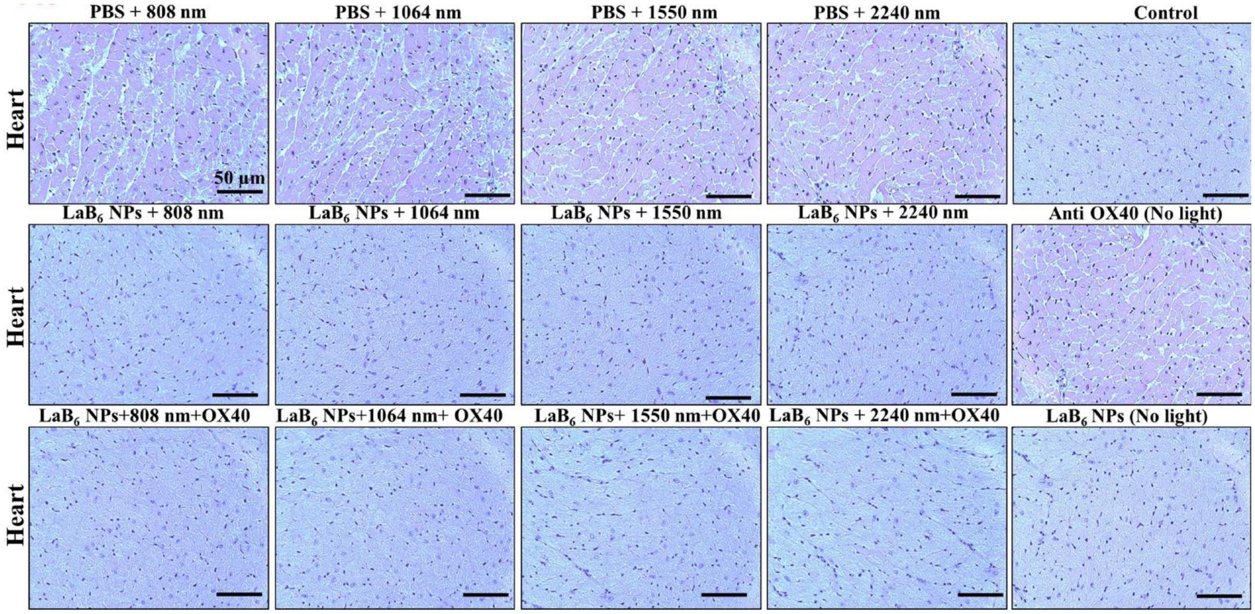

Figure S36. H&E staining images of heart using LaB_6-PEG-folate NPs internalized B16BL6 cells bearing mice under 808 nm, 1064 nm, 1550 nm and 2240 nm photo-irradiation conditions, respectively. The scale bar indicates 50 µm.

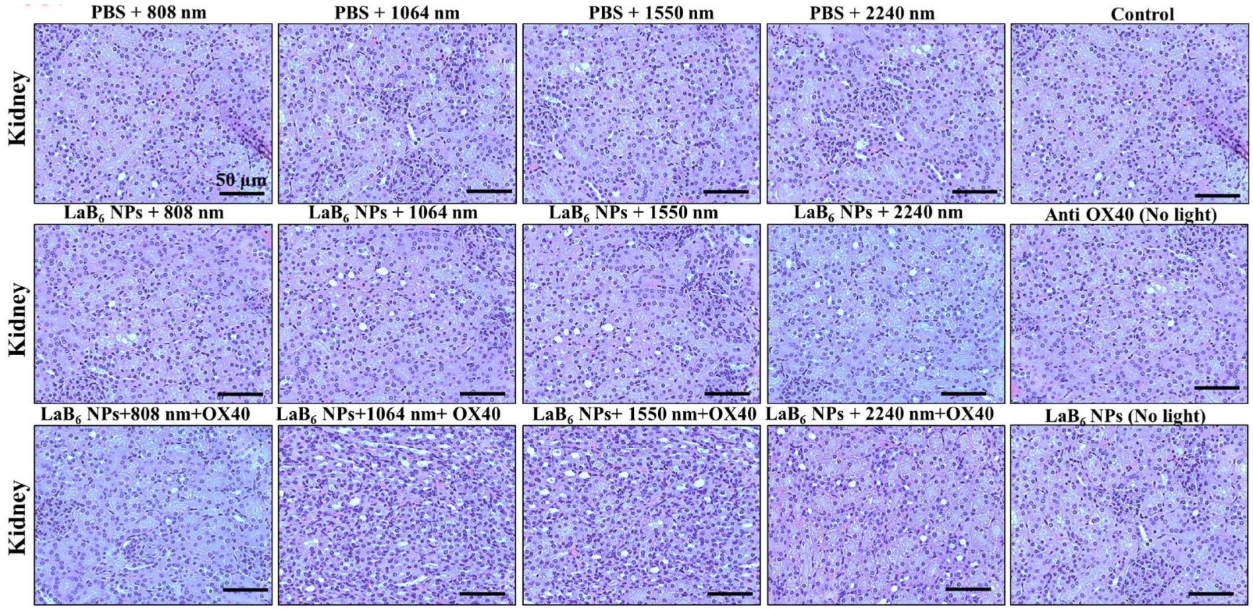

Figure S37. H&E staining images of kidney using LaB_6-PEG-folate NPs internalized B16BL6 cells bearing mice under 808 nm, 1064 nm, 1550 nm and 2240 nm photo-irradiation conditions, respectively. The scale bar indicates 50 µm.

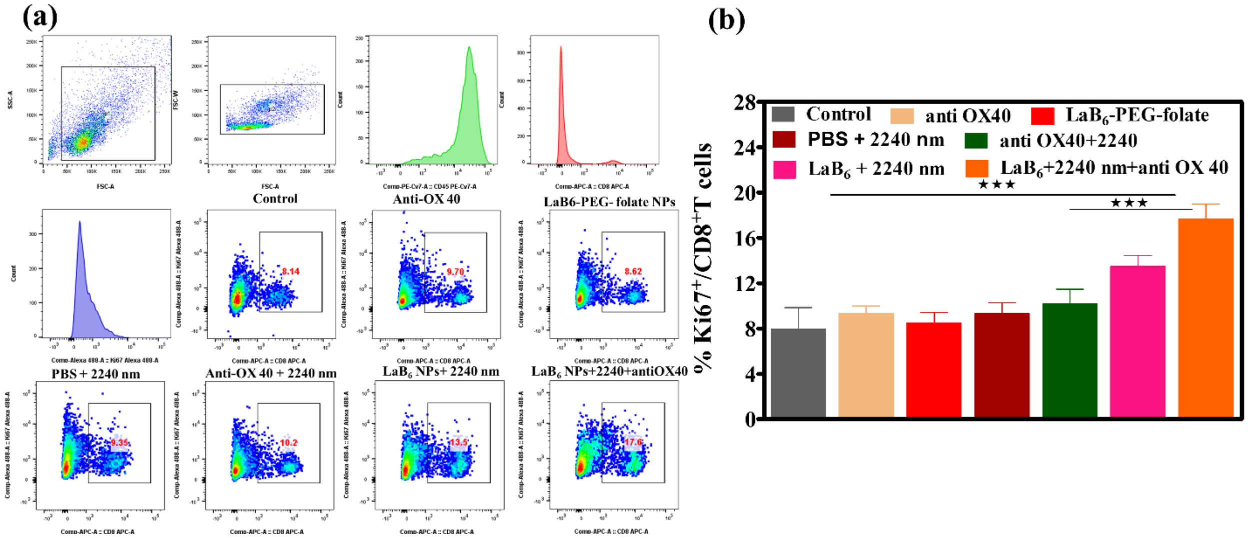

Figure S38. Flow cytometric analysis of T cell proliferation in splenocytes from B16BL6 tumor-bearing mice after different treatment conditions. (a) Representative plots showing gating strategy for $CD45^+$, $CD8^+$, and Ki-67^+ T cells. (b) Proportions of Ki-67^+ proliferating cells within $CD8^+$ T cell population, indicating T cell proliferative activity.

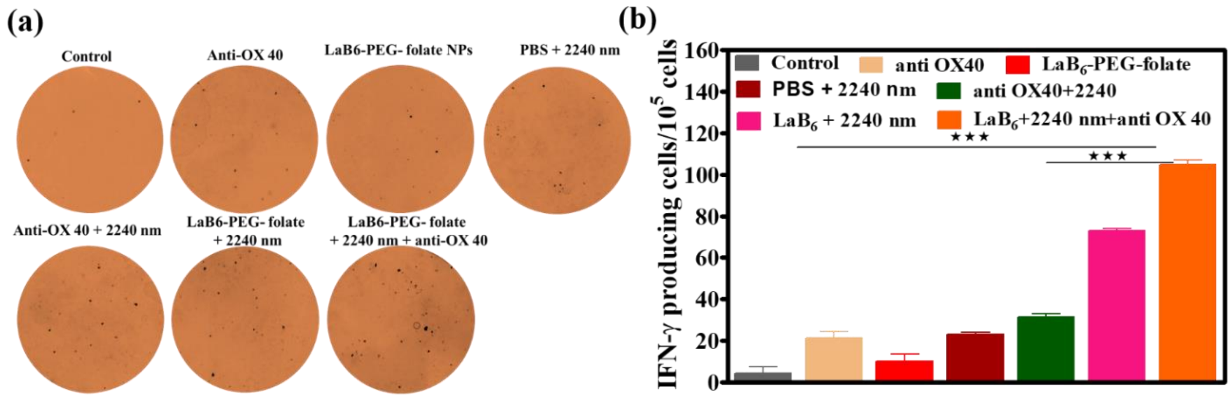

Figure S39. Tumor-specific immune responses. Tumor-specific immune responses. Images and quantitative analysis of ELISPOT assay was performed to detect IFN-γ producing T cells. Data are expressed as means ± s.d. (n = 2). *P < 0.05, **P < 0.01, and ***P < 0.001 from control.

Table S1. Quantification of key antitumor-related cytokines (pg/mL) across different NIR light treatment groups. Statistical significance was determined using one-way ANOVA followed by Tukey's post-hoc test. *P < 0.05, **P < 0.01, ***P < 0.001, comparing treatment groups to the control and among selected group pairs as indicated.

Cytokine	LaB_6-PEG - folate+808 nm+ anti OX40 NIR-I (pg/mL)	LaB_6-PEG- folate+1064 nm+ anti OX40 NIR-II (pg/mL)	LaB_6-PEG- folate+1550 nm +anti OX40 NIR-III (pg/mL)	LaB_6-PEG- folate+2240 nm +anti OX40 NIR-IV (pg/mL)	Significant Differences
TNF-α	280 ± 8	303 ± 10	385 ± 15	400 ± 12	NIR-III/IV > NIR-I/II ($p < 0.01$)
IFN-γ	762 ± 14	780 ± 12	942 ± 13	970 ± 15	NIR-III/IV > NIR-I/II ($p < 0.01$)
IL-2	38 ± 3	42 ± 4	55 ± 5	57 ± 5	NIR-III/IV > NIR-I/II ($p < 0.001$)
IL-12	81 ± 6	93 ± 7	121 ± 9	125 ± 10	NIR-III/IV > NIR-I/II ($p < 0.01$)

Table S2. Raw serum cytokine concentrations (pg/mL) for TNF-α, IFN-γ, IL-2, and IL-12 measured in B16BL6 tumor-bearing mice across different treatment groups. Values represent mean ± SD (n = 2 per group). Data shown as mean ± half-range; n = 2 replicates per condition.

**Group**	**Serum TNF-α (pg/mL)**	**Serum IFN-γ (pg/mL)**	**Serum IL-2 (pg/mL)**	**Serum IL-12 (pg/mL)**
Control	69.75 ± 3.5	349.25 ± 1.5	7.9 ± 0.2	20.25 ± 0.5
PBS + 808 nm	71.15 ± 1.7	392.75 ± 5.5	11.85 ± 0.3	26.25 ± 0.5
PBS + 1064 nm	87.7 ± 0.6	422.25 ± 4.5	13.675 ± 0.35	29.25 ± 0.5
PBS + 1550 nm	94.5 ± 3	434.25 ± 3.5	13.85 ± 0.1	30.25 ± 0.5
PBS + 2240 nm	85.25 ± 2.5	394 ± 2	12.95 ± 0.3	30.25 ± 0.5
LaB_6-PEG folate NPs + 808 nm	154.5 ± 1	483.5 ± 3	19.8 ± 0.4	50.25 ± 0.5
LaB_6-PEG folate NPs + 1064 nm	222.5 ± 5	504 ± 8	21.175 ± 0.35	54.25 ± 0.5
LaB_6-PEG folate NPs + 1550 nm	244.6 ± 1.8	738.5 ± 7	26.625 ± 0.25	68.25 ± 0.5
LaB_6-PEG folate NPs + 2240 nm	233.65 ± 2.7	707.5 ± 55	23.5 ± 1	79.25 ± 0.5
anti-OX 40	151 ± 18	491.75 ± 3.5	21.2 ± 1.6	35.75 ± 0.5
LaB_6-PEG folate NPs	70.3 ± 0.6	381 ± 2	7.7 ± 0.2	19.8 ± 0.3
LaB_6-PEG folate NPs + 808 nm + anti OX 40	279.5 ± 1	765 ± 6	38.4 ± 0.4	80.85 ± 0.4
LaB_6-PEG folate NPs + 1064 nm + anti OX 40	305 ± 4	785 ± 10	42.15 ± 0.3	93.5 ± 1
LaB_6-PEG folate NPs + 1550 nm + anti OX 40	388.5 ± 7	943.75 ± 3.5	55.35 ± 0.7	120.5 ± 1
LaB_6-PEG folate NPs + 2240 nm + anti OX 40	397.5 ± 5	878 ± 4	55.5 ± 7	124.65 ±0.7

Table S3. Chemical and reagent.

Antibody	Lot number	Manufacturer
1-(3-dimethylaminopropyl)-3-ethylcarbodiimide hydrochloride	10195051	Alpha Aesar
Sodium borohydride	Y09H029	Alpha Aesar
Sodium Chloride	WXBC7715V	VETECH
Potassium chloride	KBP202A	Showa
Citric acid	KJR005C	Showa
Lanthanum chloride hydrated	20211-76-1	Sigma aldrich
RPMI Medium 1640	2725256	Gibco
Dulbecco's Modified Eagle Medium	2985148	Gibco
4′,6-diamidino-2-phenylindole	2217039	Invitrogen
EDTA	2946648	Invitrogen
Lithium Heparin	24032851	Greiner
APF	10157	LSBio
H2DCFDA(2,7-dichlorofluorescin diacetate)	2032450	Invitrogen
5,5-Dimethyyl-1-pyrroline-N-oxide	L22M	Matrix

Table S4. Flow-cytometry antibodies.

Antibody	Lot number	Manufacturer
Alexa Fluor 488 anti-Hsp70 Antibody	B266951	BioLegend, Inc.
Propidium Iodide Staining Solution	2111675	BD biosciences
APC Annexin V	B434233	BioLegend, Inc.
Calcein AM	582423	Medchem Express
Singlet Oxygen Sensor Green	1735084	Life techlogies
FITC anti-mouse CD80 Antibody	9276231	BD biosciences
APC anti-mouse CD86 Antibody	9301674	BD biosciences
PE-Cy anti-mouse CD11c Antibody	9345738	BD biosciences
APC anti-mouse CD45 Antibody	8277680	BD biosciences
FITC anti-mouse CD4 Antibody	9156805	BD biosciences
PE anti-mouse CD8a Antibody	9204340	BD biosciences
PE anti-mouse CD25 Antibody	0000073847	BD biosciences
FITC anti-mouse CD3 Antibody	3150826	BD biosciences
Cy5 Goat Anti-Mouse IgG	2286327	invitrogen
FITC anti-mouse F4/80 Recombinant Antibody	2732768	invitrogen
APC anti-mouse CD206 (MMR) Antibody	2763949	invitrogen
Purified Rat Anti-Mouse CD16/CD32	9297201	BD Pharmingen
APC anti-mouse Ki-67 Antibody	B434167	Bio Legend
Anti-Mo NK1.1	2791113	invitrogen
PE anti-mouse CD80 Antibody	2630427	invitrogen
FoxP3 Rabbit mAb	D6O8R	Cell signaling Techlogy

Table S5. ELISA (Enzyme-Linked ImmunoSorbent Assay) analysis specific proteins and antigens.

Antibody	Lot number	Manufacturer
Mouse TNF-alpha ELISA Kit	P190320	R&D Systems
Mouse IFN-gamma ELISA Kit	P138913	R&D Systems
Mouse IL-2 ELISA Kit	P226350	R&D Systems
Mouse IL-12 ELISA Kit	20240219	Sunlong Biotech Co., Ltd
Mouse IL-10 ELISA Kit	20240818	Sunlong Biotech Co., Ltd
Mouse TGF-beta ELISA Kit	20240219	Sunlong Biotech Co., Ltd
Mouse IFN-gamma ELISpot Kit	EL285	R&D Systems
Mouse/Rat HMGB1 ELISA Kit	130814	Arigo
ATP Assay Kit-Luminescence	WU821	Dojindo Laboratories
Amplex Red Hydrogen Peroxide/Peroxidase Assay Kit	2201580	Invitrogen
Thiazolyl blue tetrazolium bromide	10169095	Alfa Aesar

Supporting References:

S1. Wilkinson F, Helman WP, Ross AB.. Quantum Yields for the Photosensitized Formation of the Lowest Electronically Excited Singlet State of Molecular Oxygen in Solution. *J. Phys. Chem. Ref. Data* 1993, 22, 113–262.

S2. Gao RM, Stark J, Bahnemann DW, Rabani J. Quantum yields of hydroxyl radicals in illuminated TiO_2 nanocrystallite layers. J. Photochem. PhotoBiol. A: Chem.2002, 148, 387-391.
